# A model of gene-gene and gene-environment interactions and its implications for targeting environmental interventions by genotype

**DOI:** 10.1186/1742-4682-3-35

**Published:** 2006-10-09

**Authors:** Helen M Wallace

**Affiliations:** 1 GeneWatch UK, The Mill House, Tideswell, Buxton, Derbyshire, SK17 8LN, UK

## Abstract

**Background:**

The potential public health benefits of targeting environmental interventions by genotype depend on the environmental and genetic contributions to the variance of common diseases, and the magnitude of any gene-environment interaction. In the absence of prior knowledge of all risk factors, twin, family and environmental data may help to define the potential limits of these benefits in a given population. However, a general methodology to analyze twin data is required because of the potential importance of gene-gene interactions (epistasis), gene-environment interactions, and conditions that break the 'equal environments' assumption for monozygotic and dizygotic twins.

**Method:**

A new model for gene-gene and gene-environment interactions is developed that abandons the assumptions of the classical twin study, including Fisher's (1918) assumption that genes act as risk factors for common traits in a manner necessarily dominated by an additive polygenic term. Provided there are no confounders, the model can be used to implement a top-down approach to quantifying the potential utility of genetic prediction and prevention, using twin, family and environmental data. The results describe a solution space for each disease or trait, which may or may not include the classical twin study result. Each point in the solution space corresponds to a different model of genotypic risk and gene-environment interaction.

**Conclusion:**

The results show that the potential for reducing the incidence of common diseases using environmental interventions targeted by genotype may be limited, except in special cases. The model also confirms that the importance of an individual's genotype in determining their risk of complex diseases tends to be exaggerated by the classical twin studies method, owing to the 'equal environments' assumption and the assumption of no gene-environment interaction. In addition, if phenotypes are genetically robust, because of epistasis, a largely environmental explanation for shared sibling risk is plausible, even if the classical heritability is high. The results therefore highlight the possibility – previously rejected on the basis of twin study results – that inherited genetic variants are important in determining risk only for the relatively rare familial forms of diseases such as breast cancer. If so, genetic models of familial aggregation may be incorrect and the hunt for additional susceptibility genes could be largely fruitless.

## Background

Some geneticists have predicted a genetic revolution in healthcare: involving a future in which individuals take a battery of genetic tests, at birth or later in life, to determine their individual 'genetic susceptibility' to disease [1,2]. In theory, once the risk of particular combinations of genotype and environmental exposure is known, medical interventions (including lifestyle advice, screening or medication) could then be targeted at high-risk groups or individuals, with the aim of preventing disease [3].

However, there are also many critics of this strategy, who argue that it is likely to be of limited benefit to health [4-8]. One area of debate concerns the proportion of cases of a given common disease that might be avoided by targeting environmental or lifestyle interventions to those at high genotypic risk. Known genetic risk factors have to date shown limited utility in this respect [9]. However, some argue that combinations of multiple genetic risk factors may prove more useful in the future [10].

There are two possible approaches to considering this issue. The 'bottom-up' approach seeks to identify individual genetic and environmental risk factors and their interactions and quantify the risks. However, this approach is limited by the difficulties in establishing the statistical validity of genetic association studies and of quantifying gene-gene and gene-environment interactions: see, for example, [11-14].

A 'top-down' approach instead considers risks at the population level using twin and family studies and data on the importance of environmental factors in determining a trait. However, analysis of twin data is usually limited by the assumptions made in the classical twin study [15], including that: (i) there are no gene-gene interactions (epistasis); (ii) there are no gene-environment interactions; (iii) the effects of environmental factors shared by twins are independent of zygosity (the 'equal environments' assumption). These assumptions have all been individually explored and shown to be important in influencing the conclusions drawn from twin and family data [16-18]. In addition, the magnitude of any gene-environment interaction is critically important in determining the utility of targeting environmental interventions by genotype [19]. Although a general methodology to analyze twin data without making these assumptions has been developed, the algebra becomes intractable once multiple loci are involved [17]. This is problematic because, for common diseases, the impacts of multiple genetic variants, and potentially the whole genetic sequence, on disease susceptibility (here called 'genotypic risk') may be important.

The four-category model of population risks developed by Khoury and others [19] is a useful starting point for a top-down analysis of genetic prediction and prevention. It allows the merits of a targeted intervention strategy (which seeks to reduce the exposure of the high-risk genotype group only) to be explored, and can readily be extended to include more than four risk categories [10]. However, this model's use to date has been limited to bottom-up consideration of single genetic variants or to studying hypothetical examples of multiple variants. The four-category model is limited by the assumption of no confounders, which means it is applicable to only a subset of possible models of gene-gene and gene-environment interaction. However, situations where the 'no confounders' assumption is valid are arguably most likely to be of relevance to public health.

The aim of this paper is to combine the four-category model with population level data from twin, family and environmental studies, without adopting the classical twin model assumptions. This model of gene-gene and gene-environment interactions is then used to implement a 'top-down' approach to quantifying the utility of genetic 'prediction and prevention'.

## Method

### The four-category model

**Figure 1:**
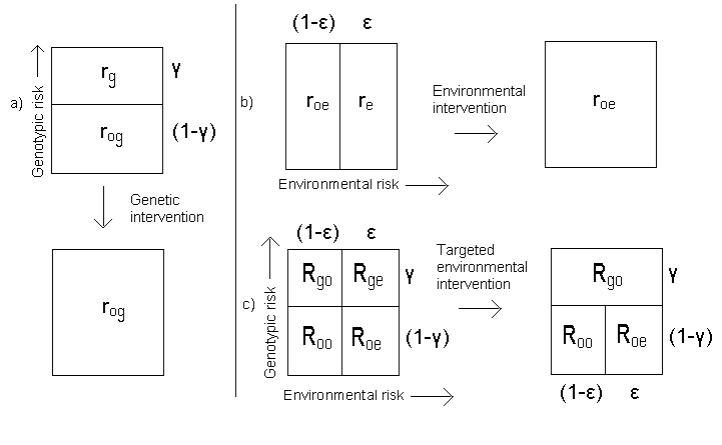
**The four-category model**. A population divided into: (a) high and low genotypic risk categories (r_g _and r_og_); (b) high and low environmental risk categories (r_e _and r_oe_); (c) four categories based on combined genotypic and environmental risk.

Consider a population divided into genotypic or environmental risk categories for a given trait (Figure 1a and 1b). The fraction of the population in the 'high environmental risk group' (designated by subscript e) is ε, and this subpopulation is at risk r_e_. The remainder of the population is at risk r_oe_. The fraction of the population in the 'high genotypic risk' group (designated by the subscript g) is γ, and this subpopulation is at risk r_g_, with the remainder of the population at risk r_og_. The total risk r_t _for this trait in this population is then given by:

*r*_*t *_= γ*r*_*g *_+ (1-γ)*r*_*og *_    (1)

or by:

*r*_*t *_= ε*r*_e _+ (1-ε)*r*_*oe *_    (2)

The same population can alternatively be divided into four categories, making a four-category model (Figure 1c)) with risks R_oo_, R_oe_, R_go _and R_ge_. Table 1 shows the risk categories in this model.

**Table 1:** The four category model: risks and cases for a population of size N.

**Category**	**Risk of being in category**	**Number of people in category**	**Number of cases in category**
**ge **(high-risk genotype/high-risk exposure)	R_ge_	γεN	γε R_ge_N
**go **(high-risk genotype/low-risk exposure)	R_go_	γ (1-ε)N	γ (1-ε)R_go_N
**oe **(low-risk genotype/high-risk exposure)	R_oe_	ε (1-γ)N	ε (1-γ)R_oe_N
**oo **(low-risk genotype/low-risk exposure)	R_oo_	(1-ε) (1-γ)N	(1-ε) (1-γ)R_oo_N

**Total**		N	r_t_N

The risks are related to the previous definitions by:

*r*_*g *_= *εR*_*ge *_+ (1-*ε*) *R*_*go *_    (3)

*r*_*og *_= *εR*_*oe *_+ (1-*ε*) *R*_*oo *_    (4)

*r*_e _= *γR*_*ge *_+ (1-*γ*) *R*_*oe *_    (5)

*r*_*oe *_= *γR*_*og *_+ (1-*γ*) *R*_*oo *_    (6)

The category risks R remain constant in different populations (i.e. as ε and γ vary), provided there are no confounders. This assumption restricts the model to special cases of gene-gene and gene-environment interaction. Note that for a single genetic variant, r_g _corresponds to the penetrance of the variant, and that in general (provided R_ge _≠ R_go_) this varies with the proportion of the population in the high exposure group, ε, as has been observed [20,21].

The total risk for the given trait is given by:

*r*_*t *_= *γεR*_*ge *_+ *γ*(1-*ε*)*R*_*go *_+ *ε*(1-*γ*)*R*_*oe *_+ (1-*ε*)(1-*γ*)*R*_*oo *_    (7)

The subpopulation of cases has different characteristics from the general population: for example, it contains a higher proportion of people from the 'ge' subgroup. The relative risk for a person drawn randomly from a subpopulation with the same genotypic and environmental characteristics as the cases, RR^cases^, is given by the sum of the relative risks for each category shown in Table 1:

RRcases=γεRge2+γ(1−ε)Rgo2+ε(1−γ)Roe2+(1−ε)(1−γ)Roo2rt2     (8)
 MathType@MTEF@5@5@+=feaafiart1ev1aaatCvAUfKttLearuWrP9MDH5MBPbIqV92AaeXatLxBI9gBaebbnrfifHhDYfgasaacH8akY=wiFfYdH8Gipec8Eeeu0xXdbba9frFj0=OqFfea0dXdd9vqai=hGuQ8kuc9pgc9s8qqaq=dirpe0xb9q8qiLsFr0=vr0=vr0dc8meaabaqaciaacaGaaeqabaqabeGadaaakeaacqWGsbGucqWGsbGudaahaaWcbeqaaiabdogaJjabdggaHjabdohaZjabdwgaLjabdohaZbaakiabg2da9maalaaabaGaeq4SdCMaeqyTduMaemOuai1aa0baaSqaaiabdEgaNjabdwgaLbqaaiabikdaYaaakiabgUcaRiabeo7aNjabcIcaOiabigdaXiabgkHiTiabew7aLjabcMcaPiabdkfasnaaDaaaleaacqWGNbWzcqWGVbWBaeaacqaIYaGmaaGccqGHRaWkcqaH1oqzcqGGOaakcqaIXaqmcqGHsislcqaHZoWzcqGGPaqkcqWGsbGudaqhaaWcbaGaem4Ba8MaemyzaugabaGaeGOmaidaaOGaey4kaSIaeiikaGIaeGymaeJaeyOeI0IaeqyTduMaeiykaKIaeiikaGIaeGymaeJaeyOeI0Iaeq4SdCMaeiykaKIaemOuai1aa0baaSqaaiabd+gaVjabd+gaVbqaaiabikdaYaaaaOqaaiabdkhaYnaaDaaaleaacqWG0baDaeaacqaIYaGmaaaaaOGaaCzcaiaaxMaadaqadaqaaiabiIda4aGaayjkaiaawMcaaaaa@7152@

Similarly, the relative risk for a person drawn randomly from a subpopulation with the same genotypic characteristics as the cases (but with the environmental characteristics of the general population) is:

RRgencases=γrg2+(1−γ)rog2rt2     (9)
 MathType@MTEF@5@5@+=feaafiart1ev1aaatCvAUfKttLearuWrP9MDH5MBPbIqV92AaeXatLxBI9gBaebbnrfifHhDYfgasaacH8akY=wiFfYdH8Gipec8Eeeu0xXdbba9frFj0=OqFfea0dXdd9vqai=hGuQ8kuc9pgc9s8qqaq=dirpe0xb9q8qiLsFr0=vr0=vr0dc8meaabaqaciaacaGaaeqabaqabeGadaaakeaacqWGsbGucqWGsbGudaqhaaWcbaGaem4zaCMaemyzauMaemOBa4gabaGaem4yamMaemyyaeMaem4CamNaemyzauMaem4CamhaaOGaeyypa0ZaaSaaaeaaiiGacqWFZoWzcqWGYbGCdaqhaaWcbaGaem4zaCgabaGaeGOmaidaaOGaey4kaSIaeiikaGIaeGymaeJaeyOeI0Iae83SdCMaeiykaKIaemOCai3aa0baaSqaaiabd+gaVjabdEgaNbqaaiabikdaYaaaaOqaaiabdkhaYnaaDaaaleaacqWG0baDaeaacqaIYaGmaaaaaOGaaCzcaiaaxMaadaqadaqaaiabiMda5aGaayjkaiaawMcaaaaa@5403@

The relative risk for a person drawn randomly from a subpopulation with the same environmental characteristics as the cases (but with the genotypic characteristics of the general population) is:

RRenvcases=εre2+(1−ε)roe2rt2     (10)
 MathType@MTEF@5@5@+=feaafiart1ev1aaatCvAUfKttLearuWrP9MDH5MBPbIqV92AaeXatLxBI9gBaebbnrfifHhDYfgasaacH8akY=wiFfYdH8Gipec8Eeeu0xXdbba9frFj0=OqFfea0dXdd9vqai=hGuQ8kuc9pgc9s8qqaq=dirpe0xb9q8qiLsFr0=vr0=vr0dc8meaabaqaciaacaGaaeqabaqabeGadaaakeaacqWGsbGucqWGsbGudaqhaaWcbaGaemyzauMaemOBa4MaemODayhabaGaem4yamMaemyyaeMaem4CamNaemyzauMaem4CamhaaOGaeyypa0ZaaSaaaeaaiiGacqWF1oqzcqWGYbGCdaqhaaWcbaGaemyzaugabaGaeGOmaidaaOGaey4kaSIaeiikaGIaeGymaeJaeyOeI0Iae8xTduMaeiykaKIaemOCai3aa0baaSqaaiabd+gaVjabdwgaLbqaaiabikdaYaaaaOqaaiabdkhaYnaaDaaaleaacqWG0baDaeaacqaIYaGmaaaaaOGaaCzcaiaaxMaadaqadaqaaiabigdaXiabicdaWaGaayjkaiaawMcaaaaa@54F7@

### Population attributable fractions

Provided there are no confounders, the population attributable fraction (PAF^E^_e_) due to the presence of the high exposure (E) in the high exposure population subgroup (e) may be defined as:

PAFeE=ε(re−roe)rt=ε{γ(Rge−Rgo)+(1−γ)(Roe−Roo)}/rt     (11)
 MathType@MTEF@5@5@+=feaafiart1ev1aaatCvAUfKttLearuWrP9MDH5MBPbIqV92AaeXatLxBI9gBaebbnrfifHhDYfgasaacH8akY=wiFfYdH8Gipec8Eeeu0xXdbba9frFj0=OqFfea0dXdd9vqai=hGuQ8kuc9pgc9s8qqaq=dirpe0xb9q8qiLsFr0=vr0=vr0dc8meaabaqaciaacaGaaeqabaqabeGadaaakeaacqWGqbaucqWGbbqqcqWGgbGrdaqhaaWcbaGaemyzaugabaGaemyraueaaOGaeyypa0ZaaSaaaeaaiiGacqWF1oqzcqGGOaakcqWGYbGCdaWgaaWcbaGaemyzaugakeqaaiabgkHiTiabdkhaYnaaBaaaleaacqWGVbWBcqWGLbqzaOqabaGaeiykaKcabaGaemOCai3aaSbaaSqaaiabdsha0bGcbeaaaaGaeyypa0ZaaSGbaeaacqWF1oqzdaGadaqaaiab=n7aNjabcIcaOiabdkfasnaaBaaaleaacqWGNbWzcqWGLbqzaOqabaGaeyOeI0IaemOuai1aaSbaaSqaaiabdEgaNjabd+gaVbGcbeaacqGGPaqkcqGHRaWkcqGGOaakcqaIXaqmcqGHsislcqWFZoWzcqGGPaqkcqGGOaakcqWGsbGudaWgaaWcbaGaem4Ba8MaemyzaugakeqaaiabgkHiTiabdkfasnaaBaaaleaacqWGVbWBcqWGVbWBaOqabaGaeiykaKcacaGL7bGaayzFaaaabaGaemOCai3aaSbaaSqaaiabdsha0bGcbeaaaaGaaCzcaiaaxMaadaqadaqaaiabigdaXiabigdaXaGaayjkaiaawMcaaaaa@6C7B@

If the trait is a disease, PAF^E^_e _is the proportion of cases that could be avoided if an environmental intervention (such as a lifestyle change or reduction in exposure) succeeds in moving everyone in the 'high environmental risk group' to the 'low environmental risk' category, as shown in Figure 1b.

The targeted population attributable fraction (PAF^E^_ge_) may be defined as the proportion of cases that could be avoided by targeting the same environmental intervention at the 'high genotypic + high environmental risk' subgroup only (the 'ge' subgroup), as shown in Figure 1c. Again assuming no confounders, it is given by:

PAFgeE=εγ(Rge−Rgo)/rt     (12)
 MathType@MTEF@5@5@+=feaafiart1ev1aaatCvAUfKttLearuWrP9MDH5MBPbIqV92AaeXatLxBI9gBaebbnrfifHhDYfgasaacH8akY=wiFfYdH8Gipec8Eeeu0xXdbba9frFj0=OqFfea0dXdd9vqai=hGuQ8kuc9pgc9s8qqaq=dirpe0xb9q8qiLsFr0=vr0=vr0dc8meaabaqaciaacaGaaeqabaqabeGadaaakeaacqWGqbaucqWGbbqqcqWGgbGrdaqhaaWcbaGaem4zaCMaemyzaugabaGaemyraueaaOGaeyypa0dcciGae8xTduMae83SdCMaeiikaGIaemOuai1aaSbaaSqaaiabdEgaNjabdwgaLbGcbeaacqGHsislcqWGsbGudaWgaaWcbaGaem4zaCMaem4Ba8gakeqaaiabcMcaPiabc+caViabdkhaYnaaBaaaleaacqWG0baDaOqabaGaaCzcaiaaxMaadaqadaqaaiabigdaXiabikdaYaGaayjkaiaawMcaaaaa@4BB5@

Note that PAF^E^_ge _differs from PAF_ge _as defined by Khoury & Wagener [19]. The latter implicitly assumes that both environmental and genetic risk factors are reduced and thus is inappropriate for assessing the merits of a targeted environmental intervention. PAF^E^_ge _as defined here is instead equivalent to the targeted attributable fraction (AF_T_) defined by Khoury et al. [10]. To avoid confusion, the notation adopted here specifies both the nature of the intervention (environmental, denoted by superscript E) and the target subpopulation (the 'ge' subgroup, at both high genotypic and high environmental risk). Thus, the proportion of cases that would be avoided were it possible to move the 'high genotypic risk' subgroup to 'low genotypic risk' (as shown in Figure 1a) is written as PAF^G^_g_, given by:

PAFgG=γ(rg−rog)rt=γ{ε(Rge−Roe)+(1−ε)(Rgo−Roo)}/rt     (13)
 MathType@MTEF@5@5@+=feaafiart1ev1aaatCvAUfKttLearuWrP9MDH5MBPbIqV92AaeXatLxBI9gBaebbnrfifHhDYfgasaacH8akY=wiFfYdH8Gipec8Eeeu0xXdbba9frFj0=OqFfea0dXdd9vqai=hGuQ8kuc9pgc9s8qqaq=dirpe0xb9q8qiLsFr0=vr0=vr0dc8meaabaqaciaacaGaaeqabaqabeGadaaakeaacqWGqbaucqWGbbqqcqWGgbGrlmaaDaaabaGaem4zaCgabaGaem4raCeaaOGaeyypa0ZaaSaaaeaaiiGacqWFZoWzcqGGOaakcqWGYbGCdaWgaaWcbaGaem4zaCgakeqaaiabgkHiTiabdkhaYnaaBaaaleaacqWGVbWBcqWGNbWzaOqabaGaeiykaKcabaGaemOCai3cdaWgaaqaaiabdsha0bqabaaaaOWaaSGbaeaacqGH9aqpcqWFZoWzdaGadaqaaiab=v7aLjabcIcaOiabdkfasnaaBaaaleaacqWGNbWzcqWGLbqzaOqabaGaeyOeI0IaemOuai1cdaWgaaqaaiabd+gaVjabdwgaLbqabaGccqGGPaqkcqGHRaWkcqGGOaakcqaIXaqmcqGHsislcqWF1oqzcqGGPaqkcqGGOaakcqWGsbGudaWgaaWcbaGaem4zaCMaem4Ba8gakeqaaiabgkHiTiabdkfasnaaBaaaleaacqWGVbWBcqWGVbWBaOqabaGaeiykaKcacaGL7bGaayzFaaaabaGaemOCai3cdaWgaaqaaiabdsha0bqabaaaaOGaaCzcaiaaxMaadaqadaqaaiabigdaXiabiodaZaGaayjkaiaawMcaaaaa@6C8F@

Although in practice it is not possible to change the genotype of the population, the parameter PAF^G^_g _is nevertheless useful in the calculations that follow.

### Measures of utility

Khoury et al. [10] define the Population Impact (PI) as:

PI=PAFgeE/PAFeE     (14)
 MathType@MTEF@5@5@+=feaafiart1ev1aaatCvAUfKttLearuWrP9MDH5MBPbIqV92AaeXatLxBI9gBaebbnrfifHhDYfgasaacH8akY=wiFfYdH8Gipec8Eeeu0xXdbba9frFj0=OqFfea0dXdd9vqai=hGuQ8kuc9pgc9s8qqaq=dirpe0xb9q8qiLsFr0=vr0=vr0dc8meaabaqaciaacaGaaeqabaqabeGadaaakeaacqWGqbaucqWGjbqscqGH9aqpdaWcgaqaaiabdcfaqjabdgeabjabdAeagnaaDaaaleaacqWGNbWzcqWGLbqzaeaacqWGfbqraaaakeaacqWGqbaucqWGbbqqcqWGgbGrlmaaDaaabaGaemyzaugabaGaemyraueaaaaakiaaxMaacaWLjaWaaeWaaeaacqaIXaqmcqaI0aanaiaawIcacaGLPaaaaaa@41E2@

PI is one possible measure of the usefulness of targeting the environmental intervention (E) at the 'ge' subgroup. It measures the proportion of cases avoided by targeting the 'high genotypic + high environmental risk' subgroup (the 'ge' subgroup), compared to the proportion avoided by applying the environmental intervention to the whole 'high environmental risk' group. PI has the property:

0 ≤ *PI *≤ 1     (15)

and has its maximum value when PAF^E^_ge _= PAF^E^_e_. However, as a measure of the utility of genotyping, PI has the disadvantage that it takes no account of the proportion of the population γ in the high genotypic risk group. This means PI = 1 when γ = 1 simply because the whole population is then in the high genotypic risk group, although using genotyping to target environmental interventions is more likely to be useful if PI = 1 and γ is also small.

Therefore, consider an alternative utility parameter U_ge_, defined by:

Uge=PAFgeEPAFeE−γ=γ(1−γ)[(Rge−Rgo)−(Roe−Roo)][γ(Rge−Rgo)+(1−γ)(Roe−Roo)]     (16)
 MathType@MTEF@5@5@+=feaafiart1ev1aaatCvAUfKttLearuWrP9MDH5MBPbIqV92AaeXatLxBI9gBaebbnrfifHhDYfgasaacH8akY=wiFfYdH8Gipec8Eeeu0xXdbba9frFj0=OqFfea0dXdd9vqai=hGuQ8kuc9pgc9s8qqaq=dirpe0xb9q8qiLsFr0=vr0=vr0dc8meaabaqaciaacaGaaeqabaqabeGadaaakeaacqWGvbqvlmaaBaaabaGaem4zaCMaemyzaugabeaakiabg2da9maalaaabaGaemiuaaLaemyqaeKaemOray0cdaqhaaqaaiabdEgaNjabdwgaLbqaaiabdweafbaaaOqaaiabdcfaqjabdgeabjabdAeagTWaa0baaeaacqWGLbqzaeaacqWGfbqraaaaaOGaeyOeI0ccciGae83SdCMaeyypa0ZaaSaaaeaacqWFZoWzcqGGOaakcqaIXaqmcqGHsislcqWFZoWzcqGGPaqkdaWadaqaaiabcIcaOiabdkfasnaaBaaaleaacqWGNbWzcqWGLbqzaOqabaGaeyOeI0IaemOuai1aaSbaaSqaaiabdEgaNjabd+gaVbGcbeaacqGGPaqkcqGHsislcqGGOaakcqWGsbGudaWgaaWcbaGaem4Ba8MaemyzaugakeqaaiabgkHiTiabdkfasnaaBaaaleaacqWGVbWBcqWGVbWBaOqabaGaeiykaKcacaGLBbGaayzxaaaabaWaamWaaeaacqWFZoWzcqGGOaakcqWGsbGudaWgaaWcbaGaem4zaCMaemyzaugakeqaaiabgkHiTiabdkfasnaaBaaaleaacqWGNbWzcqWGVbWBaOqabaGaeiykaKIaey4kaSIaeiikaGIaeGymaeJaeyOeI0Iae83SdCMaeiykaKIaeiikaGIaemOuai1cdaWgaaqaaiabd+gaVjabdwgaLbqabaGccqGHsislcqWGsbGulmaaBaaabaGaem4Ba8Maem4Ba8gabeaakiabcMcaPaGaay5waiaaw2faaaaacaWLjaGaaCzcamaabmaabaGaeGymaeJaeGOnaydacaGLOaGaayzkaaaaaa@862D@

which has the property

-*γ *≤ *U*_*ge *_≤ (1-*γ*)     (17)

U_ge _tends to 1 only if PI = 1 and γ is also small. It is a measure of the utility of using genotyping to target the environmental intervention at the 'ge' subgroup, compared to randomly selecting the same proportion γ of the population to receive the intervention. U_ge _is positive if those at high genotypic risk have *more to gain *than those at low genotypic risk from the intervention ((R_ge_-R_go_) ≥ (R_oe_-R_oo_)) and negative if they have *less to gain *from the intervention. This reflects the fact that targeting those who have least to gain through an intervention is worse than using random selection in terms of its impact on population health.

Note that even if genotyping is better than random selection, other types of test that are more useful may be available [22]; a population-based approach still has the potential to reduce more cases of disease [9,19,23]; and such targeting also has broader psychological and social implications. Therefore a positive U_ge _does not necessarily imply that genotyping is the best means of selecting a subpopulation to target, or that a targeted approach is necessarily effective or socially acceptable. Note also that the measure U_ge _applies only to interventions that are considered applicable to the whole population (such as smoking cessation) and neglects other relevant issues such as cost-effectiveness and the burden of disease [24]. In addition, it is necessary to consider the magnitude of the Population Attributable Fraction, PAF^E^_e _before proposing this approach. This is because both PI and U_ge _may tend to unity even if only a small proportion of cases can be avoided by means of environmental interventions.

### Limits on parameters

Consider only populations where r_g _≥ r_og _and r_e _≥ r_oe _for all values of ε and γ. Then the risks in the four box model must be ordered such that:

1 ≥ *R*_*ge *_≥ *R*_*oe *_≥ *R*_*oo *_≥ 0     (18)

and

*R*_*ge *_≥ *R*_*go *_≥ *R*_*oo *_    (19)

Using the known relationships (Equations (11), (13) and (16)) between PAF^E^_e_, PAF^G^_g_, U_ge _and the risks R_oo_, R_go_, R_oe _and R_ge_, leads to the limits on the utility parameter U_ge _shown in Table 2. These conditions also ensure that PAF^E^_e_, PAF^G^_g _and PAF^E^_ge _are all positive. The two remaining inequalities (R_ge _≤ 1 and R_oo _≥ 0) are considered later, where they are used to derive limits on the proportion of the population in the 'high genotypic risk' group, γ. This step is not possible at this stage because PAF^E^_e_, PAF^G^_g _and PAF^E^_ge _are themselves dependent on γ.

**Table 2:** Constraints on model parameters

**Condition**	**Limits on U**_**ge**_	**Limits on γ**	**Limits on p**^**DZ**^_**g**_	**Limits on f**_**ge**_
*R*_*oe *_≥ *R*_*oo*_	*U*_*ge *_≤ (1 - *γ*)	*γ *≤ *γ*_max *ge *_where γmax⁡ge=11+VgeVe MathType@MTEF@5@5@+=feaafiart1ev1aaatCvAUfKttLearuWrP9MDH5MBPbIqV92AaeXatLxBI9gBaebbnrfifHhDYfgasaacH8akY=wiFfYdH8Gipec8Eeeu0xXdbba9frFj0=OqFfea0dXdd9vqai=hGuQ8kuc9pgc9s8qqaq=dirpe0xb9q8qiLsFr0=vr0=vr0dc8meaabaqaciaacaGaaeqabaqabeGadaaakeaaiiGacqWFZoWzdaWgaaWcbaGagiyBa0MaeiyyaeMaeiiEaGNaem4zaCMaemyzaugabeaakiabg2da9maalaaabaGaeGymaedabaGaeGymaeJaey4kaSYaaSaaaeaacqWGwbGvdaWgaaWcbaGaem4zaCMaemyzaugabeaaaOqaaiabdAfawnaaBaaaleaacqWGLbqzaeqaaaaaaaaaaa@4011@		

*R*_*go *_≥ *R*_*oo*_	Uge≤(1−γ)PAFgGPAFeE MathType@MTEF@5@5@+=feaafiart1ev1aaatCvAUfKttLearuWrP9MDH5MBPbIqV92AaeXatLxBI9gBaebbnrfifHhDYfgasaacH8akY=wiFfYdH8Gipec8Eeeu0xXdbba9frFj0=OqFfea0dXdd9vqai=hGuQ8kuc9pgc9s8qqaq=dirpe0xb9q8qiLsFr0=vr0=vr0dc8meaabaqaciaacaGaaeqabaqabeGadaaakeaacqWGvbqvdaWgaaWcbaGaem4zaCMaemyzaugabeaakiabgsMiJkabcIcaOiabigdaXiabgkHiTGGaciab=n7aNjabcMcaPmaalaaabaGaemiuaaLaemyqaeKaemOray0aa0baaSqaaiabdEgaNbqaaiabdEeahbaaaOqaaiabdcfaqjabdgeabjabdAeagnaaDaaaleaacqWGLbqzaeaacqWGfbqraaaaaaaa@438B@		pgDZ≤pgmax⁡DZ MathType@MTEF@5@5@+=feaafiart1ev1aaatCvAUfKttLearuWrP9MDH5MBPbIqV92AaeXatLxBI9gBaebbnrfifHhDYfgasaacH8akY=wiFfYdH8Gipec8Eeeu0xXdbba9frFj0=OqFfea0dXdd9vqai=hGuQ8kuc9pgc9s8qqaq=dirpe0xb9q8qiLsFr0=vr0=vr0dc8meaabaqaciaacaGaaeqabaqabeGadaaakeaacqWGWbaCdaqhaaWcbaGaem4zaCgabaGaemiraqKaemOwaOfaaOGaeyizImQaemiCaa3aa0baaSqaaiabdEgaNjGbc2gaTjabcggaHjabcIha4bqaaiabdseaejabdQfaAbaaaaa@3D07@	fge≤1PAFeE MathType@MTEF@5@5@+=feaafiart1ev1aaatCvAUfKttLearuWrP9MDH5MBPbIqV92AaeXatLxBI9gBaebbnrfifHhDYfgasaacH8akY=wiFfYdH8Gipec8Eeeu0xXdbba9frFj0=OqFfea0dXdd9vqai=hGuQ8kuc9pgc9s8qqaq=dirpe0xb9q8qiLsFr0=vr0=vr0dc8meaabaqaciaacaGaaeqabaqabeGadaaakeaacqWGMbGzdaWgaaWcbaGaem4zaCMaemyzaugabeaakiabgsMiJoaalaaabaGaeGymaedabaGaemiuaaLaemyqaeKaemOray0aa0baaSqaaiabdwgaLbqaaiabdweafbaaaaaaaa@3972@

*R*_*ge *_≥ *R*_*go*_	*U*_*ge *_≥ -*γ*	*γ *≥ *γ*_*neg *_where γneg=11+VeVge MathType@MTEF@5@5@+=feaafiart1ev1aaatCvAUfKttLearuWrP9MDH5MBPbIqV92AaeXatLxBI9gBaebbnrfifHhDYfgasaacH8akY=wiFfYdH8Gipec8Eeeu0xXdbba9frFj0=OqFfea0dXdd9vqai=hGuQ8kuc9pgc9s8qqaq=dirpe0xb9q8qiLsFr0=vr0=vr0dc8meaabaqaciaacaGaaeqabaqabeGadaaakeaaiiGacqWFZoWzdaWgaaWcbaGaemOBa4MaemyzauMaem4zaCgabeaakiabg2da9maalaaabaGaeGymaedabaGaeGymaeJaey4kaSYaaSaaaeaacqWGwbGvdaWgaaWcbaGaemyzaugabeaaaOqaaiabdAfawnaaBaaaleaacqWGNbWzcqWGLbqzaeqaaaaaaaaaaa@3D50@		

*R*_*ge *_≥ *R*_*oe*_	Uge≥−(1−γ)εPAFgG(1−ε)PAFeE MathType@MTEF@5@5@+=feaafiart1ev1aaatCvAUfKttLearuWrP9MDH5MBPbIqV92AaeXatLxBI9gBaebbnrfifHhDYfgasaacH8akY=wiFfYdH8Gipec8Eeeu0xXdbba9frFj0=OqFfea0dXdd9vqai=hGuQ8kuc9pgc9s8qqaq=dirpe0xb9q8qiLsFr0=vr0=vr0dc8meaabaqaciaacaGaaeqabaqabeGadaaakeaacqWGvbqvdaWgaaWcbaGaem4zaCMaemyzaugabeaakiabgwMiZkabgkHiTiabcIcaOiabigdaXiabgkHiTGGaciab=n7aNjabcMcaPmaalaaabaGae8xTduMaemiuaaLaemyqaeKaemOray0aa0baaSqaaiabdEgaNbqaaiabdEeahbaaaOqaaiabcIcaOiabigdaXiabgkHiTiab=v7aLjabcMcaPiabdcfaqjabdgeabjabdAeagnaaDaaaleaacqWGLbqzaeaacqWGfbqraaaaaaaa@4B5C@		pgDZ≤pgnegDZ MathType@MTEF@5@5@+=feaafiart1ev1aaatCvAUfKttLearuWrP9MDH5MBPbIqV92AaeXatLxBI9gBaebbnrfifHhDYfgasaacH8akY=wiFfYdH8Gipec8Eeeu0xXdbba9frFj0=OqFfea0dXdd9vqai=hGuQ8kuc9pgc9s8qqaq=dirpe0xb9q8qiLsFr0=vr0=vr0dc8meaabaqaciaacaGaaeqabaqabeGadaaakeaacqWGWbaCdaqhaaWcbaGaem4zaCgabaGaemiraqKaemOwaOfaaOGaeyizImQaemiCaa3aa0baaSqaaiabdEgaNjabd6gaUjabdwgaLjabdEgaNbqaaiabdseaejabdQfaAbaaaaa@3CF0@	fge≥−ε(1−ε)PAFeE MathType@MTEF@5@5@+=feaafiart1ev1aaatCvAUfKttLearuWrP9MDH5MBPbIqV92AaeXatLxBI9gBaebbnrfifHhDYfgasaacH8akY=wiFfYdH8Gipec8Eeeu0xXdbba9frFj0=OqFfea0dXdd9vqai=hGuQ8kuc9pgc9s8qqaq=dirpe0xb9q8qiLsFr0=vr0=vr0dc8meaabaqaciaacaGaaeqabaqabeGadaaakeaacqWGMbGzdaWgaaWcbaGaem4zaCMaemyzaugabeaakiabgwMiZkabgkHiTmaalaaabaacciGae8xTdugabaGaeiikaGIaeGymaeJaeyOeI0Iae8xTduMaeiykaKIaemiuaaLaemyqaeKaemOray0aa0baaSqaaiabdwgaLbqaaiabdweafbaaaaaaaa@405F@

*R*_*ge *_≤ 1		*γ *≥ *γ*_min *ge *_where γmin⁡ge=11+F12(Vg/rt2) MathType@MTEF@5@5@+=feaafiart1ev1aaatCvAUfKttLearuWrP9MDH5MBPbIqV92AaeXatLxBI9gBaebbnrfifHhDYfgasaacH8akY=wiFfYdH8Gipec8Eeeu0xXdbba9frFj0=OqFfea0dXdd9vqai=hGuQ8kuc9pgc9s8qqaq=dirpe0xb9q8qiLsFr0=vr0=vr0dc8meaabaqaciaacaGaaeqabaqabeGadaaakeaaiiGacqWFZoWzdaWgaaWcbaGagiyBa0MaeiyAaKMaeiOBa4Maem4zaCMaemyzaugabeaakiabg2da9maalaaabaGaeGymaedabaGaeGymaeJaey4kaSYaaSaaaeaacqWGgbGrdaqhaaWcbaGaeGymaedabaGaeGOmaidaaaGcbaWaaSGbaeaacqGGOaakcqWGwbGvdaWgaaWcbaGaem4zaCgabeaaaOqaaiabdkhaYnaaDaaaleaacqWG0baDaeaacqaIYaGmaaGccqGGPaqkaaaaaaaaaaa@4503@		

*R*_*oo *_≥ 0		*γ *≤ *γ*_*o *_where γo=11+F22(Vg/rt2) MathType@MTEF@5@5@+=feaafiart1ev1aaatCvAUfKttLearuWrP9MDH5MBPbIqV92AaeXatLxBI9gBaebbnrfifHhDYfgasaacH8akY=wiFfYdH8Gipec8Eeeu0xXdbba9frFj0=OqFfea0dXdd9vqai=hGuQ8kuc9pgc9s8qqaq=dirpe0xb9q8qiLsFr0=vr0=vr0dc8meaabaqaciaacaGaaeqabaqabeGadaaakeaaiiGacqWFZoWzdaWgaaWcbaGaem4Ba8gabeaakiabg2da9maalaaabaGaeGymaedabaGaeGymaeJaey4kaSIaemOray0aa0baaSqaaiabikdaYaqaaiabikdaYaaakiabcIcaOmaalyaabaGaemOvay1aaSbaaSqaaiabdEgaNbqabaaakeaacqWGYbGCdaqhaaWcbaGaemiDaqhabaGaeGOmaidaaaaakiabcMcaPaaaaaa@3F90@		

### The twin and familial risks model

Data from studies of monozygotic and dizygotic twins are commonly used to estimate the genetic and environmental variances V_g _and V_e _of a trait. Here, the aim is to use twin and other data to estimate the possible magnitudes of the population attributable fractions and measures of utility defined above. To do this it is necessary to estimate V_g_, V_e _and the variance due to gene-environment interaction, V_ge_. The standard methodology for twin data analysis is inappropriate because it assumes V_ge _= 0.

First note that we are interested in the extent to which relatives share *risk categories *(which may be either environmental or genotypic, or both), rather than a particular genetic variant. The probability that a relative of a proband is also a case depends on the extent to which their environmental and genotypic risks are correlated with those of the proband. Rather than adopting a specific form for the genetic model, define p^rel^_g _as the correlation in genotypic risk category (g) between relatives of type denoted by the superscript 'rel'. The parameter p^rel^_g _is the probability that the genotypic risk category (high or low) is identical by descent.

For monozygotic (MZ) twins, assumed to share their entire genome, p^MZ^_g _= 1. For dizygotic (DZ) twins and other siblings, who share half their genome, p^DZ^_g _= p^sib^_g _= 1/2 for a single allele model (dominant Mendelian disorder) or an additive polygenic model. For a two allele model (recessive Mendelian disorder) or the dominance term of a polygenic model (in which multiple pairs of alleles interact), p^DZ^_g _= p^sib^_g _= 1/4. Here, allowing for the possibility of multiple gene-gene interactions (epistasis), require only that:

1/2≥pgDZ≥0     (20)
 MathType@MTEF@5@5@+=feaafiart1ev1aaatCvAUfKttLearuWrP9MDH5MBPbIqV92AaeXatLxBI9gBaebbnrfifHhDYfgasaacH8akY=wiFfYdH8Gipec8Eeeu0xXdbba9frFj0=OqFfea0dXdd9vqai=hGuQ8kuc9pgc9s8qqaq=dirpe0xb9q8qiLsFr0=vr0=vr0dc8meaabaqaciaacaGaaeqabaqabeGadaaakeaadaWcgaqaaiabigdaXaqaaiabikdaYiabgwMiZkabdchaWnaaDaaaleaacqWGNbWzaeaacqWGebarcqWGAbGwaaaaaOGaeyyzImRaeGimaaJaaCzcaiaaxMaadaqadaqaaiabikdaYiabicdaWaGaayjkaiaawMcaaaaa@3D10@

The meaning of p^DZ^_g _and its relationship to the polygenic risk model first adopted by Ronald Fisher in 1918 is discussed further below.

Similarly, define p^rel^_e _as the correlation in environmental risk category (e) between relatives of type "rel", requiring only that:

1≥perel≥0     (21)
 MathType@MTEF@5@5@+=feaafiart1ev1aaatCvAUfKttLearuWrP9MDH5MBPbIqV92AaeXatLxBI9gBaebbnrfifHhDYfgasaacH8akY=wiFfYdH8Gipec8Eeeu0xXdbba9frFj0=OqFfea0dXdd9vqai=hGuQ8kuc9pgc9s8qqaq=dirpe0xb9q8qiLsFr0=vr0=vr0dc8meaabaqaciaacaGaaeqabaqabeGadaaakeaacqaIXaqmcqGHLjYScqWGWbaCdaqhaaWcbaGaemyzaugabaGaemOCaiNaemyzauMaemiBaWgaaOGaeyyzImRaeGimaaJaaCzcaiaaxMaadaqadaqaaiabikdaYiabigdaXaGaayjkaiaawMcaaaaa@3DD9@

Assume that p^rel^_g _and p^rel^_e _are independent (so that there is no genotype-environment correlation) and that risks within a category are randomly distributed. The relative risk for a relative of type "rel" may then be written:

λrel=(1−pgrel)(1−perel)+pgrel(1−perel)RRgencases+(1−pgrel)perelRRenvcases+pgrelperelRRcases     (22)
 MathType@MTEF@5@5@+=feaafiart1ev1aaatCvAUfKttLearuWrP9MDH5MBPbIqV92AaeXatLxBI9gBaebbnrfifHhDYfgasaacH8akY=wiFfYdH8Gipec8Eeeu0xXdbba9frFj0=OqFfea0dXdd9vqai=hGuQ8kuc9pgc9s8qqaq=dirpe0xb9q8qiLsFr0=vr0=vr0dc8meaabaqaciaacaGaaeqabaqabeGadaaakeaaiiGacqWF7oaBdaWgaaWcbaGaemOCaiNaemyzauMaemiBaWgabeaakiabg2da9iabcIcaOiabigdaXiabgkHiTiabdchaWnaaDaaaleaacqWGNbWzaeaacqWGYbGCcqWGLbqzcqWGSbaBaaGccqGGPaqkcqGGOaakcqaIXaqmcqGHsislcqWGWbaCdaqhaaWcbaGaemyzaugabaGaemOCaiNaemyzauMaemiBaWgaaOGaeiykaKIaey4kaSIaemiCaa3aa0baaSqaaiabdEgaNbqaaiabdkhaYjabdwgaLjabdYgaSbaakiabcIcaOiabigdaXiabgkHiTiabdchaWnaaDaaaleaacqWGLbqzaeaacqWGYbGCcqWGLbqzcqWGSbaBaaGccqGGPaqkcqWGsbGucqWGsbGudaqhaaWcbaGaem4zaCMaemyzauMaemOBa4gabaGaem4yamMaemyyaeMaem4CamNaemyzauMaem4CamhaaOGaey4kaSIaeiikaGIaeGymaeJaeyOeI0IaemiCaa3aa0baaSqaaiabdEgaNbqaaiabdkhaYjabdwgaLjabdYgaSbaakiabcMcaPiabdchaWnaaDaaaleaacqWGLbqzaeaacqWGYbGCcqWGLbqzcqWGSbaBaaGccqWGsbGucqWGsbGudaqhaaWcbaGaemyzauMaemOBa4MaemODayhabaGaem4yamMaemyyaeMaem4CamNaemyzauMaem4CamhaaOGaey4kaSIaemiCaa3aa0baaSqaaiabdEgaNbqaaiabdkhaYjabdwgaLjabdYgaSbaakiabdchaWnaaDaaaleaacqWGLbqzaeaacqWGYbGCcqWGLbqzcqWGSbaBaaGccqWGsbGucqWGsbGudaahaaWcbeqaaiabdogaJjabdggaHjabdohaZjabdwgaLjabdohaZbaakiaaxMaacaWLjaWaaeWaaeaacqaIYaGmcqaIYaGmaiaawIcacaGLPaaaaaa@A657@

Substituting for the relative risks RR^cases^_gen_, RR^cases^_env _and RR^cases ^using Equations (8), (9) and (10) leads (after some algebra) to:

λrel−1=pgrelVgrt2+perelVert2+pgrelperelVgert2     (23)
 MathType@MTEF@5@5@+=feaafiart1ev1aaatCvAUfKttLearuWrP9MDH5MBPbIqV92AaeXatLxBI9gBaebbnrfifHhDYfgasaacH8akY=wiFfYdH8Gipec8Eeeu0xXdbba9frFj0=OqFfea0dXdd9vqai=hGuQ8kuc9pgc9s8qqaq=dirpe0xb9q8qiLsFr0=vr0=vr0dc8meaabaqaciaacaGaaeqabaqabeGadaaakeaaiiGacqWF7oaBdaWgaaWcbaGaemOCaiNaemyzauMaemiBaWgabeaakiabgkHiTiabigdaXiabg2da9iabdchaWnaaDaaaleaacqWGNbWzaeaacqWGYbGCcqWGLbqzcqWGSbaBaaGcdaWcaaqaaiabdAfawnaaBaaaleaacqWGNbWzaeqaaaGcbaGaemOCai3aa0baaSqaaiabdsha0bqaaiabikdaYaaaaaGccqGHRaWkcqWGWbaCdaqhaaWcbaGaemyzaugabaGaemOCaiNaemyzauMaemiBaWgaaOWaaSaaaeaacqWGwbGvdaWgaaWcbaGaemyzaugabeaaaOqaaiabdkhaYnaaDaaaleaacqWG0baDaeaacqaIYaGmaaaaaOGaey4kaSIaemiCaa3aa0baaSqaaiabdEgaNbqaaiabdkhaYjabdwgaLjabdYgaSbaakiabdchaWnaaDaaaleaacqWGLbqzaeaacqWGYbGCcqWGLbqzcqWGSbaBaaGcdaWcaaqaaiabdAfawnaaBaaaleaacqWGNbWzcqWGLbqzaeqaaaGcbaGaemOCai3aa0baaSqaaiabdsha0bqaaiabikdaYaaaaaGccaWLjaGaaCzcamaabmaabaGaeGOmaiJaeG4mamdacaGLOaGaayzkaaaaaa@6E4A@

where

Vert2=(1−ε)ε[PAFeE]2     (24)
 MathType@MTEF@5@5@+=feaafiart1ev1aaatCvAUfKttLearuWrP9MDH5MBPbIqV92AaeXatLxBI9gBaebbnrfifHhDYfgasaacH8akY=wiFfYdH8Gipec8Eeeu0xXdbba9frFj0=OqFfea0dXdd9vqai=hGuQ8kuc9pgc9s8qqaq=dirpe0xb9q8qiLsFr0=vr0=vr0dc8meaabaqaciaacaGaaeqabaqabeGadaaakeaadaWcaaqaaiabdAfawnaaBaaaleaacqWGLbqzaeqaaaGcbaGaemOCai3aa0baaSqaaiabdsha0bqaaiabikdaYaaaaaGccqGH9aqpdaWcaaqaaiabcIcaOiabigdaXiabgkHiTGGaciab=v7aLjabcMcaPaqaaiab=v7aLbaadaWadaqaaiabdcfaqjabdgeabjabdAeagnaaDaaaleaacqWGLbqzaeaacqWGfbqraaaakiaawUfacaGLDbaadaahaaWcbeqaaiabikdaYaaakiaaxMaacaWLjaWaaeWaaeaacqaIYaGmcqaI0aanaiaawIcacaGLPaaaaaa@492C@

Vgrt2=(1−γ)γ[PAFgG]2     (25)
 MathType@MTEF@5@5@+=feaafiart1ev1aaatCvAUfKttLearuWrP9MDH5MBPbIqV92AaeXatLxBI9gBaebbnrfifHhDYfgasaacH8akY=wiFfYdH8Gipec8Eeeu0xXdbba9frFj0=OqFfea0dXdd9vqai=hGuQ8kuc9pgc9s8qqaq=dirpe0xb9q8qiLsFr0=vr0=vr0dc8meaabaqaciaacaGaaeqabaqabeGadaaakeaadaWcaaqaaiabdAfawnaaBaaaleaacqWGNbWzaeqaaaGcbaGaemOCai3aa0baaSqaaiabdsha0bqaaiabikdaYaaaaaGccqGH9aqpdaWcaaqaaiabcIcaOiabigdaXiabgkHiTGGaciab=n7aNjabcMcaPaqaaiab=n7aNbaadaWadaqaaiabdcfaqjabdgeabjabdAeagnaaDaaaleaacqWGNbWzaeaacqWGhbWraaaakiaawUfacaGLDbaadaahaaWcbeqaaiabikdaYaaakiaaxMaacaWLjaWaaeWaaeaacqaIYaGmcqaI1aqnaiaawIcacaGLPaaaaaa@493A@

Vgert2=(1−ε)εγ(1−γ)[UgePAFeE]2     (26)
 MathType@MTEF@5@5@+=feaafiart1ev1aaatCvAUfKttLearuWrP9MDH5MBPbIqV92AaeXatLxBI9gBaebbnrfifHhDYfgasaacH8akY=wiFfYdH8Gipec8Eeeu0xXdbba9frFj0=OqFfea0dXdd9vqai=hGuQ8kuc9pgc9s8qqaq=dirpe0xb9q8qiLsFr0=vr0=vr0dc8meaabaqaciaacaGaaeqabaqabeGadaaakeaadaWcaaqaaiabdAfawnaaBaaaleaacqWGNbWzcqWGLbqzaeqaaaGcbaGaemOCai3aa0baaSqaaiabdsha0bqaaiabikdaYaaaaaGccqGH9aqpdaWcaaqaaiabcIcaOiabigdaXiabgkHiTGGaciab=v7aLjabcMcaPaqaaiab=v7aLjab=n7aNjabcIcaOiabigdaXiabgkHiTiab=n7aNjabcMcaPaaadaWadaqaaiabdwfavnaaBaaaleaacqWGNbWzcqWGLbqzaeqaaOGaemiuaaLaemyqaeKaemOray0aa0baaSqaaiabdwgaLbqaaiabdweafbaaaOGaay5waiaaw2faamaaCaaaleqabaGaeGOmaidaaOGaaCzcaiaaxMaadaqadaqaaiabikdaYiabiAda2aGaayjkaiaawMcaaaaa@556D@

Note that if the G-E interaction component of the variance, V_ge_, is zero, the utility of targeting the environmental intervention by genotype, U_ge_, is also zero (Equation (26)), because those at high genotypic risk have no more to gain from the intervention than those at low genotypic risk (R_ge_-R_go _= R_oe_-R_oo_).

Equation (23) can also be derived more formally using matrix methods (Appendix A).

### The gene-environment interaction factor and remaining inequalities

Without loss of generality, define the gene-environment interaction factor f_ge _such that:

Vgert2=fge2Vgrt2.Vert2     (27)
 MathType@MTEF@5@5@+=feaafiart1ev1aaatCvAUfKttLearuWrP9MDH5MBPbIqV92AaeXatLxBI9gBaebbnrfifHhDYfgasaacH8akY=wiFfYdH8Gipec8Eeeu0xXdbba9frFj0=OqFfea0dXdd9vqai=hGuQ8kuc9pgc9s8qqaq=dirpe0xb9q8qiLsFr0=vr0=vr0dc8meaabaqaciaacaGaaeqabaqabeGadaaakeaadaWcaaqaaiabdAfawnaaBaaaleaacqWGNbWzcqWGLbqzaeqaaaGcbaGaemOCai3aa0baaSqaaiabdsha0bqaaiabikdaYaaaaaGccqGH9aqpcqWGMbGzdaqhaaWcbaGaem4zaCMaemyzaugabaGaeGOmaidaaOWaaSaaaeaacqWGwbGvdaWgaaWcbaGaem4zaCgabeaaaOqaaiabdkhaYnaaDaaaleaacqWG0baDaeaacqaIYaGmaaaaaOGaeiOla4YaaSaaaeaacqWGwbGvdaWgaaWcbaGaemyzaugabeaaaOqaaiabdkhaYnaaDaaaleaacqWG0baDaeaacqaIYaGmaaaaaOGaaCzcaiaaxMaadaqadaqaaiabikdaYiabiEda3aGaayjkaiaawMcaaaaa@4E53@

and choose its sign so that (combining Equations (24), (25) and (26)):

Uge=fgeγ(1−γ)Vgrt2     (28)
 MathType@MTEF@5@5@+=feaafiart1ev1aaatCvAUfKttLearuWrP9MDH5MBPbIqV92AaeXatLxBI9gBaebbnrfifHhDYfgasaacH8akY=wiFfYdH8Gipec8Eeeu0xXdbba9frFj0=OqFfea0dXdd9vqai=hGuQ8kuc9pgc9s8qqaq=dirpe0xb9q8qiLsFr0=vr0=vr0dc8meaabaqaciaacaGaaeqabaqabeGadaaakeaacqWGvbqvdaWgaaWcbaGaem4zaCMaemyzaugabeaakiabg2da9iabdAgaMnaaBaaaleaacqWGNbWzcqWGLbqzaeqaaOWaaOaaaeaaiiGacqWFZoWzcqGGOaakcqaIXaqmcqGHsislcqWFZoWzcqGGPaqkdaWcaaqaaiabdAfawnaaBaaaleaacqWGNbWzaeqaaaGcbaGaemOCai3aa0baaSqaaiabdsha0bqaaiabikdaYaaaaaaabeaakiaaxMaacaWLjaWaaeWaaeaacqaIYaGmcqaI4aaoaiaawIcacaGLPaaaaaa@487F@

U_ge _is zero if f_ge _= 0 (i.e. for an additive G-E model, with no G-E interaction), but for a given γ and V_g_, U_ge _increases with increasing gene-environment interaction factor, f_ge_. For a fixed f_ge _and genetic variance component V_g_, U_ge _is maximum when γ = 1/2, i.e. when half the population is in the high genotypic risk group, provided solutions with γ = 1/2 exist (see also below: *cases where γ*_maxge_* < 1/2*).

Using the definitions of V_e_, V_g _and V_ge _(Equations (24), (25) and (26)) and the remaining inequalities, R_ge _≤ 1 and R_oo _≥ 0, two limits can be derived on the proportion of the population in the 'high genotypic risk' group, γ (see Table 2).

### Scoping studies

The general system of equations represented by Equation (23) may be simplified where data exist from monozygotic twins, dizygotic twins and other siblings, such that λ_DZ _> λ_sib_. This implies that environmental risks are more strongly correlated in dizygotic twins than in other siblings, p^e^_DZ _> p^e^_sib_. Remembering that p^MZ^_g _= 1 and p^sib^_g _= p^DZ^_g_, three independent equations for the relative risk in monozygotic, dizygotic twins and siblings may then be written:

λMZ−1=Vgrt2+peMZVert2+peMZVgert2     (29)
 MathType@MTEF@5@5@+=feaafiart1ev1aaatCvAUfKttLearuWrP9MDH5MBPbIqV92AaeXatLxBI9gBaebbnrfifHhDYfgasaacH8akY=wiFfYdH8Gipec8Eeeu0xXdbba9frFj0=OqFfea0dXdd9vqai=hGuQ8kuc9pgc9s8qqaq=dirpe0xb9q8qiLsFr0=vr0=vr0dc8meaabaqaciaacaGaaeqabaqabeGadaaakeaaiiGacqWF7oaBdaWgaaWcbaGaemyta0KaemOwaOfabeaakiabgkHiTiabigdaXiabg2da9maalaaabaGaemOvay1aaSbaaSqaaiabdEgaNbqabaaakeaacqWGYbGCdaqhaaWcbaGaemiDaqhabaGaeGOmaidaaaaakiabgUcaRiabdchaWnaaDaaaleaacqWGLbqzaeaacqWGnbqtcqWGAbGwaaGcdaWcaaqaaiabdAfawnaaBaaaleaacqWGLbqzaeqaaaGcbaGaemOCai3aa0baaSqaaiabdsha0bqaaiabikdaYaaaaaGccqGHRaWkcqWGWbaCdaqhaaWcbaGaemyzaugabaGaemyta0KaemOwaOfaaOWaaSaaaeaacqWGwbGvdaWgaaWcbaGaem4zaCMaemyzaugabeaaaOqaaiabdkhaYnaaDaaaleaacqWG0baDaeaacqaIYaGmaaaaaOGaaCzcaiaaxMaadaqadaqaaiabikdaYiabiMda5aGaayjkaiaawMcaaaaa@5AE3@

λDZ−1=pgDZVgrt2+peDZVert2+pgDZpeDZVgert2     (30)
 MathType@MTEF@5@5@+=feaafiart1ev1aaatCvAUfKttLearuWrP9MDH5MBPbIqV92AaeXatLxBI9gBaebbnrfifHhDYfgasaacH8akY=wiFfYdH8Gipec8Eeeu0xXdbba9frFj0=OqFfea0dXdd9vqai=hGuQ8kuc9pgc9s8qqaq=dirpe0xb9q8qiLsFr0=vr0=vr0dc8meaabaqaciaacaGaaeqabaqabeGadaaakeaaiiGacqWF7oaBdaWgaaWcbaGaemiraqKaemOwaOfabeaakiabgkHiTiabigdaXiabg2da9iabdchaWnaaDaaaleaacqWGNbWzaeaacqWGebarcqWGAbGwaaGcdaWcaaqaaiabdAfawnaaBaaaleaacqWGNbWzaeqaaaGcbaGaemOCai3aa0baaSqaaiabdsha0bqaaiabikdaYaaaaaGccqGHRaWkcqWGWbaCdaqhaaWcbaGaemyzaugabaGaemiraqKaemOwaOfaaOWaaSaaaeaacqWGwbGvdaWgaaWcbaGaemyzaugabeaaaOqaaiabdkhaYnaaDaaaleaacqWG0baDaeaacqaIYaGmaaaaaOGaey4kaSIaemiCaa3aa0baaSqaaiabdEgaNbqaaiabdseaejabdQfaAbaakiabdchaWnaaDaaaleaacqWGLbqzaeaacqWGebarcqWGAbGwaaGcdaWcaaqaaiabdAfawnaaBaaaleaacqWGNbWzcqWGLbqzaeqaaaGcbaGaemOCai3aa0baaSqaaiabdsha0bqaaiabikdaYaaaaaGccaWLjaGaaCzcamaabmaabaGaeG4mamJaeGimaadacaGLOaGaayzkaaaaaa@6527@

λsib−1=pgDZVgrt2+pesibVert2+pgDZpesibVgert2     (31)
 MathType@MTEF@5@5@+=feaafiart1ev1aaatCvAUfKttLearuWrP9MDH5MBPbIqV92AaeXatLxBI9gBaebbnrfifHhDYfgasaacH8akY=wiFfYdH8Gipec8Eeeu0xXdbba9frFj0=OqFfea0dXdd9vqai=hGuQ8kuc9pgc9s8qqaq=dirpe0xb9q8qiLsFr0=vr0=vr0dc8meaabaqaciaacaGaaeqabaqabeGadaaakeaaiiGacqWF7oaBdaWgaaWcbaGaem4CamNaemyAaKMaemOyaigabeaakiabgkHiTiabigdaXiabg2da9iabdchaWnaaDaaaleaacqWGNbWzaeaacqWGebarcqWGAbGwaaGcdaWcaaqaaiabdAfawnaaBaaaleaacqWGNbWzaeqaaaGcbaGaemOCai3aa0baaSqaaiabdsha0bqaaiabikdaYaaaaaGccqGHRaWkcqWGWbaCdaqhaaWcbaGaemyzaugabaGaem4CamNaemyAaKMaemOyaigaaOWaaSaaaeaacqWGwbGvdaWgaaWcbaGaemyzaugabeaaaOqaaiabdkhaYnaaDaaaleaacqWG0baDaeaacqaIYaGmaaaaaOGaey4kaSIaemiCaa3aa0baaSqaaiabdEgaNbqaaiabdseaejabdQfaAbaakiabdchaWnaaDaaaleaacqWGLbqzaeaacqWGZbWCcqWGPbqAcqWGIbGyaaGcdaWcaaqaaiabdAfawnaaBaaaleaacqWGNbWzcqWGLbqzaeqaaaGcbaGaemOCai3aa0baaSqaaiabdsha0bqaaiabikdaYaaaaaGccaWLjaGaaCzcamaabmaabaGaeG4mamJaeGymaedacaGLOaGaayzkaaaaaa@6A84@

To solve, assume the recurrence risks λ are known (see Appendix B and [25]) and define:

RMD=λMZ−1λDZ−1     (32)
 MathType@MTEF@5@5@+=feaafiart1ev1aaatCvAUfKttLearuWrP9MDH5MBPbIqV92AaeXatLxBI9gBaebbnrfifHhDYfgasaacH8akY=wiFfYdH8Gipec8Eeeu0xXdbba9frFj0=OqFfea0dXdd9vqai=hGuQ8kuc9pgc9s8qqaq=dirpe0xb9q8qiLsFr0=vr0=vr0dc8meaabaqaciaacaGaaeqabaqabeGadaaakeaacqWGsbGudaWgaaWcbaGaemyta0Kaemiraqeabeaakiabg2da9maalaaabaacciGae83UdW2aaSbaaSqaaiabd2eanjabdQfaAbqabaGccqGHsislcqaIXaqmaeaacqWF7oaBdaWgaaWcbaGaemiraqKaemOwaOfabeaakiabgkHiTiabigdaXaaacaWLjaGaaCzcamaabmaabaGaeG4mamJaeGOmaidacaGLOaGaayzkaaaaaa@424A@

RSD=λsib−1λDZ−1     (33)
 MathType@MTEF@5@5@+=feaafiart1ev1aaatCvAUfKttLearuWrP9MDH5MBPbIqV92AaeXatLxBI9gBaebbnrfifHhDYfgasaacH8akY=wiFfYdH8Gipec8Eeeu0xXdbba9frFj0=OqFfea0dXdd9vqai=hGuQ8kuc9pgc9s8qqaq=dirpe0xb9q8qiLsFr0=vr0=vr0dc8meaabaqaciaacaGaaeqabaqabeGadaaakeaacqWGsbGudaWgaaWcbaGaem4uamLaemiraqeabeaakiabg2da9maalaaabaacciGae83UdW2aaSbaaSqaaiabdohaZjabdMgaPjabdkgaIbqabaGccqGHsislcqaIXaqmaeaacqWF7oaBdaWgaaWcbaGaemiraqKaemOwaOfabeaakiabgkHiTiabigdaXaaacaWLjaGaaCzcamaabmaabaGaeG4mamJaeG4mamdacaGLOaGaayzkaaaaaa@440F@

with

*R*_*MD *_≥ 1     (34)

and

0 ≤ *R*_*SD *_≤ 1.     (35)

Note that if R_SD _= 1, Equations (30) and (31) are identical, p^e^_DZ _= p^e^_sib_, and more relatives are needed to obtain solutions, except in the special case where there is no environmental variance (see below: *no environmental variance*).

In addition, define the variable parameters (assumed unknown):

cMD=peMZpeDZ     (36)
 MathType@MTEF@5@5@+=feaafiart1ev1aaatCvAUfKttLearuWrP9MDH5MBPbIqV92AaeXatLxBI9gBaebbnrfifHhDYfgasaacH8akY=wiFfYdH8Gipec8Eeeu0xXdbba9frFj0=OqFfea0dXdd9vqai=hGuQ8kuc9pgc9s8qqaq=dirpe0xb9q8qiLsFr0=vr0=vr0dc8meaabaqaciaacaGaaeqabaqabeGadaaakeaacqWGJbWydaWgaaWcbaGaemyta0Kaemiraqeabeaakiabg2da9maalaaabaGaemiCaa3aa0baaSqaaiabdwgaLbqaaiabd2eanjabdQfaAbaaaOqaaiabdchaWnaaDaaaleaacqWGLbqzaeaacqWGebarcqWGAbGwaaaaaOGaaCzcaiaaxMaadaqadaqaaiabiodaZiabiAda2aGaayjkaiaawMcaaaaa@40CA@

cSD=pesibpeDZ     (37)
 MathType@MTEF@5@5@+=feaafiart1ev1aaatCvAUfKttLearuWrP9MDH5MBPbIqV92AaeXatLxBI9gBaebbnrfifHhDYfgasaacH8akY=wiFfYdH8Gipec8Eeeu0xXdbba9frFj0=OqFfea0dXdd9vqai=hGuQ8kuc9pgc9s8qqaq=dirpe0xb9q8qiLsFr0=vr0=vr0dc8meaabaqaciaacaGaaeqabaqabeGadaaakeaacqWGJbWydaWgaaWcbaGaem4uamLaemiraqeabeaakiabg2da9maalaaabaGaemiCaa3aa0baaSqaaiabdwgaLbqaaiabdohaZjabdMgaPjabdkgaIbaaaOqaaiabdchaWnaaDaaaleaacqWGLbqzaeaacqWGebarcqWGAbGwaaaaaOGaaCzcaiaaxMaadaqadaqaaiabiodaZiabiEda3aGaayjkaiaawMcaaaaa@428F@

with

*c*_*MD *_≥ 1     (38)

and

0 ≤ *c*_*SD *_≤ 1.     (39)

For λ_DZ _> 1 and R_SD _< 1 the simultaneous Equations (29), (30) and (31) can then be solved to give:

Vgrt2=(λDZ−1)pgDZ.(RSD−cSD)(1−cSD)     (40)
 MathType@MTEF@5@5@+=feaafiart1ev1aaatCvAUfKttLearuWrP9MDH5MBPbIqV92AaeXatLxBI9gBaebbnrfifHhDYfgasaacH8akY=wiFfYdH8Gipec8Eeeu0xXdbba9frFj0=OqFfea0dXdd9vqai=hGuQ8kuc9pgc9s8qqaq=dirpe0xb9q8qiLsFr0=vr0=vr0dc8meaabaqaciaacaGaaeqabaqabeGadaaakeaadaWcaaqaaiabdAfawnaaBaaaleaacqWGNbWzaeqaaaGcbaGaemOCai3aa0baaSqaaiabdsha0bqaaiabikdaYaaaaaGccqGH9aqpdaWcaaqaamaabmaabaacciGae83UdW2aaSbaaSqaaiabdseaejabdQfaAbqabaGccqGHsislcqaIXaqmaiaawIcacaGLPaaaaeaacqWGWbaCdaqhaaWcbaGaem4zaCgabaGaemiraqKaemOwaOfaaaaakiabc6caUmaalaaabaWaaeWaaeaacqWGsbGudaWgaaWcbaGaem4uamLaemiraqeabeaakiabgkHiTiabdogaJnaaBaaaleaacqWGtbWucqWGebaraeqaaaGccaGLOaGaayzkaaaabaWaaeWaaeaacqaIXaqmcqGHsislcqWGJbWydaWgaaWcbaGaem4uamLaemiraqeabeaaaOGaayjkaiaawMcaaaaacaWLjaGaaCzcamaabmaabaGaeGinaqJaeGimaadacaGLOaGaayzkaaaaaa@5833@

Vert2=(λDZ−1)peDZcMD(1−pgDZ)[(cMD−1)(1−RSD)(1−cSD)+(1−pgDZRMD)]     (41)
 MathType@MTEF@5@5@+=feaafiart1ev1aaatCvAUfKttLearuWrP9MDH5MBPbIqV92AaeXatLxBI9gBaebbnrfifHhDYfgasaacH8akY=wiFfYdH8Gipec8Eeeu0xXdbba9frFj0=OqFfea0dXdd9vqai=hGuQ8kuc9pgc9s8qqaq=dirpe0xb9q8qiLsFr0=vr0=vr0dc8meaabaqaciaacaGaaeqabaqabeGadaaakeaadaWcaaqaaiabdAfawnaaBaaaleaacqWGLbqzaeqaaaGcbaGaemOCai3aa0baaSqaaiabdsha0bqaaiabikdaYaaaaaGccqGH9aqpdaWcaaqaaiabcIcaOGGaciab=T7aSnaaBaaaleaacqWGebarcqWGAbGwaeqaaOGaeyOeI0IaeGymaeJaeiykaKcabaGaemiCaa3aa0baaSqaaiabdwgaLbqaaiabdseaejabdQfaAbaakiabdogaJnaaBaaaleaacqWGnbqtcqWGebaraeqaaOGaeiikaGIaeGymaeJaeyOeI0IaemiCaa3aa0baaSqaaiabdEgaNbqaaiabdseaejabdQfaAbaakiabcMcaPaaadaWadaqaamaalaaabaGaeiikaGIaem4yam2aaSbaaSqaaiabd2eanjabdseaebqabaGccqGHsislcqaIXaqmcqGGPaqkcqGGOaakcqaIXaqmcqGHsislcqWGsbGudaWgaaWcbaGaem4uamLaemiraqeabeaakiabcMcaPaqaaiabcIcaOiabigdaXiabgkHiTiabdogaJnaaBaaaleaacqWGtbWucqWGebaraeqaaOGaeiykaKcaaiabgUcaRiabcIcaOiabigdaXiabgkHiTiabdchaWnaaDaaaleaacqWGNbWzaeaacqWGebarcqWGAbGwaaGccqWGsbGudaWgaaWcbaGaemyta0KaemiraqeabeaakiabcMcaPaGaay5waiaaw2faaiaaxMaacaWLjaWaaeWaaeaacqaI0aancqaIXaqmaiaawIcacaGLPaaaaaa@7803@

Vgert2=(λDZ−1)peDZpgDZcMD(1−pgDZ)[(1−cMDpgDZ)(1−RSD)(1−cSD)+(1−pgDZRMD)]     (42)
 MathType@MTEF@5@5@+=feaafiart1ev1aaatCvAUfKttLearuWrP9MDH5MBPbIqV92AaeXatLxBI9gBaebbnrfifHhDYfgasaacH8akY=wiFfYdH8Gipec8Eeeu0xXdbba9frFj0=OqFfea0dXdd9vqai=hGuQ8kuc9pgc9s8qqaq=dirpe0xb9q8qiLsFr0=vr0=vr0dc8meaabaqaciaacaGaaeqabaqabeGadaaakeaadaWcaaqaaiabdAfawnaaBaaaleaacqWGNbWzcqWGLbqzaeqaaaGcbaGaemOCai3aa0baaSqaaiabdsha0bqaaiabikdaYaaaaaGccqGH9aqpdaWcaaqaaiabcIcaOGGaciab=T7aSnaaBaaaleaacqWGebarcqWGAbGwaeqaaOGaeyOeI0IaeGymaeJaeiykaKcabaGaemiCaa3aa0baaSqaaiabdwgaLbqaaiabdseaejabdQfaAbaakiabdchaWnaaDaaaleaacqWGNbWzaeaacqWGebarcqWGAbGwaaGccqWGJbWydaWgaaWcbaGaemyta0KaemiraqeabeaakiabcIcaOiabigdaXiabgkHiTiabdchaWnaaDaaaleaacqWGNbWzaeaacqWGebarcqWGAbGwaaGccqGGPaqkaaWaamWaaeaadaWcaaqaaiabcIcaOiabigdaXiabgkHiTiabdogaJnaaBaaaleaacqWGnbqtcqWGebaraeqaaOGaemiCaa3aa0baaSqaaiabdEgaNbqaaiabdseaejabdQfaAbaakiabcMcaPiabcIcaOiabigdaXiabgkHiTiabdkfasnaaBaaaleaacqWGtbWucqWGebaraeqaaOGaeiykaKcabaGaeiikaGIaeGymaeJaeyOeI0Iaem4yam2aaSbaaSqaaiabdofatjabdseaebqabaGccqGGPaqkaaGaey4kaSIaeiikaGIaeGymaeJaeyOeI0IaemiCaa3aa0baaSqaaiabdEgaNbqaaiabdseaejabdQfaAbaakiabdkfasnaaBaaaleaacqWGnbqtcqWGebaraeqaaOGaeiykaKcacaGLBbGaayzxaaGaaCzcaiaaxMaadaqadaqaaiabisda0iabikdaYaGaayjkaiaawMcaaaaa@83E6@

provided pgDZ
 MathType@MTEF@5@5@+=feaafiart1ev1aaatCvAUfKttLearuWrP9MDH5MBPbIqV92AaeXatLxBI9gBaebbnrfifHhDYfgasaacH8akY=wiFfYdH8Gipec8Eeeu0xXdbba9frFj0=OqFfea0dXdd9vqai=hGuQ8kuc9pgc9s8qqaq=dirpe0xb9q8qiLsFr0=vr0=vr0dc8meaabaqaciaacaGaaeqabaqabeGadaaakeaacqWGWbaCdaqhaaWcbaGaem4zaCgabaGaemiraqKaemOwaOfaaaaa@31E7@ ≠ 0, peDZ
 MathType@MTEF@5@5@+=feaafiart1ev1aaatCvAUfKttLearuWrP9MDH5MBPbIqV92AaeXatLxBI9gBaebbnrfifHhDYfgasaacH8akY=wiFfYdH8Gipec8Eeeu0xXdbba9frFj0=OqFfea0dXdd9vqai=hGuQ8kuc9pgc9s8qqaq=dirpe0xb9q8qiLsFr0=vr0=vr0dc8meaabaqaciaacaGaaeqabaqabeGadaaakeaacqWGWbaCdaqhaaWcbaGaemyzaugabaGaemiraqKaemOwaOfaaaaa@31E3@ ≠ 0 and *c*_*SD *_≠ 1 (see also below).

For situations in which a targeted intervention is under consideration, the population attributable fraction PAF^E^_e _and exposure ε are likely to be known, allowing V_e _to be treated as an input variable. However, p^DZ^_e _is usually unknown, since environmental correlations are often difficult to measure. Therefore, it is useful to eliminate p^DZ^_e _from Equations (41) and (42), leading to:

VgeVe={pgDZpgmin⁡DZ−1}(RSD−cSD)(1−cSD)pgDZRMD(pgtopDZ−pgmin⁡DZ)     (43)
 MathType@MTEF@5@5@+=feaafiart1ev1aaatCvAUfKttLearuWrP9MDH5MBPbIqV92AaeXatLxBI9gBaebbnrfifHhDYfgasaacH8akY=wiFfYdH8Gipec8Eeeu0xXdbba9frFj0=OqFfea0dXdd9vqai=hGuQ8kuc9pgc9s8qqaq=dirpe0xb9q8qiLsFr0=vr0=vr0dc8meaabaqaciaacaGaaeqabaqabeGadaaakeaadaWcaaqaaiabdAfawnaaBaaaleaacqWGNbWzcqWGLbqzaeqaaaGcbaGaemOvay1aaSbaaSqaaiabdwgaLbqabaaaaOGaeyypa0ZaaSaaaeaadaGadaqaamaalaaabaGaemiCaa3aa0baaSqaaiabdEgaNbqaaiabdseaejabdQfaAbaaaOqaaiabdchaWnaaDaaaleaacqWGNbWzcyGGTbqBcqGGPbqAcqGGUbGBaeaacqWGebarcqWGAbGwaaaaaOGaeyOeI0IaeGymaedacaGL7bGaayzFaaWaaSaaaeaacqGGOaakcqWGsbGudaWgaaWcbaGaem4uamLaemiraqeabeaakiabgkHiTiabdogaJnaaBaaaleaacqWGtbWucqWGebaraeqaaOGaeiykaKcabaGaeiikaGIaeGymaeJaeyOeI0Iaem4yam2aaSbaaSqaaiabdofatjabdseaebqabaGccqGGPaqkaaaabaGaemiCaa3aa0baaSqaaiabdEgaNbqaaiabdseaejabdQfaAbaakiabdkfasnaaBaaaleaacqWGnbqtcqWGebaraeqaaOGaeiikaGIaemiCaa3aa0baaSqaaiabdEgaNjabdsha0jabd+gaVjabdchaWbqaaiabdseaejabdQfaAbaakiabgkHiTiabdchaWnaaDaaaleaacqWGNbWzcyGGTbqBcqGGPbqAcqGGUbGBaeaacqWGebarcqWGAbGwaaGccqGGPaqkaaGaaCzcaiaaxMaadaqadaqaaiabisda0iabiodaZaGaayjkaiaawMcaaaaa@7BF9@

where

pgtopDZ=1RMD{1+(cMD−1)(1−RSD)(1−cSD)}     (44)
 MathType@MTEF@5@5@+=feaafiart1ev1aaatCvAUfKttLearuWrP9MDH5MBPbIqV92AaeXatLxBI9gBaebbnrfifHhDYfgasaacH8akY=wiFfYdH8Gipec8Eeeu0xXdbba9frFj0=OqFfea0dXdd9vqai=hGuQ8kuc9pgc9s8qqaq=dirpe0xb9q8qiLsFr0=vr0=vr0dc8meaabaqaciaacaGaaeqabaqabeGadaaakeaacqWGWbaCdaqhaaWcbaGaem4zaCMaemiDaqNaem4Ba8MaemiCaahabaGaemiraqKaemOwaOfaaOGaeyypa0ZaaSaaaeaacqaIXaqmaeaacqWGsbGudaWgaaWcbaGaemyta0KaemiraqeabeaaaaGcdaGadaqaaiabigdaXiabgUcaRmaalaaabaGaeiikaGIaem4yam2aaSbaaSqaaiabd2eanjabdseaebqabaGccqGHsislcqaIXaqmcqGGPaqkcqGGOaakcqaIXaqmcqGHsislcqWGsbGudaWgaaWcbaGaem4uamLaemiraqeabeaakiabcMcaPaqaaiabcIcaOiabigdaXiabgkHiTiabdogaJnaaBaaaleaacqWGtbWucqWGebaraeqaaOGaeiykaKcaaaGaay5Eaiaaw2haaiaaxMaacaWLjaWaaeWaaeaacqaI0aancqaI0aanaiaawIcacaGLPaaaaaa@5A69@

and

pgmin⁡DZ=(RSD−cSD){RMD(1−cSD)−cMD(1−RSD)}     (45).
 MathType@MTEF@5@5@+=feaafiart1ev1aaatCvAUfKttLearuWrP9MDH5MBPbIqV92AaeXatLxBI9gBaebbnrfifHhDYfgasaacH8akY=wiFfYdH8Gipec8Eeeu0xXdbba9frFj0=OqFfea0dXdd9vqai=hGuQ8kuc9pgc9s8qqaq=dirpe0xb9q8qiLsFr0=vr0=vr0dc8meaabaqaciaacaGaaeqabaqabeGadaaakeaacqWGWbaCdaqhaaWcbaGaem4zaCMagiyBa0MaeiyAaKMaeiOBa4gabaGaemiraqKaemOwaOfaaOGaeyypa0ZaaSaaaeaacqGGOaakcqWGsbGudaWgaaWcbaGaem4uamLaemiraqeabeaakiabgkHiTiabdogaJnaaBaaaleaacqWGtbWucqWGebaraeqaaOGaeiykaKcabaWaaiWaaeaacqWGsbGudaWgaaWcbaGaemyta0KaemiraqeabeaakiabcIcaOiabigdaXiabgkHiTiabdogaJnaaBaaaleaacqWGtbWucqWGebaraeqaaOGaeiykaKIaeyOeI0Iaem4yam2aaSbaaSqaaiabd2eanjabdseaebqabaGccqGGOaakcqaIXaqmcqGHsislcqWGsbGudaWgaaWcbaGaem4uamLaemiraqeabeaakiabcMcaPaGaay5Eaiaaw2haaaaacaWLjaGaaCzcamaabmaabaGaeGinaqJaeGynaudacaGLOaGaayzkaaGaeiOla4caaa@5FC3@

Equations (27), (40) and (43) allow the gene-environment interaction factor f_ge _to be written as:

fge2={pgDZpgmin⁡DZ−1}(λDZ−1)RMD(pgtopDZ−pgDZ)     (46).
 MathType@MTEF@5@5@+=feaafiart1ev1aaatCvAUfKttLearuWrP9MDH5MBPbIqV92AaeXatLxBI9gBaebbnrfifHhDYfgasaacH8akY=wiFfYdH8Gipec8Eeeu0xXdbba9frFj0=OqFfea0dXdd9vqai=hGuQ8kuc9pgc9s8qqaq=dirpe0xb9q8qiLsFr0=vr0=vr0dc8meaabaqaciaacaGaaeqabaqabeGadaaakeaacqWGMbGzdaqhaaWcbaGaem4zaCMaemyzaugabaGaeGOmaidaaOGaeyypa0ZaaSaaaeaadaGadaqaamaalaaabaGaemiCaa3aa0baaSqaaiabdEgaNbqaaiabdseaejabdQfaAbaaaOqaaiabdchaWnaaDaaaleaacqWGNbWzcyGGTbqBcqGGPbqAcqGGUbGBaeaacqWGebarcqWGAbGwaaaaaOGaeyOeI0IaeGymaedacaGL7bGaayzFaaaabaGaeiikaGccciGae83UdW2aaSbaaSqaaiabdseaejabdQfaAbqabaGccqGHsislcqaIXaqmcqGGPaqkcqWGsbGudaWgaaWcbaGaemyta0KaemiraqeabeaakiabcIcaOiabdchaWnaaDaaaleaacqWGNbWzcqWG0baDcqWGVbWBcqWGWbaCaeaacqWGebarcqWGAbGwaaGccqGHsislcqWGWbaCdaqhaaWcbaGaem4zaCgabaGaemiraqKaemOwaOfaaOGaeiykaKcaaiaaxMaacaWLjaWaaeWaaeaacqaI0aancqaI2aGnaiaawIcacaGLPaaacqGGUaGlaaa@6824@

The parameter p^DZ^_g_, which defines the form of the genetic model, is then given by:

pgDZpgmin⁡DZ=1+fge2(λDZ−1)RMDpgtopDZ1+fge2(λDZ−1)RMDpgmin⁡DZ     (47).
 MathType@MTEF@5@5@+=feaafiart1ev1aaatCvAUfKttLearuWrP9MDH5MBPbIqV92AaeXatLxBI9gBaebbnrfifHhDYfgasaacH8akY=wiFfYdH8Gipec8Eeeu0xXdbba9frFj0=OqFfea0dXdd9vqai=hGuQ8kuc9pgc9s8qqaq=dirpe0xb9q8qiLsFr0=vr0=vr0dc8meaabaqaciaacaGaaeqabaqabeGadaaakeaadaWcaaqaaiabdchaWnaaDaaaleaacqWGNbWzaeaacqWGebarcqWGAbGwaaaakeaacqWGWbaCdaqhaaWcbaGaem4zaCMagiyBa0MaeiyAaKMaeiOBa4gabaGaemiraqKaemOwaOfaaaaakiabg2da9maalaaabaGaeGymaeJaey4kaSIaemOzay2aa0baaSqaaiabdEgaNjabdwgaLbqaaiabikdaYaaakiabcIcaOGGaciab=T7aSnaaBaaaleaacqWGebarcqWGAbGwaeqaaOGaeyOeI0IaeGymaeJaeiykaKIaemOuai1aaSbaaSqaaiabd2eanjabdseaebqabaGccqWGWbaCdaqhaaWcbaGaem4zaCMaemiDaqNaem4Ba8MaemiCaahabaGaemiraqKaemOwaOfaaaGcbaGaeGymaeJaey4kaSIaemOzay2aa0baaSqaaiabdEgaNjabdwgaLbqaaiabikdaYaaakiabcIcaOiab=T7aSnaaBaaaleaacqWGebarcqWGAbGwaeqaaOGaeyOeI0IaeGymaeJaeiykaKIaemOuai1aaSbaaSqaaiabd2eanjabdseaebqabaGccqWGWbaCdaqhaaWcbaGaem4zaCMagiyBa0MaeiyAaKMaeiOBa4gabaGaemiraqKaemOwaOfaaaaakiaaxMaacaWLjaWaaeWaaeaacqaI0aancqaI3aWnaiaawIcacaGLPaaacqGGUaGlaaa@79C0@

For known R_MD_, R_SD _and λ_DZ _a solution space can now be mapped, which includes all possible variances consistent with the data and with the inequalities derived above.

Requiring the variances to be positive leads to the additional conditions on p^DZ^_g _and c_SD _shown in Table 3.

**Table 3:** Further constraints on model parameters

**Condition**	**Limits on p**^**DZ**^_**g**_	**Limits on c**_**SD**_
*V*_*e *_≥ 0	pgDZ≤pgtopDZ MathType@MTEF@5@5@+=feaafiart1ev1aaatCvAUfKttLearuWrP9MDH5MBPbIqV92AaeXatLxBI9gBaebbnrfifHhDYfgasaacH8akY=wiFfYdH8Gipec8Eeeu0xXdbba9frFj0=OqFfea0dXdd9vqai=hGuQ8kuc9pgc9s8qqaq=dirpe0xb9q8qiLsFr0=vr0=vr0dc8meaabaqaciaacaGaaeqabaqabeGadaaakeaacqWGWbaCdaqhaaWcbaGaem4zaCgabaGaemiraqKaemOwaOfaaOGaeyizImQaemiCaa3aa0baaSqaaiabdEgaNjabdsha0jabd+gaVjabdchaWbqaaiabdseaejabdQfaAbaaaaa@3D22@	

*V*_*ge *_≥ 0	pgDZ≥pgmin⁡DZ MathType@MTEF@5@5@+=feaafiart1ev1aaatCvAUfKttLearuWrP9MDH5MBPbIqV92AaeXatLxBI9gBaebbnrfifHhDYfgasaacH8akY=wiFfYdH8Gipec8Eeeu0xXdbba9frFj0=OqFfea0dXdd9vqai=hGuQ8kuc9pgc9s8qqaq=dirpe0xb9q8qiLsFr0=vr0=vr0dc8meaabaqaciaacaGaaeqabaqabeGadaaakeaacqWGWbaCdaqhaaWcbaGaem4zaCgabaGaemiraqKaemOwaOfaaOGaeyyzImRaemiCaa3aa0baaSqaaiabdEgaNjGbc2gaTjabcMgaPjabc6gaUbqaaiabdseaejabdQfaAbaaaaa@3D14@	

*V*_*g *_≥ 0		*C*_*SD *_≤ *R*_*SD*_

*γ*_max _≥ *γ*_min_		If *λ*_*MD *_> *y*_*e *_+ 1 require: *c*_*SD *_≥ *c*_*SDm *_where cSDm=1−(λDZ−1)(1−RSD)[cMD+fge2(λDZ−1)RMD+yefge2(cMD−1)][1+fge2(λDZ−1)][(λDZ−1)RMD−ye] MathType@MTEF@5@5@+=feaafiart1ev1aaatCvAUfKttLearuWrP9MDH5MBPbIqV92AaeXatLxBI9gBaebbnrfifHhDYfgasaacH8akY=wiFfYdH8Gipec8Eeeu0xXdbba9frFj0=OqFfea0dXdd9vqai=hGuQ8kuc9pgc9s8qqaq=dirpe0xb9q8qiLsFr0=vr0=vr0dc8meaabaqaciaacaGaaeqabaqabeGadaaakeaacqWGJbWydaWgaaWcbaGaem4uamLaemiraqKaemyBa0gabeaakiabg2da9iabigdaXiabgkHiTmaalaaabaWaaeWaaeaaiiGacqWF7oaBdaWgaaWcbaGaemiraqKaemOwaOfabeaakiabgkHiTiabigdaXaGaayjkaiaawMcaamaabmaabaGaeGymaeJaeyOeI0IaemOuai1aaSbaaSqaaiabdofatjabdseaebqabaaakiaawIcacaGLPaaadaWadaqaaiabdogaJnaaBaaaleaacqWGnbqtcqWGebaraeqaaOGaey4kaSIaemOzay2aa0baaSqaaiabdEgaNjabdwgaLbqaaiabikdaYaaakmaabmaabaGae83UdW2aaSbaaSqaaiabdseaejabdQfaAbqabaGccqGHsislcqaIXaqmaiaawIcacaGLPaaacqWGsbGudaWgaaWcbaGaemyta0KaemiraqeabeaakiabgUcaRiabdMha5naaBaaaleaacqWGLbqzaeqaaOGaemOzay2aa0baaSqaaiabdEgaNjabdwgaLbqaaiabikdaYaaakmaabmaabaGaem4yam2aaSbaaSqaaiabd2eanjabdseaebqabaGccqGHsislcqaIXaqmaiaawIcacaGLPaaaaiaawUfacaGLDbaaaeaadaWadaqaaiabigdaXiabgUcaRiabdAgaMnaaDaaaleaacqWGNbWzcqWGLbqzaeaacqaIYaGmaaGcdaqadaqaaiab=T7aSnaaBaaaleaacqWGebarcqWGAbGwaeqaaOGaeyOeI0IaeGymaedacaGLOaGaayzkaaaacaGLBbGaayzxaaWaamWaaeaadaqadaqaaiab=T7aSnaaBaaaleaacqWGebarcqWGAbGwaeqaaOGaeyOeI0IaeGymaedacaGLOaGaayzkaaGaemOuai1aaSbaaSqaaiabd2eanjabdseaebqabaGccqGHsislcqWG5bqEdaWgaaWcbaGaemyzaugabeaaaOGaay5waiaaw2faaaaaaaa@8C1F@

The limits on U_ge _shown in Table 2 set limits on the range of gene-environment interaction models such that:

−ε(1−ε)PAFeE≤fge≤1PAFeE     (48)
 MathType@MTEF@5@5@+=feaafiart1ev1aaatCvAUfKttLearuWrP9MDH5MBPbIqV92AaeXatLxBI9gBaebbnrfifHhDYfgasaacH8akY=wiFfYdH8Gipec8Eeeu0xXdbba9frFj0=OqFfea0dXdd9vqai=hGuQ8kuc9pgc9s8qqaq=dirpe0xb9q8qiLsFr0=vr0=vr0dc8meaabaqaciaacaGaaeqabaqabeGadaaakeaacqGHsisldaWcaaqaaGGaciab=v7aLbqaaiabcIcaOiabigdaXiabgkHiTiab=v7aLjabcMcaPiabdcfaqjabdgeabjabdAeagnaaDaaaleaacqWGLbqzaeaacqWGfbqraaaaaOGaeyizImQaemOzay2aaSbaaSqaaiabdEgaNjabdwgaLbqabaGccqGHKjYOdaWcaaqaaiabigdaXaqaaiabdcfaqjabdgeabjabdAeagnaaDaaaleaacqWGLbqzaeaacqWGfbqraaaaaOGaaCzcaiaaxMaadaqadaqaaiabisda0iabiIda4aGaayjkaiaawMcaaaaa@4DB4@

Noting that f_ge _= 0 corresponds to p^DZ^_g _= p^DZ^_gmin _(Equation (64)), this implies that, for U_ge _≥ 0, the solution space may be defined by:

pgmin⁡DZ≤pgDZ≤pgmax⁡DZ     (49)
 MathType@MTEF@5@5@+=feaafiart1ev1aaatCvAUfKttLearuWrP9MDH5MBPbIqV92AaeXatLxBI9gBaebbnrfifHhDYfgasaacH8akY=wiFfYdH8Gipec8Eeeu0xXdbba9frFj0=OqFfea0dXdd9vqai=hGuQ8kuc9pgc9s8qqaq=dirpe0xb9q8qiLsFr0=vr0=vr0dc8meaabaqaciaacaGaaeqabaqabeGadaaakeaacqWGWbaCdaqhaaWcbaGaem4zaCMagiyBa0MaeiyAaKMaeiOBa4gabaGaemiraqKaemOwaOfaaOGaeyizImQaemiCaa3aa0baaSqaaiabdEgaNbqaaiabdseaejabdQfaAbaakiabgsMiJkabdchaWnaaDaaaleaacqWGNbWzcyGGTbqBcqGGHbqycqGG4baEaeaacqWGebarcqWGAbGwaaGccaWLjaGaaCzcamaabmaabaGaeGinaqJaeGyoaKdacaGLOaGaayzkaaaaaa@4CF0@

where p^DZ^_gmax _is given by Equation (47) with f_ge _= 1/PAF^E^_e_.

For U_ge _≤ 0, the solution space may be defined by:

pgmin⁡DZ≤pgDZ≤pgnegDZ     (50)
 MathType@MTEF@5@5@+=feaafiart1ev1aaatCvAUfKttLearuWrP9MDH5MBPbIqV92AaeXatLxBI9gBaebbnrfifHhDYfgasaacH8akY=wiFfYdH8Gipec8Eeeu0xXdbba9frFj0=OqFfea0dXdd9vqai=hGuQ8kuc9pgc9s8qqaq=dirpe0xb9q8qiLsFr0=vr0=vr0dc8meaabaqaciaacaGaaeqabaqabeGadaaakeaacqWGWbaCdaqhaaWcbaGaem4zaCMagiyBa0MaeiyAaKMaeiOBa4gabaGaemiraqKaemOwaOfaaOGaeyizImQaemiCaa3aa0baaSqaaiabdEgaNbqaaiabdseaejabdQfaAbaakiabgsMiJkabdchaWnaaDaaaleaacqWGNbWzcqWGUbGBcqWGLbqzcqWGNbWzaeaacqWGebarcqWGAbGwaaGccaWLjaGaaCzcamaabmaabaGaeGynauJaeGimaadacaGLOaGaayzkaaaaaa@4CC9@

where p^DZ^_gneg _is given by Equation (47) with f_ge _= -ε/(1-ε)PAF^E^_e_.

The remaining limits on U_ge _lead to the additional conditions on the range of γ values (the proportion of the population in the high risk group) shown in Table 2. These conditions on γ may be written:

*γ*_min _≤ *γ *≤ *γ*_max _    (51)

where (noting that γ_maxge _= γ_o _when f_ge _= 1):

γmax⁡={γmax⁡ge for fge≥1γ0 for fge≤1     (52)
 MathType@MTEF@5@5@+=feaafiart1ev1aaatCvAUfKttLearuWrP9MDH5MBPbIqV92AaeXatLxBI9gBaebbnrfifHhDYfgasaacH8akY=wiFfYdH8Gipec8Eeeu0xXdbba9frFj0=OqFfea0dXdd9vqai=hGuQ8kuc9pgc9s8qqaq=dirpe0xb9q8qiLsFr0=vr0=vr0dc8meaabaqaciaacaGaaeqabaqabeGadaaakeaaiiGacqWFZoWzdaWgaaWcbaGagiyBa0MaeiyyaeMaeiiEaGhabeaakiabg2da9maaceaabaqbaeqabiqaaaqaaiab=n7aNnaaBaaaleaacyGGTbqBcqGGHbqycqGG4baEcqWGNbWzcqWGLbqzaeqaaOGaeeiiaaIaeeOzayMaee4Ba8MaeeOCaiNaeeiiaaIaemOzay2aaSbaaSqaaiabdEgaNjabdwgaLbqabaGccqGHLjYScqaIXaqmaeaacqWFZoWzdaWgaaWcbaGaeGimaadabeaakiabbccaGiabbAgaMjabb+gaVjabbkhaYjabbccaGiabdAgaMnaaBaaaleaacqWGNbWzcqWGLbqzaeqaaOGaeyizImQaeGymaedaaaGaay5EaaGaaCzcaiaaxMaadaqadaqaaiabiwda1iabikdaYaGaayjkaiaawMcaaaaa@5E2F@

and (noting that γ_minge _= γ_neg _when f_ge _= -r_t_/(1-r_t_)):

γmin⁡={γmin⁡ge for fge≥−rt/(1−rt)γneg for fge≤−rt/(1−rt)     (53)
 MathType@MTEF@5@5@+=feaafiart1ev1aaatCvAUfKttLearuWrP9MDH5MBPbIqV92AaeXatLxBI9gBaebbnrfifHhDYfgasaacH8akY=wiFfYdH8Gipec8Eeeu0xXdbba9frFj0=OqFfea0dXdd9vqai=hGuQ8kuc9pgc9s8qqaq=dirpe0xb9q8qiLsFr0=vr0=vr0dc8meaabaqaciaacaGaaeqabaqabeGadaaakeaaiiGacqWFZoWzdaWgaaWcbaGagiyBa0MaeiyAaKMaeiOBa4gabeaakiabg2da9maaceaabaqbaeqabiqaaaqaaiab=n7aNnaaBaaaleaacyGGTbqBcqGGPbqAcqGGUbGBcqWGNbWzcqWGLbqzaeqaaOGaeeiiaaIaeeOzayMaee4Ba8MaeeOCaiNaeeiiaaIaemOzay2aaSbaaSqaaiabdEgaNjabdwgaLbqabaGccqGHLjYSdaWcgaqaaiabgkHiTiabdkhaYnaaBaaaleaacqWG0baDaeqaaaGcbaWaaeWaaeaacqaIXaqmcqGHsislcqWGYbGCdaWgaaWcbaGaemiDaqhabeaaaOGaayjkaiaawMcaaaaaaeaacqWFZoWzdaWgaaWcbaGaemOBa4MaemyzauMaem4zaCgabeaakiabbccaGiabbAgaMjabb+gaVjabbkhaYjabbccaGiabdAgaMnaaBaaaleaacqWGNbWzcqWGLbqzaeqaaOGaeyizIm6aaSGbaeaacqGHsislcqWGYbGCdaWgaaWcbaGaemiDaqhabeaaaOqaamaabmaabaGaeGymaeJaeyOeI0IaemOCai3aaSbaaSqaaiabdsha0bqabaaakiaawIcacaGLPaaaaaaaaaGaay5EaaGaaCzcaiaaxMaadaqadaqaaiabiwda1iabiodaZaGaayjkaiaawMcaaaaa@748C@

Two transition lines can therefore be defined such that p^DZ^_g _= p^DZ^_gt _when f_ge _= 1 and p^DZ^_g _= p^DZ^_gnegt _when f_ge _= -r_t_/(1-r_t_). The values of p^DZ^_gt _and p^DZ^_gnegt _may be calculated using Equation (47).

The full range of gene-environment interaction models specified by f_ge _(within the limits given by Equation (48)) and the corresponding range of γ values are summarized in Table 4. Note that the risk distribution associated with f_ge _= 1 corresponds to a multiplicative model of gene-environment interaction. If f_ge _≥ 1 solutions with population impact PI = 1 may exist (i.e. with PAF^E^_ge _= PAF^E^_e_), provided the proportion of the population in the high risk genotypic group takes the maximum value consistent with the data (γ = γ_maxge_). For lower values of f_ge_, solutions with PI = 1 cannot exist.

**Table 4:** Limits on the gene-environment interaction factor (f_ge_) and the proportion of the population in the high-genotypic risk group (γ).

**Gene-environment interaction model**	**Interaction factor f**_**ge**_	**Risk distribution**		**Utility U**_**ge**_	**Fraction of population at high genotypic risk**	
					
					**Maximum γ**_**max**_	**Minimum γ**_**min**_
Genetic effect in high-exposure group only	1/PAF^E^_e_	**R**_**00**_	**R**_**ge**_	Positive	γ_maxge _(where PAF^E^_ge _= PAF^E^_e_; PI = 1; and U_ge _= 1-γ).	γ_minge _(where R_ge _= 1).
		**R**_**00**_	**R**_**0e**_			
Multiplicative	1	**R**_**g0**_	**R**_**g0**_**R**_**0e**_**/R**_**00**_		γ_maxge _= γ_0 _(where PAF^E^_ge _= PAF^E^_e_; R_00 _= 0; and PAF^G^_g _= 1).	
		**R**_**00**_	**R**_**0e**_			
Additive	0	**R**_**g0**_	**R**_**g0**_**+R**_**0e**_**-R**_**00**_	Zero	γ_0 _(where R_00 _= 0).	
		**R**_**00**_	**R**_**0e**_			
Reverse multiplicative	-r_t_/(1-r_t_)	**R**_**g0**_	**(1-R_g0_) (1-R_0e_)/(1-R**_**00**_**)**	Negative		γ_neg _= γ_minge _(where PAF^E^_ge _= 0 and R_ge _= 1)
		**R**_**00**_	**R**_**0e**_			
Genetic effect in low-exposure group only	-ε/(1-ε)PAF^E^_e_	**R**_**g0**_	**R**_**0e**_			γ_neg _(where PAF^E^_ge _= 0 and PI = 0).
		**R**_**00**_	**R**_**0e**_			

One additional condition is necessary for solutions to exist, namely:

*γ*_max _≥ *γ*_min _    (54)

This condition is always met if

*λ*_*MD *_≤ *y*_*e *_+ 1     (55)

where

ye={F1/fge for fge≥1F1/F2 for 1≥fge≥−rt/(1−rt)−F2/fge for fge≤−rt/(1−rt)     (56)
 MathType@MTEF@5@5@+=feaafiart1ev1aaatCvAUfKttLearuWrP9MDH5MBPbIqV92AaeXatLxBI9gBaebbnrfifHhDYfgasaacH8akY=wiFfYdH8Gipec8Eeeu0xXdbba9frFj0=OqFfea0dXdd9vqai=hGuQ8kuc9pgc9s8qqaq=dirpe0xb9q8qiLsFr0=vr0=vr0dc8meaabaqaciaacaGaaeqabaqabeGadaaakeaacqWG5bqEdaWgaaWcbaGaemyzaugabeaakiabg2da9maaceaabaqbaeqabmqaaaqaamaalyaabaGaemOray0aaSbaaSqaaiabigdaXaqabaaakeaacqWGMbGzdaWgaaWcbaGaem4zaCMaemyzaugabeaaaaGccqqGGaaicqqGMbGzcqqGVbWBcqqGYbGCcqqGGaaicqWGMbGzdaWgaaWcbaGaem4zaCMaemyzaugabeaakiabgwMiZkabigdaXaqaamaalyaabaGaemOray0aaSbaaSqaaiabigdaXaqabaaakeaacqWGgbGrdaWgaaWcbaGaeGOmaidabeaaaaGccqqGGaaicqqGMbGzcqqGVbWBcqqGYbGCcqqGGaaicqaIXaqmcqGHLjYScqWGMbGzdaWgaaWcbaGaem4zaCMaemyzaugabeaakiabgwMiZoaalyaabaGaeyOeI0IaemOCai3aaSbaaSqaaiabdsha0bqabaaakeaadaqadaqaaiabigdaXiabgkHiTiabdkhaYnaaBaaaleaacqWG0baDaeqaaaGccaGLOaGaayzkaaaaaaqaamaalyaabaGaeyOeI0IaemOray0aaSbaaSqaaiabikdaYaqabaaakeaacqWGMbGzdaWgaaWcbaGaem4zaCMaemyzaugabeaaaaGccqqGGaaicqqGMbGzcqqGVbWBcqqGYbGCcqqGGaaicqWGMbGzdaWgaaWcbaGaem4zaCMaemyzaugabeaakiabgsMiJoaalyaabaGaeyOeI0IaemOCai3aaSbaaSqaaiabdsha0bqabaaakeaadaqadaqaaiabigdaXiabgkHiTiabdkhaYnaaBaaaleaacqWG0baDaeqaaaGccaGLOaGaayzkaaaaaaaaaiaawUhaaiaaxMaacaWLjaWaaeWaaeaacqaI1aqncqaI2aGnaiaawIcacaGLPaaaaaa@84E2@

and F_1 _and F_2 _are given by:

F1=[(1−rtrt)−(1−εε)PAFeE][1+fge(1−εε)PAFeE]=(1−re)[rt+fge(re−rt)]     (57)
 MathType@MTEF@5@5@+=feaafiart1ev1aaatCvAUfKttLearuWrP9MDH5MBPbIqV92AaeXatLxBI9gBaebbnrfifHhDYfgasaacH8akY=wiFfYdH8Gipec8Eeeu0xXdbba9frFj0=OqFfea0dXdd9vqai=hGuQ8kuc9pgc9s8qqaq=dirpe0xb9q8qiLsFr0=vr0=vr0dc8meaabaqaciaacaGaaeqabaqabeGadaaakeaacqWGgbGrdaWgaaWcbaGaeGymaedabeaakiabg2da9maalaaabaWaamWaaeaadaqadaqaamaalaaabaGaeGymaeJaeyOeI0IaemOCai3aaSbaaSqaaiabdsha0bqabaaakeaacqWGYbGCdaWgaaWcbaGaemiDaqhabeaaaaaakiaawIcacaGLPaaacqGHsisldaqadaqaamaalaaabaGaeGymaeJaeyOeI0ccciGae8xTdugabaGae8xTdugaaaGaayjkaiaawMcaaiabdcfaqjabdgeabjabdAeagnaaDaaaleaacqWGLbqzaeaacqWGfbqraaaakiaawUfacaGLDbaaaeaadaWadaqaaiabigdaXiabgUcaRiabdAgaMnaaBaaaleaacqWGNbWzcqWGLbqzaeqaaOWaaeWaaeaadaWcaaqaaiabigdaXiabgkHiTiab=v7aLbqaaiab=v7aLbaaaiaawIcacaGLPaaacqWGqbaucqWGbbqqcqWGgbGrdaqhaaWcbaGaemyzaugabaGaemyraueaaaGccaGLBbGaayzxaaaaaiabg2da9maaleaabaGaeiikaGIaeGymaeJaeyOeI0IaemOCai3aaSbaaSqaaiabdwgaLbGcbeaacqGGPaqkaeaadaWadaqaaiabdkhaYTWaaSbaaeaacqWG0baDaeqaaOGaey4kaSIaemOzay2cdaWgaaqaaiabdEgaNjabdwgaLbqabaGccqGGOaakcqWGYbGCdaWgaaWcbaGaemyzaugakeqaaiabgkHiTiabdkhaYTWaaSbaaeaacqWG0baDaeqaaOGaeiykaKcaliaawUfacaGLDbaaaaGccaWLjaGaaCzcamaabmaabaGaeGynauJaeG4naCdacaGLOaGaayzkaaaaaa@7CF7@

F2=(1−PAFeE)(1−fgePAFeE)     (58).
 MathType@MTEF@5@5@+=feaafiart1ev1aaatCvAUfKttLearuWrP9MDH5MBPbIqV92AaeXatLxBI9gBaebbnrfifHhDYfgasaacH8akY=wiFfYdH8Gipec8Eeeu0xXdbba9frFj0=OqFfea0dXdd9vqai=hGuQ8kuc9pgc9s8qqaq=dirpe0xb9q8qiLsFr0=vr0=vr0dc8meaabaqaciaacaGaaeqabaqabeGadaaakeaacqWGgbGrdaWgaaWcbaGaeGOmaidabeaakiabg2da9maalaaabaWaaeWaaeaacqaIXaqmcqGHsislcqWGqbaucqWGbbqqcqWGgbGrdaqhaaWcbaGaemyzaugabaGaemyraueaaaGccaGLOaGaayzkaaaabaWaaeWaaeaacqaIXaqmcqGHsislcqWGMbGzdaWgaaWcbaGaem4zaCMaemyzaugabeaakiabdcfaqjabdgeabjabdAeagnaaDaaaleaacqWGLbqzaeaacqWGfbqraaaakiaawIcacaGLPaaaaaGaaCzcaiaaxMaadaqadaqaaiabiwda1iabiIda4aGaayjkaiaawMcaaiabc6caUaaa@4C73@

However, if λ_MD _is greater than this, the requirement γ_max _≥ γ_min _further restricts the values of c_SD _that lie within the solution space (Table 3).

If V_e _and ε are known, a solution space can be now be mapped for p^DZ^_g _and f_ge _with known input data from twin and sibling studies (λ_MZ_, λ_DZ _and λ_sib_), for a given c_MD _and all values of c_SD _within the assumed range. The boundaries of the solution space are determined by the limits on f_ge _given by Equation (48), the condition γ_max _≥ γ_min _(Equation (54)), and the requirement that p^DZ^_g _is less than or equal to 1/2 (Equation (20)) – no other condition on the genetic model is specified a priori. For each genetic risk model and gene-environment interaction model in the solution space, defined by p^DZ^_g _and f_ge _respectively, the variances V_g _and V_ge _can then be calculated, as can γ_max _and γ_min_. For a chosen γ value in the allowed range, U_ge _can then be calculated from Equation (28).

The model code is available as [Additional file 1: heritability12.xls].

Note that the condition on p^DZ^_g _≤ 1/2 may also be rewritten using Equation (47), so that:

pgDZ≤1/2⇒(pgmin⁡DZ−12)pgmin⁡DZ≤RMDfe2(λDZ−1)(1/2−pgtopDZ)     (59)
 MathType@MTEF@5@5@+=feaafiart1ev1aaatCvAUfKttLearuWrP9MDH5MBPbIqV92AaeXatLxBI9gBaebbnrfifHhDYfgasaacH8akY=wiFfYdH8Gipec8Eeeu0xXdbba9frFj0=OqFfea0dXdd9vqai=hGuQ8kuc9pgc9s8qqaq=dirpe0xb9q8qiLsFr0=vr0=vr0dc8meaabaqaciaacaGaaeqabaqabeGadaaakeaacqWGWbaCdaqhaaWcbaGaem4zaCgabaGaemiraqKaemOwaOfaaOGaeyizImQaeGymaeJaei4la8IaeGOmaiJaeyO0H49aaSaaaeaadaqadaqaaiabdchaWnaaDaaaleaacqWGNbWzcyGGTbqBcqGGPbqAcqGGUbGBaeaacqWGebarcqWGAbGwaaGccqGHsisldaWcaaqaaiabigdaXaqaaiabikdaYaaaaiaawIcacaGLPaaaaeaacqWGWbaCdaqhaaWcbaGaem4zaCMagiyBa0MaeiyAaKMaeiOBa4gabaGaemiraqKaemOwaOfaaaaakiabgsMiJkabdkfasnaaBaaaleaacqWGnbqtcqWGebaraeqaaOGaemOzay2aa0baaSqaaiabdwgaLbqaaiabikdaYaaakmaabmaabaacciGae83UdW2aaSbaaSqaaiabdseaejabdQfaAbqabaGccqGHsislcqaIXaqmaiaawIcacaGLPaaadaqadaqaamaalyaabaGaeGymaedabaGaeGOmaiJaeyOeI0IaemiCaa3aa0baaSqaaiabdEgaNjabdsha0jabd+gaVjabdchaWbqaaiabdseaejabdQfaAbaaaaaakiaawIcacaGLPaaacaWLjaGaaCzcamaabmaabaGaeGynauJaeGyoaKdacaGLOaGaayzkaaaaaa@738C@

which is always met if

pgtopDZ≤1/2     (60).
 MathType@MTEF@5@5@+=feaafiart1ev1aaatCvAUfKttLearuWrP9MDH5MBPbIqV92AaeXatLxBI9gBaebbnrfifHhDYfgasaacH8akY=wiFfYdH8Gipec8Eeeu0xXdbba9frFj0=OqFfea0dXdd9vqai=hGuQ8kuc9pgc9s8qqaq=dirpe0xb9q8qiLsFr0=vr0=vr0dc8meaabaqaciaacaGaaeqabaqabeGadaaakeaacqWGWbaCdaqhaaWcbaGaem4zaCMaemiDaqNaem4Ba8MaemiCaahabaGaemiraqKaemOwaOfaaOGaeyizImQaeGymaeJaei4la8IaeGOmaiJaaCzcaiaaxMaadaqadaqaaiabiAda2iabicdaWaGaayjkaiaawMcaaiabc6caUaaa@4048@

Before mapping the solution space, first consider some special cases and a comparison of the model with the classical twin studies approach.

### Special cases

#### 1. No genetic variance

If V_g _= 0, Equation (27) implies that V_ge _= 0 also. Equations (29), (30) and (31) then give:

*R*_*SD *_= *c*_*SD *_    (61)

and

*R*_*MD *_= *c*_*MD *_    (62)

Under the usual assumption that c_MD _= 1 (the 'equal environments' assumption), this is the well-known result that genetic variance can be zero only when the concordance in monozygotic and dizygotic twins is the same (leading to R_MD _= 1). However, if the equal environments assumption is not met (c_MD _> 1), values of R_MD _greater than 1 do not necessarily imply that a genetic component to the variance exists (see, for example, [18]).

#### 2. No environmental variance

If V_e _= 0, Equation (27) implies that V_ge _= 0 also. Equations (29), (30) and (31) then give:

*R*_*SD *_= 1     (63)

and

RMD=1/pgDZ     (64)
 MathType@MTEF@5@5@+=feaafiart1ev1aaatCvAUfKttLearuWrP9MDH5MBPbIqV92AaeXatLxBI9gBaebbnrfifHhDYfgasaacH8akY=wiFfYdH8Gipec8Eeeu0xXdbba9frFj0=OqFfea0dXdd9vqai=hGuQ8kuc9pgc9s8qqaq=dirpe0xb9q8qiLsFr0=vr0=vr0dc8meaabaqaciaacaGaaeqabaqabeGadaaakeaacqWGsbGudaWgaaWcbaGaemyta0Kaemiraqeabeaakiabg2da9maalyaabaGaeGymaedabaGaemiCaa3aa0baaSqaaiabdEgaNbqaaiabdseaejabdQfaAbaaaaGccaWLjaGaaCzcamaabmaabaGaeGOnayJaeGinaqdacaGLOaGaayzkaaaaaa@3C51@

For a purely genetic model with no environmental variance, Equation (64) implies that if R_MD _> 2, p^DZ^_g _< 1/2. This is consistent with Risch's finding [16] that neither an additive genetic model nor a single dominant gene model (both with p^DZ^_g _= 1/2) can fit the data for conditions such as schizophrenia (which has an R_MD _value significantly greater than 2).

#### 3. Classical twin study assumptions

Assuming no gene-environment interaction (V_ge _= 0); an additive genetic risk model (p^DZ^_g _= 1/2); and the 'equal environments' assumption (c_MD _= 1) in Equations (29), (30) and (31) gives:

Vgrt2=2(λMZ−λDZ)     (65)
 MathType@MTEF@5@5@+=feaafiart1ev1aaatCvAUfKttLearuWrP9MDH5MBPbIqV92AaeXatLxBI9gBaebbnrfifHhDYfgasaacH8akY=wiFfYdH8Gipec8Eeeu0xXdbba9frFj0=OqFfea0dXdd9vqai=hGuQ8kuc9pgc9s8qqaq=dirpe0xb9q8qiLsFr0=vr0=vr0dc8meaabaqaciaacaGaaeqabaqabeGadaaakeaadaWcaaqaaiabdAfawnaaBaaaleaacqWGNbWzaeqaaaGcbaGaemOCai3aa0baaSqaaiabdsha0bqaaiabikdaYaaaaaGccqGH9aqpcqaIYaGmdaqadaqaaGGaciab=T7aSnaaBaaaleaacqWGnbqtcqWGAbGwaeqaaOGaeyOeI0Iae83UdW2aaSbaaSqaaiabdseaejabdQfaAbqabaaakiaawIcacaGLPaaacaWLjaGaaCzcamaabmaabaGaeGOnayJaeGynaudacaGLOaGaayzkaaaaaa@4536@

This is the classical twin study result, assuming the dominance term of the genetic variance is negligible. Note that, if R_MD _= 2, the classical solution implies that the environmental variance terms in Equations (29) to (31) are zero and shared sibling risk is due to entirely to shared genes.

#### 4. No correlation in genotypic risk in siblings (p^DZ^_g _= 0)

Equation (20) allows p^DZ^_g _to tend to zero. Substituting p^DZ^_g _= 0 in Equations (29), (30) and (31) and using the definition of the gene-environment interaction factor (Equation (28)) gives:

*R*_*SD *_= *c*_*SD *_    (66)

and

Vgrt2=(λDZ−1)(RMD−cMD)[1+fge2cMD(λDZ−1)]     (67)
 MathType@MTEF@5@5@+=feaafiart1ev1aaatCvAUfKttLearuWrP9MDH5MBPbIqV92AaeXatLxBI9gBaebbnrfifHhDYfgasaacH8akY=wiFfYdH8Gipec8Eeeu0xXdbba9frFj0=OqFfea0dXdd9vqai=hGuQ8kuc9pgc9s8qqaq=dirpe0xb9q8qiLsFr0=vr0=vr0dc8meaabaqaciaacaGaaeqabaqabeGadaaakeaadaWcaaqaaiabdAfawnaaBaaaleaacqWGNbWzaeqaaaGcbaGaemOCai3aa0baaSqaaiabdsha0bqaaiabikdaYaaaaaGccqGH9aqpdaWcaaqaamaabmaabaacciGae83UdW2aaSbaaSqaaiabdseaejabdQfaAbqabaGccqGHsislcqaIXaqmaiaawIcacaGLPaaadaqadaqaaiabdkfasnaaBaaaleaacqWGnbqtcqWGebaraeqaaOGaeyOeI0Iaem4yam2aaSbaaSqaaiabd2eanjabdseaebqabaaakiaawIcacaGLPaaaaeaadaWadaqaaiabigdaXiabgUcaRiabdAgaMnaaDaaaleaacqWGNbWzcqWGLbqzaeaacqaIYaGmaaGccqWGJbWydaWgaaWcbaGaemyta0KaemiraqeabeaakmaabmaabaGae83UdW2aaSbaaSqaaiabdseaejabdQfaAbqabaGccqGHsislcqaIXaqmaiaawIcacaGLPaaaaiaawUfacaGLDbaaaaGaaCzcaiaaxMaadaqadaqaaiabiAda2iabiEda3aGaayjkaiaawMcaaaaa@5F07@

Note that, from Equations (30) and (31), p^DZ^_g _= 0 corresponds to a purely environmental explanation for shared sibling risks (although there may remain a genetic component to shared risks in monozygotic twins, from Equation (29)). The solution p^DZ^_g _= 0 may not exist in reality; however, the solution at this limit is of interest because low values of p^DZ^_g _are plausible.

Also, note that if f_ge _= 0 (no gene-environment interaction) and c_MD _= 1 (the 'equal environments' assumption), the genetic variance V_g _given by Equation (67) is half the classical twin study result (Equation (65)).

#### 5. Cases where γ_max _= γ_min_

If the line γ_max _= γ_min _exists within the solution space, some special cases may arise with risk distributions of particular interest (including, for example, a solution with R_ge _= 1 and all other risks zero). These special cases and the conditions that they meet are shown in Table 5.

**Table 5:** Special cases with γ_max _= γ_min_for U_ge _≥ 0

**Special cases with γ**_**max **_**= γ**_**min**_				**Special cases with γ**_**max **_**= γ**_**min **_**and specific G-E interaction models**				**Special cases with γ**_**max **_**= γ**_**min **_**and all risks all 0 or 1**			
**Risk distribution**		**Conditions**	**Population impact and Utility**	**Risk distribution**		**Conditions**	**Population impact and Utility**	**Risk distribution**		**Conditions**	**Population impact and Utility**

								1	1	r_t _= 1 PAF_e _= 0	Undefined (PAF_ge _= 0)
				R_00_	1	γ_minge _= γ_maxge _(R_ge _= 1 and PAF_ge _= PAF_e_) f_ge _= 1/PAF_e_	PI = 1 U_ge _= 1-γ	1	1		
R_g0_	1	γ_minge _= γ_maxge _(R_ge _= 1 and PAF_ge _= PAF_e_) f_ge _≥ 1	PI = 1 U_ge _= 1-γ	R_00_	R_00_			0	1	r_t _= γε PAF_e _= 1	PI = 1 U_ge _= 1-γ
R_00_	R_00_			R_g0_	1	γ_minge _= γ_0 _= γ_maxge _(R_ge _= 1; R_00 _= 0; PAF_ge _= PAF_e_) f_ge _= 1	PI = 1 U_ge _= 1-γ	0	0		
R_g0_	1	γ_minge _= γ_0 _(R_ge _= 1; R_00 _= 0) 0 ≤ f_ge _≤ 1	0 = PI = 1 U_ge _= PI-γ	0	0			1	1	r_t _= γ PAF_e _= 0	Undefined (PAF_ge _= 0)
0	R_0e_			1-R_0e_	1	γ_minge _= γ_0 _(R_ge _= 1; R_00 _= 0) f_ge _= 0	PI = γ U_ge _= 0	0	0		
				0	R_0e_			0	1	r_t _= ε PAF_e _= 1	PI = γ U_ge _= 0
								0	1		

#### 6. Cases where γ_maxge _< 1/2

Equation (27) shows that for a fixed gene-environment interaction factor f_ge _and genetic variance component V_g_, the utility U_ge _is maximum when γ = 1/2, i.e. when half the population is in the high genotypic risk group, provided this solution exists. However, if γ_max _< 1/2, utility is maximum when γ = γ_max_. As a smaller proportion of the population is then targeted, these solutions are of particular interest. Because solutions with population impact PI = 1 may exist when 1 ≤ f_ge _≤ 1/PAF^E^_e _if γ = γ_maxge _(Table 4), it is of interest to identify the area of the solution space with γ_maxge _< 1/2. Maximum utility is then obtained when γ = γ_maxge _(where PI = 1 and U_ge _= 1-γ_maxge_). For the condition

γmax⁡ge<1/2⇒pgDZ>pgxDZ     (68)
 MathType@MTEF@5@5@+=feaafiart1ev1aaatCvAUfKttLearuWrP9MDH5MBPbIqV92AaeXatLxBI9gBaebbnrfifHhDYfgasaacH8akY=wiFfYdH8Gipec8Eeeu0xXdbba9frFj0=OqFfea0dXdd9vqai=hGuQ8kuc9pgc9s8qqaq=dirpe0xb9q8qiLsFr0=vr0=vr0dc8meaabaqaciaacaGaaeqabaqabeGadaaakeaaiiGacqWFZoWzdaWgaaWcbaGagiyBa0MaeiyyaeMaeiiEaGNaem4zaCMaemyzaugabeaakiabgYda8iabigdaXiabc+caViabikdaYiabgkDiElabdchaWnaaDaaaleaacqWGNbWzaeaacqWGebarcqWGAbGwaaGccqGH+aGpcqWGWbaCdaqhaaWcbaGaem4zaCMaemiEaGhabaGaemiraqKaemOwaOfaaOGaaCzcaiaaxMaadaqadaqaaiabiAda2iabiIda4aGaayjkaiaawMcaaaaa@4D59@

where p^DZ^_gx _is given by:

RMD(1−cSD)(pgxDZ)2+[(1−cSD)(RMD−1)−(2cMD−1)(1−RSD)]pgxDZ−(RSD−cSD)=0     (69)
 MathType@MTEF@5@5@+=feaafiart1ev1aaatCvAUfKttLearuWrP9MDH5MBPbIqV92AaeXatLxBI9gBaebbnrfifHhDYfgasaacH8akY=wiFfYdH8Gipec8Eeeu0xXdbba9frFj0=OqFfea0dXdd9vqai=hGuQ8kuc9pgc9s8qqaq=dirpe0xb9q8qiLsFr0=vr0=vr0dc8meaabaqaciaacaGaaeqabaqabeGadaaakeaacqWGsbGudaWgaaWcbaGaemyta0KaemiraqeabeaakiabcIcaOiabigdaXiabgkHiTiabdogaJnaaBaaaleaacqWGtbWucqWGebaraeqaaOGaeiykaKIaeiikaGIaemiCaa3aa0baaSqaaiabdEgaNjabdIha4bqaaiabdseaejabdQfaAbaakiabcMcaPmaaCaaaleqabaGaeGOmaidaaOGaey4kaSYaamWaaeaacqGGOaakcqaIXaqmcqGHsislcqWGJbWydaWgaaWcbaGaem4uamLaemiraqeabeaakiabcMcaPiabcIcaOiabdkfasnaaBaaaleaacqWGnbqtcqWGebaraeqaaOGaeyOeI0IaeGymaeJaeiykaKIaeyOeI0IaeiikaGIaeGOmaiJaem4yam2aaSbaaSqaaiabd2eanjabdseaebqabaGccqGHsislcqaIXaqmcqGGPaqkcqGGOaakcqaIXaqmcqGHsislcqWGsbGudaWgaaWcbaGaem4uamLaemiraqeabeaakiabcMcaPaGaay5waiaaw2faaiabdchaWnaaDaaaleaacqWGNbWzcqWG4baEaeaacqWGebarcqWGAbGwaaGccqGHsislcqGGOaakcqWGsbGudaWgaaWcbaGaem4uamLaemiraqeabeaakiabgkHiTiabdogaJnaaBaaaleaacqWGtbWucqWGebaraeqaaOGaeiykaKIaeyypa0JaeGimaaJaaCzcaiaaxMaadaqadaqaaiabiAda2iabiMda5aGaayjkaiaawMcaaaaa@7B44@

solving for p^DZ^_gx _allows the region of the solution space where γ_maxge _< 1/2 to be defined.

#### 7. Cases where the 'equal environments' assumption holds (c_MD _= 1)

In the special case where the 'equal environments' assumption holds (c_MD _= 1, and hence p^DZ^_gtop _= 1/R_MD_), Equation (63) simplifies to give R_MD _≥ 2. Equation (62) also simplifies to give:

pgDZ≤1/2⇒cSD≥c1     (70)
 MathType@MTEF@5@5@+=feaafiart1ev1aaatCvAUfKttLearuWrP9MDH5MBPbIqV92AaeXatLxBI9gBaebbnrfifHhDYfgasaacH8akY=wiFfYdH8Gipec8Eeeu0xXdbba9frFj0=OqFfea0dXdd9vqai=hGuQ8kuc9pgc9s8qqaq=dirpe0xb9q8qiLsFr0=vr0=vr0dc8meaabaqaciaacaGaaeqabaqabeGadaaakeaacqWGWbaCdaqhaaWcbaGaem4zaCgabaGaemiraqKaemOwaOfaaOGaeyizIm6aaSGbaeaacqaIXaqmaeaacqaIYaGmaaGaeyO0H4Taem4yam2aaSbaaSqaaiabdofatjabdseaebqabaGccqGHLjYScqWGJbWydaWgaaWcbaGaeGymaedabeaakiaaxMaacaWLjaWaaeWaaeaacqaI3aWncqaIWaamaiaawIcacaGLPaaaaaa@44B2@

where

c1=1−(1−RSD)[1+fge2(λDZ−1)(2−RMD)](2−RMD)[1+fge2(λDZ−1)]     (71)
 MathType@MTEF@5@5@+=feaafiart1ev1aaatCvAUfKttLearuWrP9MDH5MBPbIqV92AaeXatLxBI9gBaebbnrfifHhDYfgasaacH8akY=wiFfYdH8Gipec8Eeeu0xXdbba9frFj0=OqFfea0dXdd9vqai=hGuQ8kuc9pgc9s8qqaq=dirpe0xb9q8qiLsFr0=vr0=vr0dc8meaabaqaciaacaGaaeqabaqabeGadaaakeaacqWGJbWydaWgaaWcbaGaeGymaedabeaakiabg2da9iabigdaXiabgkHiTmaalaaabaGaeiikaGIaeGymaeJaeyOeI0IaemOuai1aaSbaaSqaaiabdofatjabdseaebqabaGccqGGPaqkdaWadaqaaiabigdaXiabgUcaRiabdAgaMnaaDaaaleaacqWGNbWzcqWGLbqzaeaacqaIYaGmaaGccqGGOaakiiGacqWF7oaBdaWgaaWcbaGaemiraqKaemOwaOfabeaakiabgkHiTiabigdaXiabcMcaPiabcIcaOiabikdaYiabgkHiTiabdkfasnaaBaaaleaacqWGnbqtcqWGebaraeqaaOGaeiykaKcacaGLBbGaayzxaaaabaGaeiikaGIaeGOmaiJaeyOeI0IaemOuai1aaSbaaSqaaiabd2eanjabdseaebqabaGccqGGPaqkdaWadaqaaiabigdaXiabgUcaRiabdAgaMnaaDaaaleaacqWGNbWzcqWGLbqzaeaacqaIYaGmaaGccqGGOaakcqWF7oaBdaWgaaWcbaGaemiraqKaemOwaOfabeaakiabgkHiTiabigdaXiabcMcaPaGaay5waiaaw2faaaaacaWLjaGaaCzcamaabmaabaGaeG4naCJaeGymaedacaGLOaGaayzkaaaaaa@6DB7@

Meeting the condition p^DZ^_g _≤ 1/2 at c_SD _= 0 then requires:

RMD≥2−(1−RSD)[1+fge2(λDZ−1)RSD]     (72).
 MathType@MTEF@5@5@+=feaafiart1ev1aaatCvAUfKttLearuWrP9MDH5MBPbIqV92AaeXatLxBI9gBaebbnrfifHhDYfgasaacH8akY=wiFfYdH8Gipec8Eeeu0xXdbba9frFj0=OqFfea0dXdd9vqai=hGuQ8kuc9pgc9s8qqaq=dirpe0xb9q8qiLsFr0=vr0=vr0dc8meaabaqaciaacaGaaeqabaqabeGadaaakeaacqWGsbGudaWgaaWcbaGaemyta0KaemiraqeabeaakiabgwMiZkabikdaYiabgkHiTmaalaaabaGaeiikaGIaeGymaeJaeyOeI0IaemOuai1aaSbaaSqaaiabdofatjabdseaebqabaGccqGGPaqkaeaadaWadaqaaiabigdaXiabgUcaRiabdAgaMnaaDaaaleaacqWGNbWzcqWGLbqzaeaacqaIYaGmaaGccqGGOaakiiGacqWF7oaBdaWgaaWcbaGaemiraqKaemOwaOfabeaakiabgkHiTiabigdaXiabcMcaPiabdkfasnaaBaaaleaacqWGtbWucqWGebaraeqaaaGccaGLBbGaayzxaaaaaiaaxMaacaWLjaWaaeWaaeaacqaI3aWncqaIYaGmaiaawIcacaGLPaaacqGGUaGlaaa@5526@

It follows that if c_MD _= 1, solutions with p^DZ^_g _= 1/2 (an additive genetic model) and positive utility exist only when the following condition holds for R_MD_:

RMD≤2−(1−RSD)[1+(λDZ−1)RSD/(PAFeE)2]     (73).
 MathType@MTEF@5@5@+=feaafiart1ev1aaatCvAUfKttLearuWrP9MDH5MBPbIqV92AaeXatLxBI9gBaebbnrfifHhDYfgasaacH8akY=wiFfYdH8Gipec8Eeeu0xXdbba9frFj0=OqFfea0dXdd9vqai=hGuQ8kuc9pgc9s8qqaq=dirpe0xb9q8qiLsFr0=vr0=vr0dc8meaabaqaciaacaGaaeqabaqabeGadaaakeaacqWGsbGudaWgaaWcbaGaemyta0KaemiraqeabeaakiabgsMiJkabikdaYiabgkHiTmaalaaabaGaeiikaGIaeGymaeJaeyOeI0IaemOuai1aaSbaaSqaaiabdofatjabdseaebqabaGccqGGPaqkaeaadaWadaqaaiabigdaXiabgUcaRiabcIcaOGGaciab=T7aSnaaBaaaleaacqWGebarcqWGAbGwaeqaaOGaeyOeI0IaeGymaeJaeiykaKIaemOuai1aaSbaaSqaaiabdofatjabdseaebqabaGccqGGVaWlcqGGOaakcqWGqbaucqWGbbqqcqWGgbGrdaqhaaWcbaGaemyzaugabaGaemyraueaaOGaeiykaKYaaWbaaSqabeaacqaIYaGmaaaakiaawUfacaGLDbaaaaGaaCzcaiaaxMaadaqadaqaaiabiEda3iabiodaZaGaayjkaiaawMcaaiabc6caUaaa@5996@

Further, all three classical twin study assumptions (c_MD _= 1, p^DZ^_g _= 1/2 and f_ge _= 0) can be met only for values of R_MD _that are low enough to satisfy:

1 + *R*_*SD *_≥ *R*_*MD *_> 1     (74).

If R_MD _lies within this range, the classical twin study gives one possible solution; however, other solutions also exist. All alternative solutions favour a less 'genetic' and more 'environmental' explanation for shared sibling risks (i.e. they have higher values of c_SD_). If R_MD _is greater than 1+R_SD_, all three assumptions of the classical twin study cannot be met simultaneously.

### Comparison with the classical twins approach

Table 6 summarizes the differences between the classical twin studies approach and the method adopted here.

**Table 6:** Comparison with classical twin study

	**Classical twin study**	**Twins + siblings model**
**Genetic model**	Additive and dominance terms only: V^DZ^_g _= 1/2V_A_+1/4V_D_	Variable: V^DZ^_g _= p^DZ^_g_V_g _with 0 < = p^DZ^_g _< = 1/2

**Shared twin environments**	Equal environments assumption: c_MD _= 1	Variable: 1 < = c_MD _< = R_MD _c_MD _= R_MD _implies V_g _= 0

**Shared sibling environments**	Siblings not included.	Variable: 0 < = c_SD _< = R_SD _Familial aggregation may be due to genes (c_SD _= 0) or environment (c_SD _= R_SD_).

**Gene-environment interactions**	None	Variable: V_ge _= f^2^_ge_· V_g_· V_e_/r^2^_t _-ε/(1-ε)PAF_e _< = f_ge _< = 1/PAF_e_

**Gene-environment correlations**	None	None

**Method**	Total phenoptypic variance given by: V_P _= V_g_+V_e _V_P _is input and a single solution for V_e _and V_g _calculated. Heritabilities are given by: H^2 ^= V_g_/V_P _h^2 ^= V_A_/V_P_	V_e _and ε are input and V_g _and V_ge _calculated, for a chosen c_MD _and all possible values of f_ge _and p^DZ^_g_. Method is not valid if R_SD _= 1.

A central feature of the model is that it abandons Fisher's assumption [26] that genes act as risk factors for common traits in a manner necessarily dominated by an additive polygenic term. In his historic 1918 paper, Fisher synthesized Mendelian inheritance with Darwin's theory of evolution by showing that the genetic variance of a continuous trait could be decomposed into additive and non-additive components [26,27]. Following Fisher, the classical twin study analysis depends on writing the genetic component of a trait as a convergent series of terms, consisting of an additive term (the sum of contributions of individual alleles at each locus) plus a smaller dominance term (the sum of contributions from pairs of alleles at each locus) and – usually neglected – epistatic terms (involving potentially multiple interactions between alleles at multiple loci) [15]. Often the additive term is assumed to dominate the series (equivalent to assuming p^DZ^_g _= 1/2).

Fisher saw his polygenic model as "*abandon *[ing] *the strictly Mendelian mode of inheritance, and treat *[ing] *Galton's 'particulate inheritance' in almost its full generality*" [26]. However, it can be argued that Fisher's model is flawed in so far as it fails to distinguish between the function of alleles and the properties of traits [4,28]. In particular, epistasis (although referred to here as 'gene-gene interaction') is not strictly an interaction between genes, but can be shown to depend on the structure and interdependence of metabolic pathways [28].

The alternative model adopted here is based on correlations in *risk categories *for a trait (which may be either environmental or genetic, or both), rather than single or multiple genetic variants. Adopting Porteous' critique [28], there is no *a priori *biological reason why the parameter p^DZ^_g _(the probability that the genotypic risk category of a dizygotic twin pair is identical by descent) cannot take any value between 1/2 (its value if the additive model holds) and zero. Low p^DZ^_g _can then be understood to mean either a situation in which Fisher's polygenic model [26] is dominated by negative (synergistic) epistatic terms (for example, p^DZ^_g _= 1/2^n ^implies that interactions between n deleterious alleles are necessary to produce a phenotypic effect), or, more meaningfully, a situation in which human phenotypes are *biologically robust *to individual genetic variants [29]. Thus, in the extreme case where numerous genetic variants combine to influence a trait through the interdependence of metabolic pathways, the trait may be highly correlated in monozygotic twins (who share all the genetic variants) but not correlated at all (p^DZ^_g _= 0) in dizygotic twins or siblings (who share only half the relevant variants by descent). Although p^DZ^_g _= 0 may not be realistic, low values of p^DZ^_g _are plausible, and may even be typical of complex diseases.

The classical twin study assumptions (see above) allow a single solution to be calculated from the under-determined system of simultaneous Equations (29), (30) and (31). However, in the absence of prior knowledge about the form of the genetic model, the presence or absence of gene-environment interactions, and the validity of the 'equal environments' assumption, the approach adopted here is more rigorous.

## Results

### General model solutions

First consider the behaviour of the model when the 'equal environments' assumption holds and hence c_MD _= 1 (as described above).

**Figure 2:**
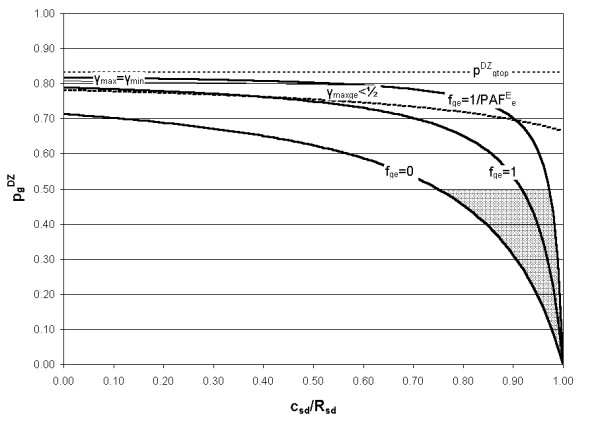
**Example model solution space with R_MD _< 1+R_SD _and U_ge _≥ 0**. Input parameters: λ_MZ _= 3.4, λ_DZ _= 3, λ_sib _= 2, ε = 0.2, PAF^E^_e _= 0.5, c_MD _= 1, r_t _= 0.1. Hence R_MD _= 1.2, R_SD _= 0.5.

**Figure 3:**
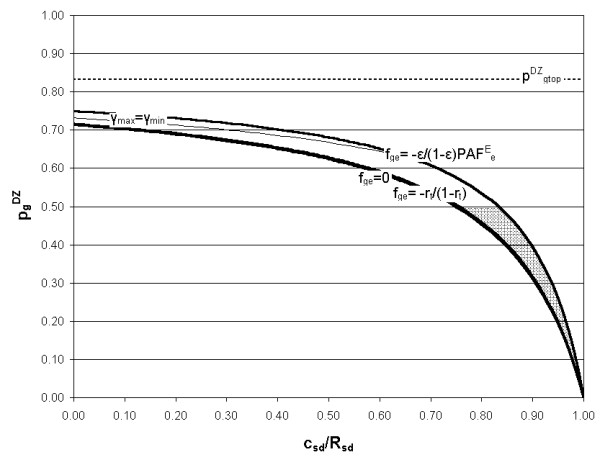
**Example model solution space with R_MD _< 1+R_SD _and U_ge _≤ 0**. Input parameters as for Figure 2.

**Figure 4:**
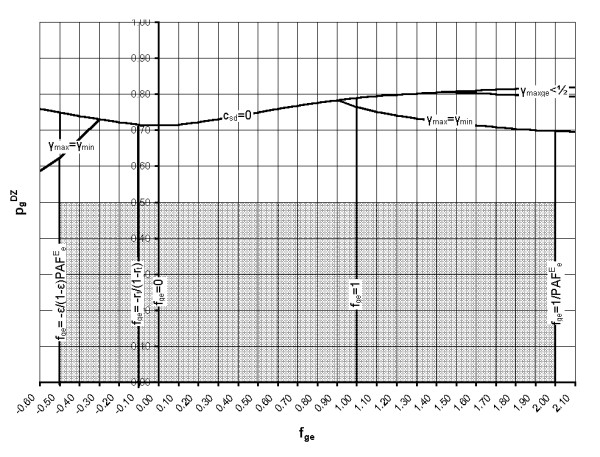
**Example full model solution space with R_MD _< 1+R_SD_**. Input parameters as for Figure 2, with the solution space transformed so that f_ge _is on the horizontal axis.

Figures 2, 3 and 4 show the possible solution spaces for an arbitrary set of plausible input parameters satisfying the requirement R_MD _> 1+R_SD _necessary for the classical twin study solution to exist. In Figure 2 the gene-environment interaction factor f_ge _and hence utility, U_ge_, are both positive and in Figure 3 they are negative. The horizontal axis shows c_SD_/R_SD_, which is zero if shared sibling risk is due to shared genetic factors only and 1 if shared sibling risk is due to shared environmental factors only. The vertical axis shows p^DZ^_g_, which is 1/2 if the additive genetic model holds, but may reduce to zero if epistasis dominates and the phenotype is robust to genetic variation. The three curved solid lines represent three models of gene-environment (G-E) interaction: an additive G-E model (i.e. no gene-environment interaction, f_ge _= 0); a multiplicative G-E model (f_ge _= 1); and maximum G-E interaction (f_ge _= 1/PAF^E^_e_). The possible solution spaces are shaded grey. Each point in each shaded solution space corresponds to a given genetic model (defined by p^DZ^_g_) and a given G-E interaction model (defined by f_ge_). Figure 4 plots the entire solution space (including both negative and positive utility) by transforming the horizontal axis to represent the G-E interaction parameter, f_ge_. Although the classical twin model can fit the data, an infinite number of other solutions corresponding to different genetic and gene-environment interaction models also exist. In this example, the line γ_max _= γ_min _lies outside the solution space and no solutions exist with γ_maxge _< 1/2.

For lower values of R_MD_, the curves defining the solution space are shifted downwards [see Additional files 2 to 9], so that the line f_ge _= 0 (corresponding to no gene-environment interaction) lies entirely below the line p^DZ^_g _= 1/2 (corresponding to an additive genetic model). The classical twin study solution does not exist, but many other combinations of genetic and gene-environment interaction models may fit the data.

When c_MD _> 1, lines of constant f_ge _no longer decrease monotonically to zero, and are also shifted upwards, so that solutions with strong G-E interactions are no longer possible [see Additional files 10 to 12].

### Example applications using twin, sibling and environmental data

#### Input values

Consider example applications of the model for male lung cancer, female breast cancer and schizophrenia. The model input variables used are shown in Table 7.

The recurrence risks, λ, and total risks, r_t_, for breast and lung cancer are those calculated by Risch [30], based on Scandinavian twin data reported by Lichtenstein et al. [31] (involving more than 44,000 twin pairs) and Swedish familial data reported by Doug and Hemminki [32] (involving more than 2 million families). The proportion of the population exposed, ε, and population attributable fraction, PAF^E^_e_, for breast cancer are taken from those reported by Rockhill et al. [33] for a US population. Although strictly speaking these values may not be appropriate for a Scandinavian population, and include a component due to family history that may be (at least partly) genetic, they give a low V_e_, consistent with the known environmental risk factors for breast cancer, and results are not sensitive to these input values (because V_e _is so small). For lung cancer, it is assumed that 15% of the Scandinavian population smokes and that 86% of lung cancer cases could be avoided if they did not (giving a risk of lung cancer in smokers of 10%).

The recurrence risks λ, and total risk, r_t_, for schizophrenia are those used by Risch [16], based on European data summarized by McGue et al. [34]. More recent twin studies for schizophrenia have given variable results and this example should be treated as illustrative only. Further, environmental exposures and population attributable fractions are unknown for schizophrenia. Two exploratory sets of results are therefore reported, using data consistent with a low environmental variance (based on the values used for breast cancer), and high environmental variance (based on the values used for smoking and lung cancer).

Detailed results for the three diseases are shown in [Additional file 13, 14, 15, 16, 17, 18, 19, 20, 21, 22, 23, 24, 25, 26, 27, 28, 29, 30, 31, 32, 33]. The key findings are outlined below.

#### Breast cancer results

For breast cancer, the PAF^E^_e _associated with known environmental factors is low. The value of the model is therefore less in calculating the utility of targeted environmental interventions than in exploring the solution space for a complex disease with R_MD _close to 2.

Although strictly speaking the classical twin study solution (with an additive genetic model, p^DZ^_g _= 1/2, and an additive G-E model, f_ge _= 0) does not exist as a solution, it might lie within the margin of error of the data. However, an infinite number of other models also could also fit the data. The classical twin model result always overestimates the genetic component of the variance, which reduces as the gene-environment interaction factor f_ge _increases, and also as p^DZ^_g _decreases (i.e. as epistatic terms begin to dominate the genetic model). These alternative models imply that shared environmental factors may partially explain familial aggregation of breast cancer. This contrasts with the classical twin method result (see earlier), which for R_MD _= 2 leads to the inevitable conclusion that shared sibling risk must be due solely to shared genes [35].

In theory, a model with p^DZ^_g _= 0 (where shared sibling risk is due entirely to shared environmental factors) could fit the data. However, for breast cancer the existence of known mutations that significantly increase risk (particularly mutations in the BRCA1 and BRCA2 genes, which are relatively common) rules out this solution. Although it is not possible to subtract out the effect of these mutations from the model, it is possible to show that they could be sufficient to explain the twin data if a G-E interaction also exists. For example, one possible solution consistent with the data could involve one or more dominant genes (p^DZ^_g _= 1/2), a strong G-E interaction (f_ge _= 1/PAF^E ^_e_), but a largely environmental explanation for shared sibling risk (say c_SD_/R_SD _= 0.9). This solution implies that the genetic component of the variance is less than a fifth of the classical twin study result, which could be low enough to be explained by mutations in the BRCA1 and BRCA2 genes alone [35]. If this model were correct it would have important implications for women with such mutations, but would not contribute significantly to reducing the incidence of breast cancer in the population as a whole, because the affected proportion of the population γ would be rather small. Other solutions, involving different genetic models with lower p^DZ^_g_, and/or less gene-environment interaction, are also possible.

The line γ_max _= γ_min _does not occur within the solution space for breast cancer; however, in some circumstances the lines γ_max _and γ_min _may be rather close together. This suggests that, although as expected there is always a trade-off between selecting a small proportion (γ_min_) of the population with a high Positive Predictive Value (PPV), or a larger proportion of the population (γ_max_) with a higher Population Impact (PI) [19], some possible solutions could exist for breast cancer where the PPV and PI are both relatively high. Further, γ_max _is often less than 1/2, so that, in these regions of the possible solution space, maximum utility might be obtained by targeting less than 50% of the population. However, known environmental factors for breast cancer are often not amenable to intervention and other possible solutions, with low, zero or negative utility, also exist.

#### Lung cancer results

For lung cancer, all the possible solutions imply that shared sibling risk is largely due to shared environmental factors (smoking) because solutions occur only when c_SD_/R_SD _is close to 1. Unlike for breast cancer, the line γ_max _= γ_min _lies outside the solution space, even for negative f_ge_, as does the area of solutions with γ_maxge _< 1/2. However, the classical twin study solution, with f_ge _= 0 and p^DZ^_g _= 1/2, clearly lies within the solution space.

Although the classical twin model again provides an upper limit to the genetic component of the variance, even the classical result indicates that the risk of lung cancer is dominated by smoking in this population and the variance has at most a small genetic component.

Unlike the breast cancer example, γ_max _and γ_min _are always far apart, suggesting a strong trade off between high Postive Predictive Value (R_ge_) for a genotypic test and a high Population Impact (PI) for a targeted intervention. This means that a genotypic test that predicts which smokers will get lung cancer cannot exist. To predict all cases of lung cancer in smokers (i.e. to obtain PI = 1), 95% or more of the population would have to be in the high genotypic risk group, and the predictive value of such a test would be very low.

Because the genetic component of the variance is so small, it follows that the utility of genetic 'prediction and prevention' (measured by U_ge_) is also small (from Equation (28)). Utility is maximum when γ = 1/2, but even then values are low. The maximum utility of genotyping occurs when about 60% of cases could be prevented by targeting the 50% of smokers at high genotypic risk. However, other possible solutions have zero or negative utility.

#### Schizophrenia results

For schizophrenia, the classical twin study solution (with f_ge _= 0 and p^DZ^_g _= 1/2 and c_MD _= 1) cannot not fit the data. If the 'equal environments' assumption holds, neither a single dominant gene (p^DZ^_g _= 1/2), nor additive polygenic model (also with p^DZ^_g _= 1/2), nor single recessive gene (p^DZ^_g _= 1/4) can explain the twin and family data, consistent with Risch's 1990 findings [16]. This may suggest that the genetic model for schizophrenia is likely to be dominated by epistatic terms. However, if gene-environment interactions are important, it is also possible that a recessive gene, combined with at least multiplicative G-E interaction (p^DZ^_g _= 1/4 and f_ge _= 1 or higher), could explain the data.

The possible solution spaces include purely genetic explanations for shared sibling risk (at c_SD_/R_SD _= 0), or purely environmental ones (at c_SD_/R_SD _= 1, applicable if p^DZ^_g _= 0).

Assuming a small environmental component to the variance, there is no region of the solution space for which γ_maxge _< 1/2, suggesting that the utility of targeted environmental interventions under these assumptions is likely to be low. However, if the environmental component of the variance is assumed to be much larger, the available solution space changes dramatically, because the line γ_max _= γ_min _now constrains the solution space to a much smaller area, which excludes solutions with no G-E interaction (f_ge _= 0). Special solutions may exist along the line γ_max _= γ_min_, as shown in Table 5. Because the environmental factors contributing to schizophrenia are unknown, it is impossible to draw any conclusions about the potential benefits of targeting environmental interventions at those at high genotypic risk.

Because prenatal development is thought to be important in schizophrenia, it is plausible that monozygotic twins are more likely to share environmental risk factors than dizygotic twins are. Breaking the 'equal environments' assumption changes the shape of the solution space significantly, and, assuming a small environmental component to the variance, only limited G-E interactions are now possible (the multiplicative G-E model, f_ge _= 1, lies largely outside the solution space). The utility of targeting environmental interventions by genotype is then likely to be low. However, in these circumstances it is possible that an additive genetic model (p^DZ^_g _= 1/2) with some G-E interaction, or a recessive gene (p^DZ^_g _= 1/4) with no G-E interaction, could explain the data.

## Discussion

If Fisher's polygenic model [26] is abandoned, along with the usual twin study assumption that there are no gene-environment interactions, the four-category model developed by Khoury and others can be combined with twin, family and environmental data to implement a 'top down' approach to assessing the utility of targeting environmental/lifestyle interventions by genotype. Scoping studies, valid when R_SD _≠ 1, provide a first step to modelling the health of populations [23].

Abandoning Fisher's assumption that the polygenic model is necessarily dominated by an additive term can be justified by the growing evidence that phenotypic effects can result from the synergistic action of alleles in many genes [36]. For example, Bardet-Biedl Syndrome, historically assumed to be a recessive trait, has been shown to involve three interacting mutations at two loci in some patients (implying that p^DZ^_g _= 1/8) and, more recently, an additional locus has been identified that can also interact to change disease severity and symptoms [37]. Both positive and negative gene-environment interactions have also been observed in human diseases, although there are difficulties in confirming their statistical validity [38,39].

The model also allows the impact of the much criticised 'equal environments' assumption to be explored.

A number of conclusions can be drawn about the merits of the classical twin study and the utility of genetic 'prediction and prevention'.

Firstly, the model confirms that the classical twin study solution is not always valid and gives at best an upper limit to the genetic component of the variance of a trait. The importance of the 'equal environments' assumption and of gene-environment interactions have previously been recognised [17,18]; however, less attention has been paid to the potential role of gene-gene interactions (epistasis). For larger values of R_MD _(greater than 1+R_SD_), observed for conditions such as schizophrenia, the model generalizes Risch's findings [16] to show that the three assumptions of the classical twin model cannot all be satisfied simultaneously. For intermediate R_MD _values, observed for conditions such as breast cancer (for which R_MD _is approximately 2), the model illustrates that the conclusion drawn from classical twin studies, that familial aggregation is due entirely to shared genetic factors, may be erroneous. This raises the possibility – previously rejected on the basis of twin study results [35] – that genetic variants are important in determining risk only for the relatively rare familial forms of cancer. If so, genetic models of familial aggregation (for example [40]) may be incorrect and the hunt for additional susceptibility genes could be largely fruitless. Existing published findings might then reflect prevailing bias, rather than true associations [14].

Secondly, the model confirms that the potential for reducing the incidence of common diseases using environmental/lifestyle interventions targeted by genotype may be limited [7] by:

(i) the low importance of genetic differences in determining the risk of some conditions (for example, lung cancer);

(ii) the complexity of gene-gene and gene-environment interactions and/or lack of knowledge of environmental factors (for example, schizophrenia).

Targeting environmental/lifestyle interventions at those at 'high genotypic risk' can be of high utility only in specific circumstances. The utility of targeting environmental interactions by genotype (compared to randomly selecting the same number of people from the population) is zero if there is no gene-environment interaction. Utility can also be negative in the presence of a negative interaction (i.e. if the people at high genotypic risk have *less to gain *by the intervention than people at low genotypic risk). The finding that utility increases with gene-environment interaction is consistent with Khoury and Wagener [19] but the relationship is considerably clarified by the adoption here of different measures of the population attributable fraction associated with a targeted intervention (PAF^E^_ge_) and of utility (U_ge_). Further, by formally introducing constraints on the model (for example, that risks are positive and do not exceed 100%), it is possible to demonstrate that both the gene-environment interaction factor and utility have maximum values, which cannot be exceeded for a given data set.

The lung cancer example is apparently trivial but also of critical importance. The R_MD _value for lung cancer is close to 1, and neither the Scandinavian data used here [31], nor earlier US studies [41], have identified a significant heritable component. It follows from Equation (27) that if the genetic component of the variance, V_g_, is zero, V_ge _(the G-E component of the variance) is also zero and using genotyping to target an intervention such as smoking cessation is therefore of zero utility (no better than randomly selecting the same number of individuals). This approximate conclusion is confirmed by the results presented for lung cancer, which show extremely low utility. The detailed calculations may at first sight seem unnecessary, particularly because smoking causes multiple diseases and targeting smoking cessation on the basis of lung cancer risk alone is therefore ill-advised. However, the idea that a genetic test will one day predict which smokers get lung cancer has been widely promoted in the literature and has driven much research aimed at identifying the supposed 'genes for lung cancer' [42]. The results presented here strongly suggest that there will never be a genetic or genotypic test that predicts which smokers will get lung cancer, because the genetic component of the variance is not high enough.

Finally, the model illustrates the argument of Terwilliger and Weiss [11] that the potential for population biobanks to quantify risks for complex disease is limited by a 'multiple testing' problem caused by the large number of genetic and gene-environment interaction models that could fit existing data. Each point in each solution space described above represents a different combination of a genetic risk model (defined by p^DZ^_g_) and a G-E interaction model (defined by f_ge_). Further, any given value of p^DZ^_g _may be obtained by an infinite number of different combinations of different alleles acting through multiple biological pathways. Because the number of hypotheses that could be tested is essentially infinite, sample sizes necessary to quantify the risks (R_oo_, R_go_, R_oe _and R_ge_) could *"plausibly be larger than the number of people that have ever lived*" [11].

The model has several limitations. Measurements of shared sibling risk (λ_sib_) are needed from the same population as twin data, and the scoping studies are only valid for λ_DZ _> λ_sib_, implying that environmental risks are more strongly correlated in dizygotic twins than other siblings. Some data exist to support this assumption for smoking [43] but for other exposures its validity is usually unknown. However, the model does not reduce to the classical twin study solution if this condition is not met: instead, data from more relatives are needed. In principle the model could, and should, be expanded to include data from more relatives, other data (such as migration study data), more risk categories and error terms. However, the number of unknown parameters will then increase, unless more data are available to quantify exposures (which change from generation to generation) and to estimate the extent to which environments are correlated between different types of relative.

Treating exposure and environmental variance (or population attributable fraction) as input data is also problematic when the effects of environmental factors on risk are often unknown. Further, the simple nature of the model (with one environmental axis) cannot adequately represent the complexity of environmental (including socio-economic) causes of disease. However, if targeting environmental interventions by genotype is to be considered, this implies that at least something is known (or expected to be learned) about environmental factors, such as particular exposures, that are amenable to intervention.

The assumption of no gene-environment correlation will often hold (for example it is rather implausible that the same genes strongly influence both lung cancer risk and nicotine addiction), but is not necessarily always true. Adult lactose intolerance is an example of a condition with a strong gene-environment interaction where targeted intervention to avoid drinking milk may be of high utility. However, the model is invalid for lactose intolerance unless exposures are applied equally to the population studied because, in general, people who are lactose intolerant may be less likely to drink milk (a gene-environment correlation) owing to the unpleasant symptoms.

A more fundamental problem is caused by the assumptions that: (i) the risks R_oo_, R_go_, R_oe _and R_ge _are inherent properties of a given trait within a given population (with a given γ and ε) and that there are therefore no confounders; and (ii) risks are randomly distributed within these categories.

These assumptions, although often made, are implausible in many situations. The assumption of no confounders means that the model can only represent a subset of the potential models of gene-gene and gene-environment interaction described by more complex models (for example [17]). It is unlikely to be met if multiple genetic factors interact with multiple environmental ones [44]. Although this may well render the results presented here invalid, such complexity is likely to reduce the utility of targeting by genotype, rather than enhance it. Hence, situations where the 'no confounders' assumption at least approximately holds are those most likely to be of relevance to public health.

The second assumption neglects the fact that for most exposures there is a gradient in risk, with higher exposure meaning higher risk, and that the same may also be true of genetic factors. This means that increasing the number of categories in the model will increase V_e _(see [45]) and perhaps V_g_. Further, these subcategories may be differently correlated between relatives (for example, the twin of a heavy smoker may be more likely to be a heavy smoker than a light one). If so, a relative of a proband may not be representative of their allocated risk category in the four-category model and Equation (22) then becomes invalid.

More broadly, these assumptions make the model, like the classical twin model, essentially deterministic: it assumes that all the factors contributing to correlations in risk between relatives are perfectly known and are either environmental or genetic. Retention of these assumptions here may be problematic and could limit the applicability of the results. Nevertheless, all the other questionable assumptions of the classical twin model have been simultaneously removed.

## Conclusion

The model shows that the potential for reducing the incidence of common diseases using environmental interventions targeted by genotype may be limited, except in special cases. The model also confirms that the importance of an individual's genotype in determining their risk of complex diseases tends to be exaggerated by the classical twin studies method, owing to the 'equal environments' assumption and the assumption of no gene-environment interaction. In addition, if phenotypes are genetically robust, because of epistasis, a largely environmental explanation for shared sibling risk is plausible, even if the classical heritability is high. The model therefore highlights the possibility – previously rejected on the basis of twin study results – that inherited genetic variants are important in determining risk only for the relatively rare familial forms of diseases such as breast cancer. If so, genetic models of familial aggregation may be incorrect and the hunt for additional susceptibility genes could be largely fruitless.

## Competing interests

The author(s) declare that they have no competing interests.

## Appendix A: formal derivation of equation (31)

Equation (23) may be derived more formally by extending the matrix method of Li and Sacks [46].

Define the probability that an affected proband is in genotypic risk category z and environmental risk category w as P_zw _and assume that risks are randomly distributed within categories. Using the definitions of the four category model given in Table 1, a vector **P **may be defined:

P=(PooPoePgoPge)=((1−ε)(1−γ)Roo/rtε(1−γ)Roe/rtγ(1−ε)Rgo/rtγεRge/rt)     (A1)
 MathType@MTEF@5@5@+=feaafiart1ev1aaatCvAUfKttLearuWrP9MDH5MBPbIqV92AaeXatLxBI9gBaebbnrfifHhDYfgasaacH8akY=wiFfYdH8Gipec8Eeeu0xXdbba9frFj0=OqFfea0dXdd9vqai=hGuQ8kuc9pgc9s8qqaq=dirpe0xb9q8qiLsFr0=vr0=vr0dc8meaabaqaciaacaGaaeqabaqabeGadaaakeaacqWHqbaucqGH9aqpdaqadaqaauaabeqaeeaaaaqaaiabdcfaqnaaBaaaleaacqWGVbWBcqWGVbWBaeqaaaGcbaGaemiuaa1aaSbaaSqaaiabd+gaVjabdwgaLbqabaaakeaacqWGqbaudaWgaaWcbaGaem4zaCMaem4Ba8gabeaaaOqaaiabdcfaqnaaBaaaleaacqWGNbWzcqWGLbqzaeqaaaaaaOGaayjkaiaawMcaaiabg2da9maabmaabaqbaeqabqqaaaaabaGaeiikaGIaeGymaeJaeyOeI0ccciGae8xTduMaeiykaKIaeiikaGIaeGymaeJaeyOeI0Iae83SdCMaeiykaKYaaSGbaeaacqWGsbGudaWgaaWcbaGaem4Ba8Maem4Ba8gabeaaaOqaaiabdkhaYnaaBaaaleaacqWG0baDaeqaaaaaaOqaaiab=v7aLjabcIcaOiabigdaXiabgkHiTiab=n7aNjabcMcaPmaalyaabaGaemOuai1aaSbaaSqaaiabd+gaVjabdwgaLbqabaaakeaacqWGYbGCdaWgaaWcbaGaemiDaqhabeaaaaaakeaacqWFZoWzcqGGOaakcqaIXaqmcqGHsislcqWF1oqzcqGGPaqkdaWcgaqaaiabdkfasnaaBaaaleaacqWGNbWzcqWGVbWBaeqaaaGcbaGaemOCai3aaSbaaSqaaiabdsha0bqabaaaaaGcbaGae83SdCMae8xTdu2aaSGbaeaacqWGsbGudaWgaaWcbaGaem4zaCMaemyzaugabeaaaOqaaiabdkhaYnaaBaaaleaacqWG0baDaeqaaaaaaaaakiaawIcacaGLPaaacaWLjaGaaCzcamaabmaabaacbaGae4xqaeKaeGymaedacaGLOaGaayzkaaaaaa@80CD@

A risk vector **R **may also be defined:

R=(RooRoeRgoRge)     (A2)
 MathType@MTEF@5@5@+=feaafiart1ev1aaatCvAUfKttLearuWrP9MDH5MBPbIqV92AaeXatLxBI9gBaebbnrfifHhDYfgasaacH8akY=wiFfYdH8Gipec8Eeeu0xXdbba9frFj0=OqFfea0dXdd9vqai=hGuQ8kuc9pgc9s8qqaq=dirpe0xb9q8qiLsFr0=vr0=vr0dc8meaabaqaciaacaGaaeqabaqabeGadaaakeaacqWHsbGucqGH9aqpdaqadaqaauaabeqaeeaaaaqaaiabdkfasnaaBaaaleaacqWGVbWBcqWGVbWBaeqaaaGcbaGaemOuai1aaSbaaSqaaiabd+gaVjabdwgaLbqabaaakeaacqWGsbGudaWgaaWcbaGaem4zaCMaem4Ba8gabeaaaOqaaiabdkfasnaaBaaaleaacqWGNbWzcqWGLbqzaeqaaaaaaOGaayjkaiaawMcaaiaaxMaacaWLjaWaaeWaaeaaieaacqWFbbqqcqaIYaGmaiaawIcacaGLPaaaaaa@45C8@

Now define G_xy _as the conditional probability P(relative is in genotypic risk category y|proband is in genotypic risk category x). Similarly, define E_xy _as the conditional probability P(relative is in environmental risk category y|proband is in environmental risk category x). Using the definitions of p^rel^_g _and p^rel^_e _given in Section 2.5, matrices **G **and **E **may be written such that:

Grel=(GooGogGgoGgg)=(pgrel+(1−γ)(1−pgrel)γ(1−pgrel)(1−γ)(1−pgrel)pgrel+γ(1−γ)pgrel)     (A3)
 MathType@MTEF@5@5@+=feaafiart1ev1aaatCvAUfKttLearuWrP9MDH5MBPbIqV92AaeXatLxBI9gBaebbnrfifHhDYfgasaacH8akY=wiFfYdH8Gipec8Eeeu0xXdbba9frFj0=OqFfea0dXdd9vqai=hGuQ8kuc9pgc9s8qqaq=dirpe0xb9q8qiLsFr0=vr0=vr0dc8meaabaqaciaacaGaaeqabaqabeGadaaakeaacqWHhbWrdaahaaWcbeqaaiabdkhaYjabdwgaLjabdYgaSbaakiabg2da9maabmaabaqbaeqabiGaaaqaaiabdEeahnaaBaaaleaacqWGVbWBcqWGVbWBaeqaaaGcbaGaem4raC0aaSbaaSqaaiabd+gaVjabdEgaNbqabaaakeaacqWGhbWrdaWgaaWcbaGaem4zaCMaem4Ba8gabeaaaOqaaiabdEeahnaaBaaaleaacqWGNbWzcqWGNbWzaeqaaaaaaOGaayjkaiaawMcaaiabg2da9maabmaabaqbaeqabiGaaaqaaiabdchaWnaaDaaaleaacqWGNbWzaeaacqWGYbGCcqWGLbqzcqWGSbaBaaGccqGHRaWkcqGGOaakcqaIXaqmcqGHsisliiGacqWFZoWzcqGGPaqkcqGGOaakcqaIXaqmcqGHsislcqWGWbaCdaqhaaWcbaGaem4zaCgabaGaemOCaiNaemyzauMaemiBaWgaaOGaeiykaKcabaGae83SdCMaeiikaGIaeGymaeJaeyOeI0IaemiCaa3aa0baaSqaaiabdEgaNbqaaiabdkhaYjabdwgaLjabdYgaSbaakiabcMcaPaqaaiabcIcaOiabigdaXiabgkHiTiab=n7aNjabcMcaPiabcIcaOiabigdaXiabgkHiTiabdchaWnaaDaaaleaacqWGNbWzaeaacqWGYbGCcqWGLbqzcqWGSbaBaaGccqGGPaqkaeaacqWGWbaCdaqhaaWcbaGaem4zaCgabaGaemOCaiNaemyzauMaemiBaWgaaOGaey4kaSIae83SdCMaeiikaGIaeGymaeJaeyOeI0Iae83SdCMaeiykaKIaemiCaa3aa0baaSqaaiabdEgaNbqaaiabdkhaYjabdwgaLjabdYgaSbaaaaaakiaawIcacaGLPaaacaWLjaGaaCzcamaabmaabaacbaGae4xqaeKaeG4mamdacaGLOaGaayzkaaaaaa@963D@

Erel=(EooEoeEeoEee)=(perel+(1−ε)(1−perel)ε(1−perel)(1−ε)(1−perel)perel+ε(1−ε)perel)     (A4)
 MathType@MTEF@5@5@+=feaafiart1ev1aaatCvAUfKttLearuWrP9MDH5MBPbIqV92AaeXatLxBI9gBaebbnrfifHhDYfgasaacH8akY=wiFfYdH8Gipec8Eeeu0xXdbba9frFj0=OqFfea0dXdd9vqai=hGuQ8kuc9pgc9s8qqaq=dirpe0xb9q8qiLsFr0=vr0=vr0dc8meaabaqaciaacaGaaeqabaqabeGadaaakeaacqWHfbqrdaahaaWcbeqaaiabdkhaYjabdwgaLjabdYgaSbaakiabg2da9maabmaabaqbaeqabiGaaaqaaiabdweafnaaBaaaleaacqWGVbWBcqWGVbWBaeqaaaGcbaGaemyrau0aaSbaaSqaaiabd+gaVjabdwgaLbqabaaakeaacqWGfbqrdaWgaaWcbaGaemyzauMaem4Ba8gabeaaaOqaaiabdweafnaaBaaaleaacqWGLbqzcqWGLbqzaeqaaaaaaOGaayjkaiaawMcaaiabg2da9maabmaabaqbaeqabiGaaaqaaiabdchaWnaaDaaaleaacqWGLbqzaeaacqWGYbGCcqWGLbqzcqWGSbaBaaGccqGHRaWkcqGGOaakcqaIXaqmcqGHsisliiGacqWF1oqzcqGGPaqkcqGGOaakcqaIXaqmcqGHsislcqWGWbaCdaqhaaWcbaGaemyzaugabaGaemOCaiNaemyzauMaemiBaWgaaOGaeiykaKcabaGae8xTduMaeiikaGIaeGymaeJaeyOeI0IaemiCaa3aa0baaSqaaiabdwgaLbqaaiabdkhaYjabdwgaLjabdYgaSbaakiabcMcaPaqaaiabcIcaOiabigdaXiabgkHiTiab=v7aLjabcMcaPiabcIcaOiabigdaXiabgkHiTiabdchaWnaaDaaaleaacqWGLbqzaeaacqWGYbGCcqWGLbqzcqWGSbaBaaGccqGGPaqkaeaacqWGWbaCdaqhaaWcbaGaemyzaugabaGaemOCaiNaemyzauMaemiBaWgaaOGaey4kaSIae8xTduMaeiikaGIaeGymaeJaeyOeI0Iae8xTduMaeiykaKIaemiCaa3aa0baaSqaaiabdwgaLbqaaiabdkhaYjabdwgaLjabdYgaSbaaaaaakiaawIcacaGLPaaacaWLjaGaaCzcamaabmaabaacbaGae4xqaeKaeGinaqdacaGLOaGaayzkaaaaaa@9603@

Finally, define X_ab-cd _as the conditional probability P(relative is in risk category cd|proband is in risk category ab), where the risk categories are as defined in Table 1 (for example risk categorgy 'ge' implies high-genotypic and high-environmental risk). Provided p^rel^_g _and p^rel^_e _are independent (there are no gene-environment correlations), the gene-environment interaction matrix **M**^rel^_ge _may be written as:

Mgerel=(Xoo−ooXoo−oeXoo−goXoo−geXoe−ooXoe−oeXoe−goXoe−geXgo−ooXgo−oeXgo−goXgo−geXge−ooXge−oeXge−goXge−ge)=(GooEooGooEoeGogEooGogEoeGooEeoGooEeeGogEeoGogEeeGgoEooGgoEoeGggEooGggEoeGgoEeoGgoEeeGggEeoGggEee)     (A5)
 MathType@MTEF@5@5@+=feaafiart1ev1aaatCvAUfKttLearuWrP9MDH5MBPbIqV92AaeXatLxBI9gBaebbnrfifHhDYfgasaacH8akY=wiFfYdH8Gipec8Eeeu0xXdbba9frFj0=OqFfea0dXdd9vqai=hGuQ8kuc9pgc9s8qqaq=dirpe0xb9q8qiLsFr0=vr0=vr0dc8meaabaqaciaacaGaaeqabaqabeGadaaakeaacqWHnbqtdaqhaaWcbaGaem4zaCMaemyzaugabaGaemOCaiNaemyzauMaemiBaWgaaOGaeyypa0ZaaeWaaeaafaqabeabeaaaaaqaaiabdIfaynaaBaaaleaacqWGVbWBcqWGVbWBcqGHsislcqWGVbWBcqWGVbWBaeqaaaGcbaGaemiwaG1aaSbaaSqaaiabd+gaVjabd+gaVjabgkHiTiabd+gaVjabdwgaLbqabaaakeaacqWGybawdaWgaaWcbaGaem4Ba8Maem4Ba8MaeyOeI0Iaem4zaCMaem4Ba8gabeaaaOqaaiabdIfaynaaBaaaleaacqWGVbWBcqWGVbWBcqGHsislcqWGNbWzcqWGLbqzaeqaaaGcbaGaemiwaG1aaSbaaSqaaiabd+gaVjabdwgaLjabgkHiTiabd+gaVjabd+gaVbqabaaakeaacqWGybawdaWgaaWcbaGaem4Ba8MaemyzauMaeyOeI0Iaem4Ba8MaemyzaugabeaaaOqaaiabdIfaynaaBaaaleaacqWGVbWBcqWGLbqzcqGHsislcqWGNbWzcqWGVbWBaeqaaaGcbaGaemiwaG1aaSbaaSqaaiabd+gaVjabdwgaLjabgkHiTiabdEgaNjabdwgaLbqabaaakeaacqWGybawdaWgaaWcbaGaem4zaCMaem4Ba8MaeyOeI0Iaem4Ba8Maem4Ba8gabeaaaOqaaiabdIfaynaaBaaaleaacqWGNbWzcqWGVbWBcqGHsislcqWGVbWBcqWGLbqzaeqaaaGcbaGaemiwaG1aaSbaaSqaaiabdEgaNjabd+gaVjabgkHiTiabdEgaNjabd+gaVbqabaaakeaacqWGybawdaWgaaWcbaGaem4zaCMaem4Ba8MaeyOeI0Iaem4zaCMaemyzaugabeaaaOqaaiabdIfaynaaBaaaleaacqWGNbWzcqWGLbqzcqGHsislcqWGVbWBcqWGVbWBaeqaaaGcbaGaemiwaG1aaSbaaSqaaiabdEgaNjabdwgaLjabgkHiTiabd+gaVjabdwgaLbqabaaakeaacqWGybawdaWgaaWcbaGaem4zaCMaemyzauMaeyOeI0Iaem4zaCMaem4Ba8gabeaaaOqaaiabdIfaynaaBaaaleaacqWGNbWzcqWGLbqzcqGHsislcqWGNbWzcqWGLbqzaeqaaaaaaOGaayjkaiaawMcaaiabg2da9maabmaabaqbaeqabqabaaaaaeaacqWGhbWrdaWgaaWcbaGaem4Ba8Maem4Ba8gabeaakiabdweafnaaBaaaleaacqWGVbWBcqWGVbWBaeqaaaGcbaGaem4raC0aaSbaaSqaaiabd+gaVjabd+gaVbqabaGccqWGfbqrdaWgaaWcbaGaem4Ba8MaemyzaugabeaaaOqaaiabdEeahnaaBaaaleaacqWGVbWBcqWGNbWzaeqaaOGaemyrau0aaSbaaSqaaiabd+gaVjabd+gaVbqabaaakeaacqWGhbWrdaWgaaWcbaGaem4Ba8Maem4zaCgabeaakiabdweafnaaBaaaleaacqWGVbWBcqWGLbqzaeqaaaGcbaGaem4raC0aaSbaaSqaaiabd+gaVjabd+gaVbqabaGccqWGfbqrdaWgaaWcbaGaemyzauMaem4Ba8gabeaaaOqaaiabdEeahnaaBaaaleaacqWGVbWBcqWGVbWBaeqaaOGaemyrau0aaSbaaSqaaiabdwgaLjabdwgaLbqabaaakeaacqWGhbWrdaWgaaWcbaGaem4Ba8Maem4zaCgabeaakiabdweafnaaBaaaleaacqWGLbqzcqWGVbWBaeqaaaGcbaGaem4raC0aaSbaaSqaaiabd+gaVjabdEgaNbqabaGccqWGfbqrdaWgaaWcbaGaemyzauMaemyzaugabeaaaOqaaiabdEeahnaaBaaaleaacqWGNbWzcqWGVbWBaeqaaOGaemyrau0aaSbaaSqaaiabd+gaVjabd+gaVbqabaaakeaacqWGhbWrdaWgaaWcbaGaem4zaCMaem4Ba8gabeaakiabdweafnaaBaaaleaacqWGVbWBcqWGLbqzaeqaaaGcbaGaem4raC0aaSbaaSqaaiabdEgaNjabdEgaNbqabaGccqWGfbqrdaWgaaWcbaGaem4Ba8Maem4Ba8gabeaaaOqaaiabdEeahnaaBaaaleaacqWGNbWzcqWGNbWzaeqaaOGaemyrau0aaSbaaSqaaiabd+gaVjabdwgaLbqabaaakeaacqWGhbWrdaWgaaWcbaGaem4zaCMaem4Ba8gabeaakiabdweafnaaBaaaleaacqWGLbqzcqWGVbWBaeqaaaGcbaGaem4raC0aaSbaaSqaaiabdEgaNjabd+gaVbqabaGccqWGfbqrdaWgaaWcbaGaemyzauMaemyzaugabeaaaOqaaiabdEeahnaaBaaaleaacqWGNbWzcqWGNbWzaeqaaOGaemyrau0aaSbaaSqaaiabdwgaLjabd+gaVbqabaaakeaacqWGhbWrdaWgaaWcbaGaem4zaCMaem4zaCgabeaakiabdweafnaaBaaaleaacqWGLbqzcqWGLbqzaeqaaaaaaOGaayjkaiaawMcaaiaaxMaacaWLjaWaaeWaaeaaieaacqWFbbqqcqaI1aqnaiaawIcacaGLPaaaaaa@3D28@

Then the risk in a relative of the proband is given by:

λrelrt=P.(MgerelR)     (A6)
 MathType@MTEF@5@5@+=feaafiart1ev1aaatCvAUfKttLearuWrP9MDH5MBPbIqV92AaeXatLxBI9gBaebbnrfifHhDYfgasaacH8akY=wiFfYdH8Gipec8Eeeu0xXdbba9frFj0=OqFfea0dXdd9vqai=hGuQ8kuc9pgc9s8qqaq=dirpe0xb9q8qiLsFr0=vr0=vr0dc8meaabaqaciaacaGaaeqabaqabeGadaaakeaaiiGacqWF7oaBdaWgaaWcbaGaemOCaiNaemyzauMaemiBaWgabeaakiabdkhaYnaaBaaaleaacqWG0baDaeqaaOGaeyypa0JaeCiuaaLaeiOla4YaaeWaaeaacqWHnbqtdaqhaaWcbaGaem4zaCMaemyzaugabaGaemOCaiNaemyzauMaemiBaWgaaOGaeCOuaifacaGLOaGaayzkaaGaaCzcaiaaxMaadaqadaqaaGqaaiab+feabjabiAda2aGaayjkaiaawMcaaaaa@48A2@

After some algebra, this yields equation (23).

## Appendix B: calculating recurrence risks for twins

The sibling recurrence risk λ_sib _is often available directly from familial studies. For twins the recurrence risks, if not reported, may be calculated from the case-wise concordance (Cc):

*λ*_*MZ *_= *Cc*_*MZ *_/*r*_*t *_    (B1)

*λ*_*DZ *_= *Cc*_*DZ *_/*r*_*t *_    (B2)

where, if there is complete ascertainment of all affected twins in a population,

*Cc *= 2*C*/(2*C *+ *D*)     (B3)

and C is the number of concordant and D the number of discordant pairs [25].

**Table 7:** Input variables

**Condition**	**λ**_**MZ**_	**λ**_**DZ**_	**λ**_**sib**_	**ε**	**PAF**^**E**^_**e**_	**r**_**t**_
Breast cancer	4.09	2.51	2.01	0.62	0.15	0.036

Lung cancer	6.27	6.14	3.16	0.15	0.86	0.017

Schizophrenia	52.1	14.2	8.6	0.62	0.15	0.01
					
				0.15	0.86	

## Supplementary Material

Additional File 1 Gene-gene and gene-environment interaction model. Contains the Visual Basic macro (Twincal), input and output datasheets and charts used to calculate the solutions described in the text. The program is run by entering parameters in the 'Inputs' sheet and clicking on the 'Run' button. Note that for the final chart ('fe') the number of categories on the horizontal axis changes depending on the environmental input parameters ε and PAF^E^_e_. If these parameters are changed it is therefore necessary to delete the lower part of the output sheet prior to running the model and, after the run, to redraw the chart using the source data option from the chart. All other charts are drawn automatically. The line γ_max _= γ_min _is calculated exactly for the chart 'fe' but is approximated in the charts 'pgdz' and 'pgdzneg' using Newton's method and an initial guess for f_ge _(f0) and step (fet). For some input parameters it may be necessary to change these values by editing the Visual Basic code (Twincal) to obtain a valid solution. Click here for file

Additional File 2 Supplementary Figure 1: Example model solution space with R_MD _= 1.7 and U_ge _≥ 0. Model solution space with U_ge _≥ 0 for the same input parameters as Figure 2, apart from λ_MZ _= 4.4. Click here for file

Additional File 3 Supplementary Figure 2: Example model solution space with R_MD _= 1.8 and U_ge _≥ 0. Model solution space with U_ge _≥ 0 for the same input parameters as Figure 2, apart from λ_MZ _= 4.6. Click here for file

Additional File 4 Supplementary Figure 3: Example model solution space with R_MD _= 1.95 and U_ge _≥ 0. Model solution space with U_ge _≥ 0 for the same input parameters as Figure 2, apart from λ_MZ _= 4.9. Click here for file

Additional File 5 Supplementary Figure 4: Example model solution space with R_MD _= 2.1 and U_ge _≥ 0. Model solution space with U_ge _≥ 0 for the same input parameters as Figure 2, apart from λ_MZ _= 5.2. Click here for file

Additional File 6 Supplementary Figure 5: Example full solution space with R_MD _= 1.7. Full model solution space for the same input parameters as Figure 5, transformed so that f_ge _is on the horizontal axis. Click here for file

Additional File 7 Supplementary Figure 6: Example full solution space with R_MD _= 1.8. Full model solution space for the same input parameters as Figure 6, transformed so that f_ge _is on the horizontal axis. Click here for file

Additional File 8 Supplementary Figure 7: Example full solution space with R_MD _= 1.95. Full model solution space for the same input parameters as Figure 7, transformed so that f_ge _is on the horizontal axis. Click here for file

Additional File 9 Supplementary Figure 8: Example full solution space with R_MD _= 2.1. Full model solution space for the same input parameters as Figure 8, transformed so that f_ge _is on the horizontal axis. Click here for file

Additional File 10 Supplementary Figure 9: Example model solution with c_MD _> 1 and U_ge _≥ 0. Input parameters: λ_MZ _= 5.2, λ_DZ _= 3, λ_sib _= 2, ε = 0.2, PAF^E^_e _= 0.5, c_MD _= 2, r_t _= 0.1. Click here for file

Additional File 11 Supplementary Figure 10: Example model solution with c_MD _> 1 and U_ge _≥ 0. Input parameters as for Figure 13. Click here for file

Additional File 12 Supplementary Figure 11: Example full solution space with c_MD _> 1. Full model solution space for the same parameters as Figure 13, transformed so that f_ge _is on the horizontal axis. Click here for file

Additional File 13 Supplementary Figure 12: Breast cancer solution space with U_ge _≥ 0. Input parameters are as shown in Table 5, with c_MD _= 1. The solution space is shown (shaded) for positive f_ge_, assuming the 'equal environments' assumption holds (c_MD _= 1). The darker shaded area shows the part of the solution space for which γ_maxge _< 1/2. Utility U_ge _is at its maximum when γ = 1/2 except within this darker shaded area. Click here for file

Additional File 14 Supplementary Figure 13: Breast cancer variances with f_ge _= 0. Input parameters as for Figure 16. Additive model of G-E interaction (f_ge _= 0). Variance components are genetic (V_g_) or environmental (V_e_). Click here for file

Additional File 15 Supplementary Figure 14: Breast cancer variances with f_ge _= 1. Input parameters as for Figure 16. Multiplicative G-E interaction model (f_ge _= 1). Variance components are genetic (V_g_), environmental (V_e_) or due to gene-environment interaction (V_ge_). Click here for file

Additional File 16 Supplementary Figure 15: Breast cancer variances with f_ge _= 1/PAF^E^_e_. Input parameters as for Figure 16. Maximum G-E interaction model (f_ge _= 1/PAF^E^_e_). Variance components are genetic (V_g_), environmental (V_e_) or due to gene-environment interaction (V_ge_). Click here for file

Additional File 17 Supplementary Figure 16: Breast cancer γ values with f_ge _= 0. Input parameters as for Figure 16. The proportion of the population in the 'high genotypic risk' group, γ, may take any value in the shaded area. γ_min _occurs when R_ge _= 1, i.e. when the Positive Predictive Value (PPV) of being in the 'ge' subgroup is 100%. γ_max _occurs when R_oo _= 1 for an additive G-E model and solutions with a Population Impact of 100% (PI = 1) cannot exist. Click here for file

Additional File 18 Supplementary Figure 17: Breast cancer γ values with f_ge _= 1. Input parameters as for Figure 16. The proportion of the population in the 'high genotypic risk' group, γ, may take any value in the shaded area. A solution with a Population Impact of 100% (PI = 1) may exist if γ = γ_max_. Click here for file

Additional File 19 Supplementary Figure 18: Breast cancer γ values with f_ge _= 1/PAF^E^_e_. Input parameters as for Figure 16. The proportion of the population in the 'high genotypic risk' group, γ, may take any value in the shaded area. A solution with a Population Impact of 100% (PI = 1) may exist if γ = γ_max_. Click here for file

Additional File 20 Supplementary Figure 19: Breast cancer solution space with U_ge _≤ 0. Input parameters are as for Figure 16. The solution space is shown for negative f_ge _(where the utility of targeting environmental interventions at the high genotypic risk group is negative, U_ge _≤ 0). Solutions exist only in the shaded area where γ_max _≥ γ_min_. Click here for file

Additional File 21 Supplementary Figure 20: Breast cancer: full solution space. Input parameters are as for Figure 16. The same solution space as Figures 16 and 23 is shown (shaded), transformed so that the G-E interaction factor is plotted on the horizontal axis. Again, each point in the shaded solution space represents a genetic model defined by p^DZ^_g _and a G-E interaction model defined by f_ge_. The area of solutions with γ_maxge _< 1/2 is highlighted with darker shading. The classical twin study solution lies on the vertical axis (f_ge _= 0) at the point p^DZ^_g _= 1/2, and is slightly outside the solution space. Click here for file

Additional File 22 Supplementary Figure 21: Lung cancer solution space with U_ge _≥ 0. Input parameters are as shown in Table 5, with c_MD _= 1. Click here for file

Additional File 23 Supplementary Figure 22: Lung cancer variances with f_ge _= 0. Input parameters as for Figure 25. Note that the horizontal axis has been expanded to show high values of c_SD_/R_SD _only. Click here for file

Additional File 24 Supplementary Figure 23: Lung cancer variances with f_ge _= 1. Input parameters as for Figure 25. Note that the horizontal axis has been expanded to show high values of c_SD_/R_SD _only. Click here for file

Additional File 25 Supplementary Figure 24: Lung cancer variances with f_ge _= 1/PAF^E^_e_. Input parameters as for Figure 25. Note that the horizontal axis has been expanded to show high values of c_SD_/R_SD _only. Click here for file

Additional File 26 Supplementary Figure 25: Lung cancer γ values for f_ge _= 1. Input parameters as for Figure 25. The proportion of the population in the 'high genotypic risk' group, γ, may take any value in the shaded area. Click here for file

Additional File 27 Supplementary Figure 26: Lung cancer γ values for f_ge _= 1/PAF^E^_e_. Input parameters as for Figure 25. The proportion of the population in the 'high genotypic risk' group, γ, may take any value in the shaded area. Click here for file

Additional File 28 Supplementary Figure 27: Lung cancer U_ge _values for f_ge _= 1. Input parameters as for Figure 25. The utility parameter, U_ge_, may take any value in the shaded area, but is maximum when γ = 1/2. Click here for file

Additional File 29 Supplementary Figure 28: Lung cancer U_ge _values for f_ge _= 1/PAF^E^_e_. Input parameters as for Figure 25. The utility parameter, U_ge_, may take any value in the shaded area, but is maximum when γ = 1/2. Click here for file

Additional File 30 Supplementary Figure 29: Lung cancer: full solution space. Input parameters as for Figure 25. Click here for file

Additional File 31 Supplementary Figure 30: Schizophrenia U_ge _≥ 0, small environmental variance and c_MD _≥ 1. Input parameters are as shown in Table 5, with ε = 0.62, PAF^E^_e _= 0.15 and c_MD _= 1. Click here for file

Additional File 32 Supplementary Figure 31: Schizophrenia U_ge _≥ 0, small environmental variance and c_MD _> 1. Input parameters are as shown in Table 5, with ε = 0.62, PAF^E^_e _= 0.15 and c_MD _= 3.8. Click here for file

Additional File 33 Supplementary Figure 32: Schizophrenia U_ge _≥ 0, large environmental variance and c_MD _= 1. Input parameters are as shown in Table 5, with ε = 0.15, PAF^E^_e _= 0.86 and c_MD _= 1. Click here for file

## References

[B1] Collins FS (1999). Shattuck Lecture – medical and societal consequences of the Human Genome Project. New Engl J Med.

[B2] Bell J (1998). The new genetics in clinical practice. BMJ.

[B3] Collins FS, McKusick VA (2001). Implications of the Human Genome Project for medical science. J Am Med Assoc.

[B4] Strohman RC (1997). The coming Kuhnian revolution in biology. Nat Biotechnol.

[B5] Holtzman NA, Marteau TM (2000). Will genetics revolutionize medicine?. New Engl J Med.

[B6] Vineis P, Schulte P, McMichael AJ (2001). Misconceptions about the use of genetic tests in populations. Lancet.

[B7] Baird P (2001). The Human Genome Project, genetics and health. Community Genet.

[B8] Cooper RS, Psaty BM (2003). Genetics and medicine: distraction, incremental progress, or the dawn of a new age?. Ann Intern Med.

[B9] Vineis P, Ahsan H, Parker M (2004). Genetic screening and occupational and environmental exposures. Occup Environ Med.

[B10] Khoury MJ, Yang Q, Gwinn M, Little J, Flanders WD (2004). An epidemiologic assessment of genetic profiling for measuring susceptibility to common diseases and targeting interventions. Genet Med.

[B11] Terwilliger JD, Weiss KM (2003). Confounding, ascertainment bias, and the blind quest for a genetic 'fountain of youth'. Ann Med.

[B12] Ioannidis JPA, Ntzani EE, Trikalinos TA, Contopoulos-Ionnidis DG (2001). Replication validity of genetic association studies. Nat Genet.

[B13] Cordell HJ, Clayton DG (2005). Genetic association studies. Lancet.

[B14] Ioannidis J (2005). Why most published research findings are false. PloS Med.

[B15] Layzer D (1974). Heritability analyses of IQ scores: science or numerology?. Science.

[B16] Risch N (1990). Linkage strategies for genetically complex traits. I. Multilocus models. Am J Hum Genet.

[B17] Guo S-W (2000). Gene-environment interaction and the mapping of complex traits: some statistical models and their interpretation. Hum Hered.

[B18] Hopper JL, Spector TD, Sneider H, MacGregor AJ (2000). Why 'common environmental effects' are so uncommon in the literature. Advances in twin and sib-pair analysis.

[B19] Khoury MJ, Wagener DK (1995). Epidemiological evaluation of the use of genetics to improve the predictive value of disease risk factors. Am J Hum Genet.

[B20] Lewis SJ, Brunner EJ (2004). Methodological problems in genetic association studies of longevity – the apolipoprotein E gene as an example. Int J Epidemiol.

[B21] Tryggvadottir L, Sigvaldason H, Olafsdottir GH, Jonasson JG, Jonsson T, Tulinius H, Eyfjord JE (2006). Population-based study of changing breast cancer risk in Icelandic BRCA2 mutation carriers, 1920–2000. J Natl Cancer Inst.

[B22] Humphries S, Ridker PM, Talmud PJ (2004). Genetic testing for cardiovascular disease susceptibility: a useful clinical management tool or possible misinformation?. Arterioscler Thromb Vasc Biol.

[B23] Rose G (1985). Sick individuals and sick populations. Int J Epidemiol.

[B24] Khoury MJ, Jones K, Grosse SD (2006). Quantifying the health benefits of genetic tests: The importance of a population perspective. Genet Med.

[B25] MacGregor AJ, Spector TD, Sneider H, MacGregor AJ (2000). Practical approaches to account for bias and confounding in twin data. Advances in twin and sib-pair analysis.

[B26] Fisher RA (1918). The correlation between relatives on the supposition of Mendelian inheritance. Trans R Soc Edinb.

[B27] Hopper JL (1993). Variance components for statistical genetics: applications in medical research to characteristics related to human diseases and health. Stat Methods Med Res.

[B28] Porteous JW (2004). A rational treatment of Mendelian genetics. Theor Biol Med Model.

[B29] Azevedo RBR, Lohaus R, Srinivasan S, Dang KK, Burch CL (2006). Sexual reproduction selects for robustness and negative epistasis in artificial gene networks. Nature.

[B30] Risch N (2001). The genetic epidemiology of cancer: interpreting family and twin studies and their implications for molecular genetic approaches. Cancer Epidemiol Biomarkers Prev.

[B31] Lichtenstein P, Holm NV, Verkasalo PK, Iliadou A, Kaprio J, Koskenvuo M, Pukkala E, Skytthe A, Hemminki K (2000). Environmental and heritable factors in the causation of cancer. New Engl J Med.

[B32] Dong C, Hemminki K (2001). Modification of cancer risks in offspring by sibling and parental cancers from 2,112,616 nuclear families. Int J Cancer.

[B33] Rockhill B, Weinberg CR, Newman B (1998). Population attributable fraction estimation for established breast cancer risk factors: considering the issues of high prevalence and unmodifiability. Am J Epidemiol.

[B34] McGue M, Gottesman II, Rao DC (1983). The transmission of schizophrenia under a multifactorial threshold model. Am J Hum Genet.

[B35] Easton DF (1999). How many more breast cancer predisposition genes are there?. Breast Cancer Res.

[B36] Badano JL, Katsanis N (2002). Beyond Mendel: an evolving view of human genetic disease transmission. Nat Rev Genet.

[B37] Badano JL, Leitch CC, Ansley SJ, May-Simera H, Lawson S, Lewis RA, Beales PL, Dietz HC, Fisher S, Katsanis N (2006). Dissection of epistasis in oligogenic Bardet-Biedl syndrome. Nature.

[B38] Taioli E, Zocchetti C, Garte S (1998). Models of interaction between metabolic genes and environmental exposure in cancer susceptibility. Environ Health Perspect.

[B39] Hunter DJ (2005). Gene-environment interactions in human diseases. Nat Rev Genet.

[B40] Antoniou AC, Pharoah PDP, McMullan G, Day NE, Stratton MR, Peto J, Ponder BJ, Easton DF (2002). A comprehensive model for familial breast cancer incorporating BRCA1, BRCA2 and other genes. Br J Cancer.

[B41] Braun MM, Caporaso NE, Page WF, Hoover RN (1995). A cohort study of twins and cancer. Cancer Epidemiol Biomarkers Prev.

[B42] Hall W, Madden P, Lynskey M (2002). The genetics of tobacco use: methods, findings and policy implications. Tob Control.

[B43] Vink JM, Willemsen G, Boomsma DI (2003). The association of current smoking behavior with the smoking behavior of parents, siblings, friends and spouses. Addiction.

[B44] Taioli E, Garte S (2002). Covariates and confounding in epidemiologic studies using metabolic gene polymorphisms. Int J Cancer.

[B45] Guo S (1999). The behaviors of some heritability estimators in the complete absence of genetic factors. Hum Hered.

[B46] Li CC, Sacks L (1954). The derivation of joint distribution and correlation between relatives by the use of stochastic matrices. Biometrics.

