# Supplementary figures and images for: A model of gene-gene and gene-environment interactions and its implications for targeting environmental interventions by genotype
Source: Theor Biol Med Model. 2006 Oct 9;3:35. doi: 10.1186/1742-4682-3-35 (PMC1629012; doi:10.1186/1742-4682-3-35)

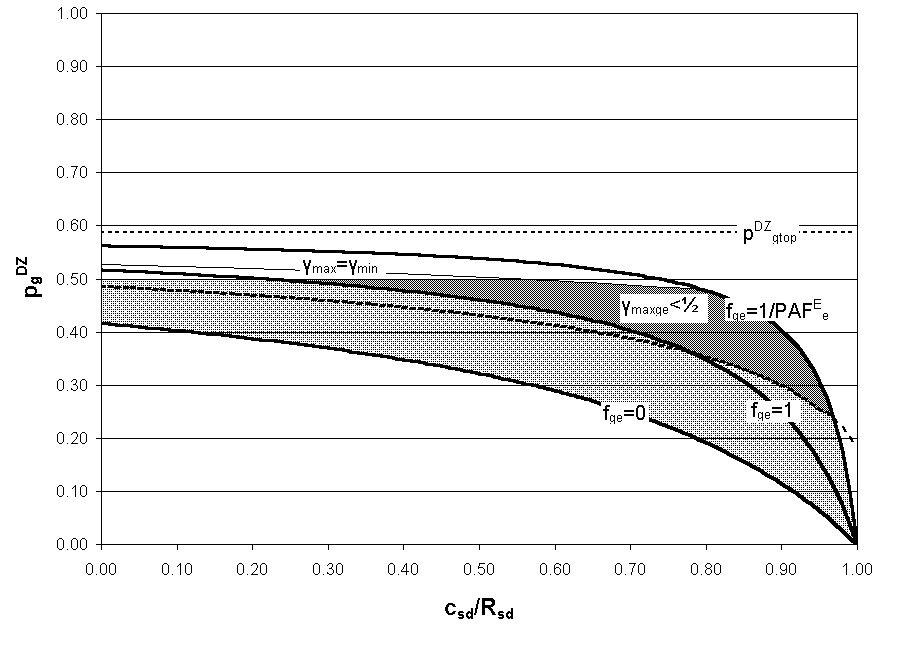

Supplement: Additional File 2 — Supplementary Figure 1: Example model solution space with RMD = 1.7 and Uge ≥ 0. Model solution space with Uge ≥ 0 for the same input parameters as Figure 2, apart from λMZ = 4.4. [file 1742-4682-3-35-S2.bmp]

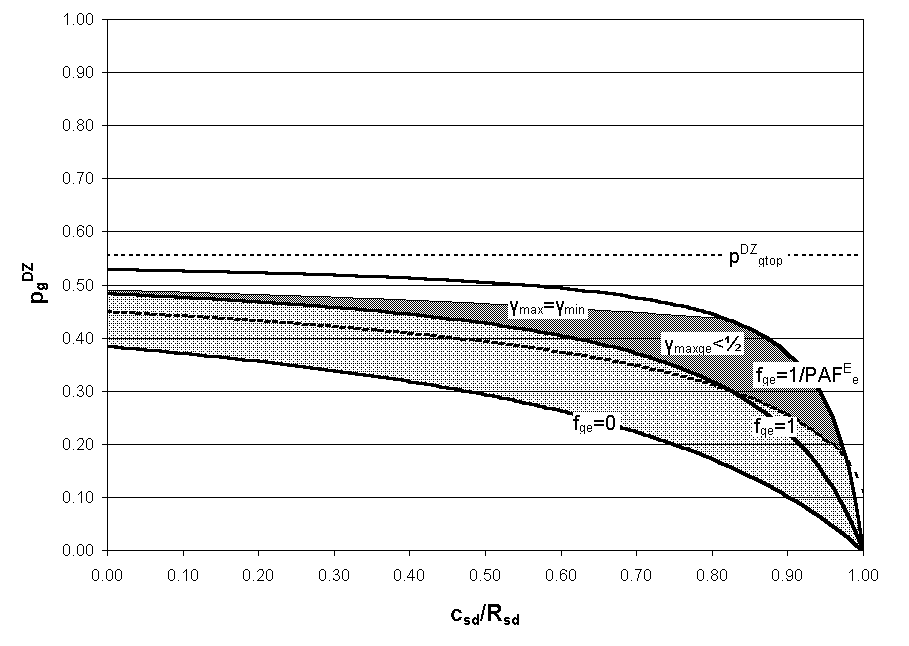

Supplement: Additional File 3 — Supplementary Figure 2: Example model solution space with RMD = 1.8 and Uge ≥ 0. Model solution space with Uge ≥ 0 for the same input parameters as Figure 2, apart from λMZ = 4.6. [file 1742-4682-3-35-S3.bmp]

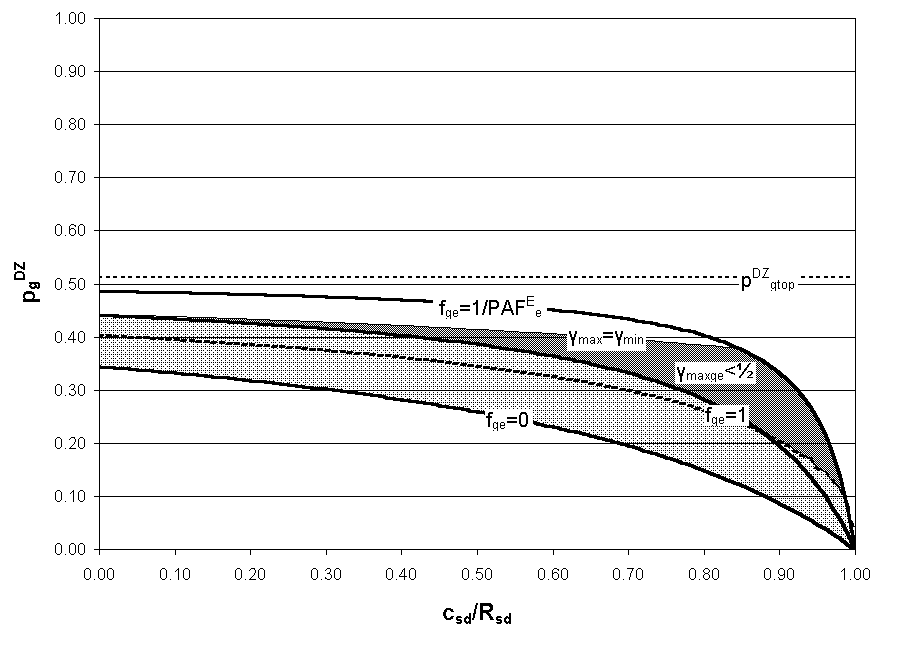

Supplement: Additional File 4 — Supplementary Figure 3: Example model solution space with RMD = 1.95 and Uge ≥ 0. Model solution space with Uge ≥ 0 for the same input parameters as Figure 2, apart from λMZ = 4.9. [file 1742-4682-3-35-S4.bmp]

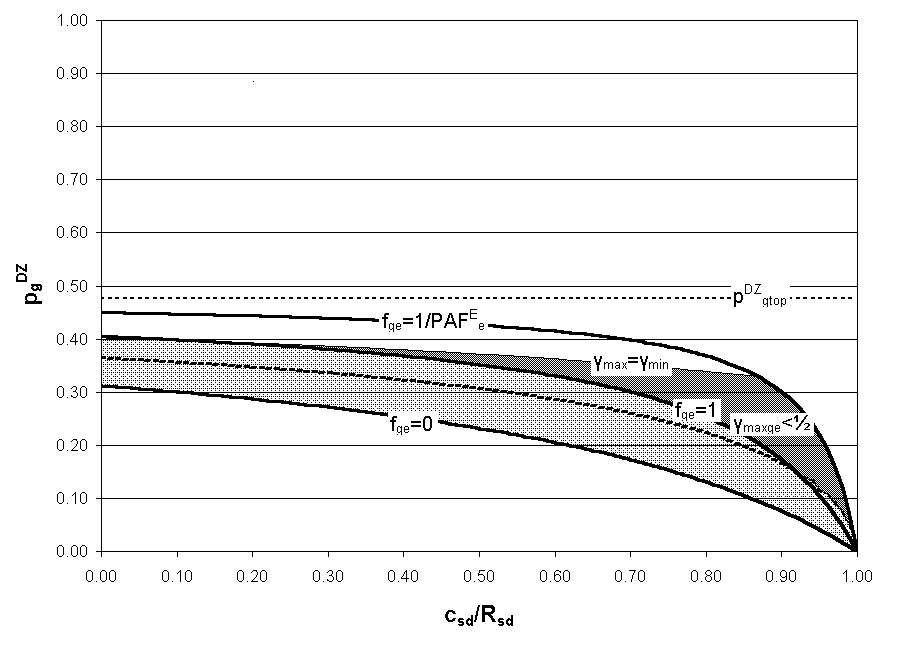

Supplement: Additional File 5 — Supplementary Figure 4: Example model solution space with RMD = 2.1 and Uge ≥ 0. Model solution space with Uge ≥ 0 for the same input parameters as Figure 2, apart from λMZ = 5.2. [file 1742-4682-3-35-S5.bmp]

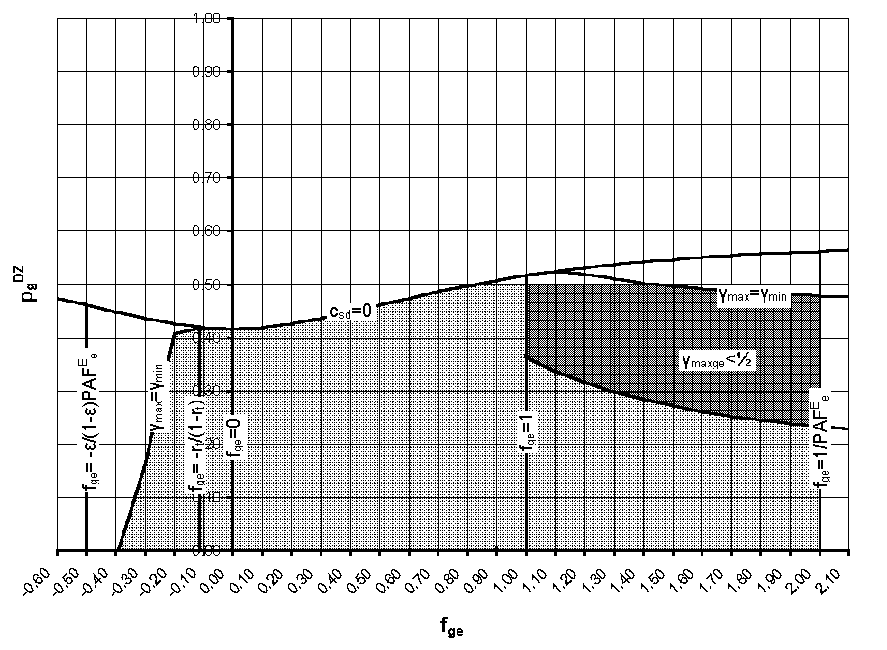

Supplement: Additional File 6 — Supplementary Figure 5: Example full solution space with RMD = 1.7. Full model solution space for the same input parameters as Figure 5, transformed so that fge is on the horizontal axis. [file 1742-4682-3-35-S6.bmp]

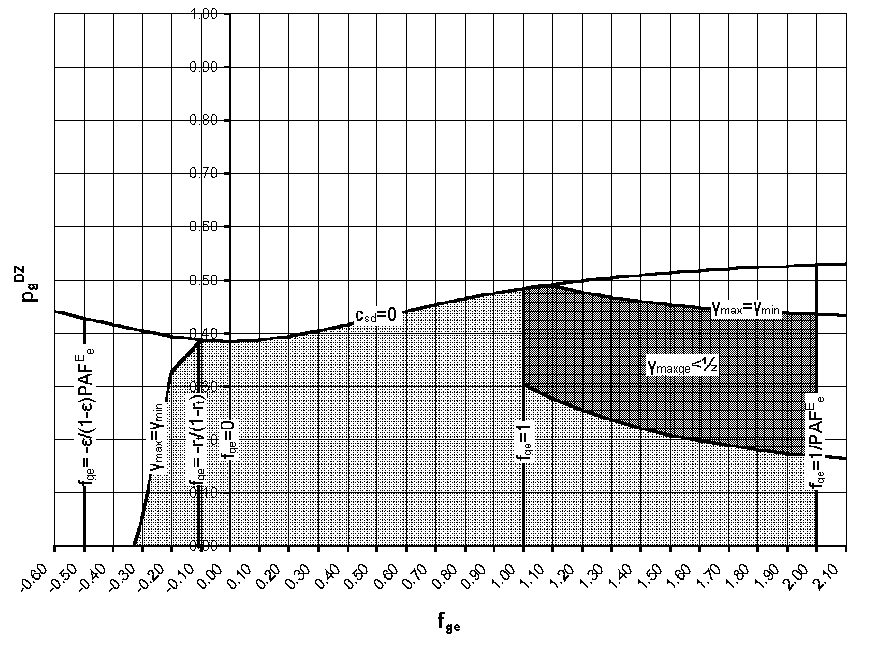

Supplement: Additional File 7 — Supplementary Figure 6: Example full solution space with RMD = 1.8. Full model solution space for the same input parameters as Figure 6, transformed so that fge is on the horizontal axis. [file 1742-4682-3-35-S7.bmp]

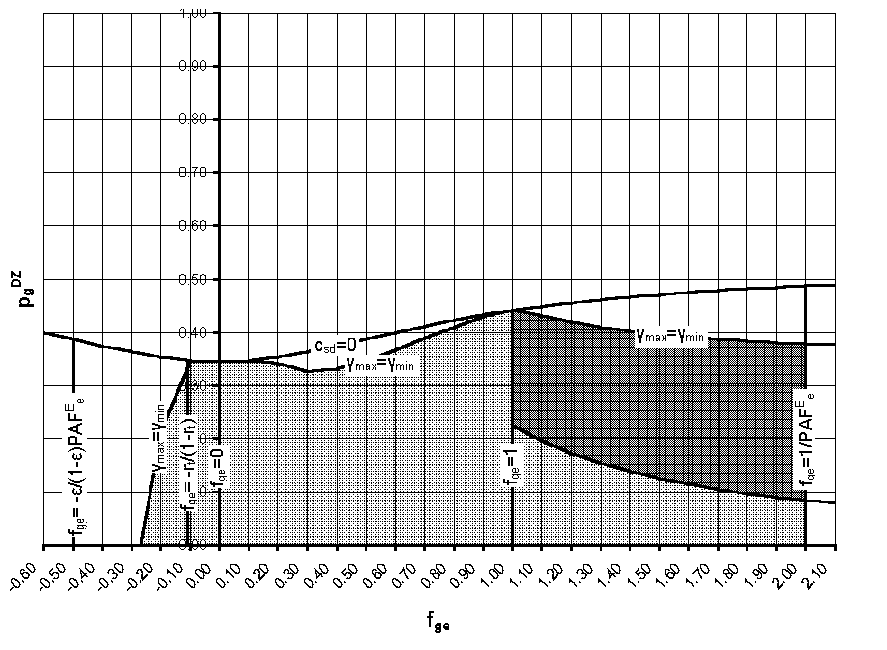

Supplement: Additional File 8 — Supplementary Figure 7: Example full solution space with RMD = 1.95. Full model solution space for the same input parameters as Figure 7, transformed so that fge is on the horizontal axis. [file 1742-4682-3-35-S8.bmp]

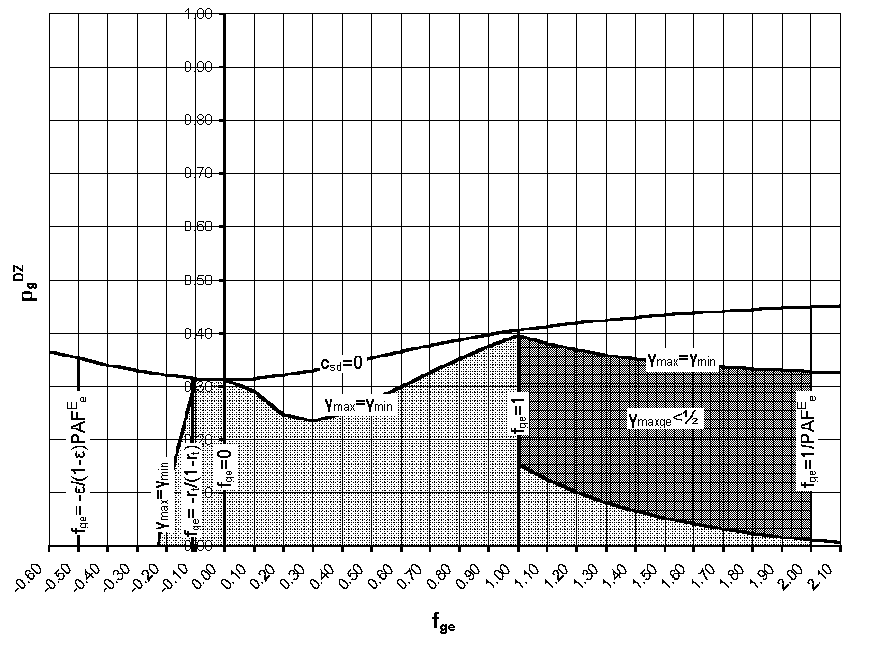

Supplement: Additional File 9 — Supplementary Figure 8: Example full solution space with RMD = 2.1. Full model solution space for the same input parameters as Figure 8, transformed so that fge is on the horizontal axis. [file 1742-4682-3-35-S9.bmp]

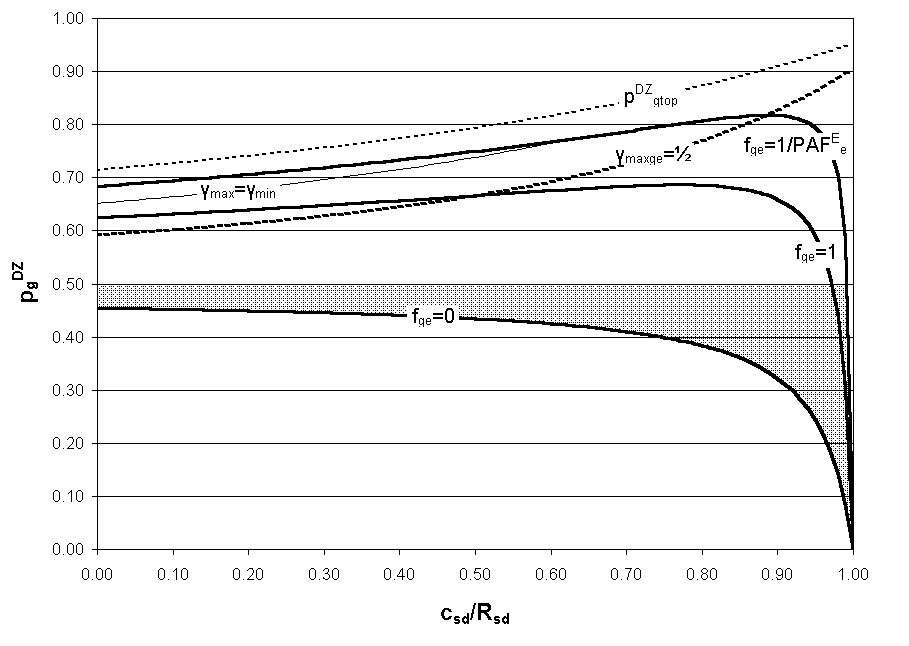

Supplement: Additional File 10 — Supplementary Figure 9: Example model solution with cMD > 1 and Uge ≥ 0. Input parameters: λMZ = 5.2, λDZ = 3, λsib = 2, ε = 0.2, PAFEe = 0.5, cMD = 2, rt = 0.1. [file 1742-4682-3-35-S10.bmp]

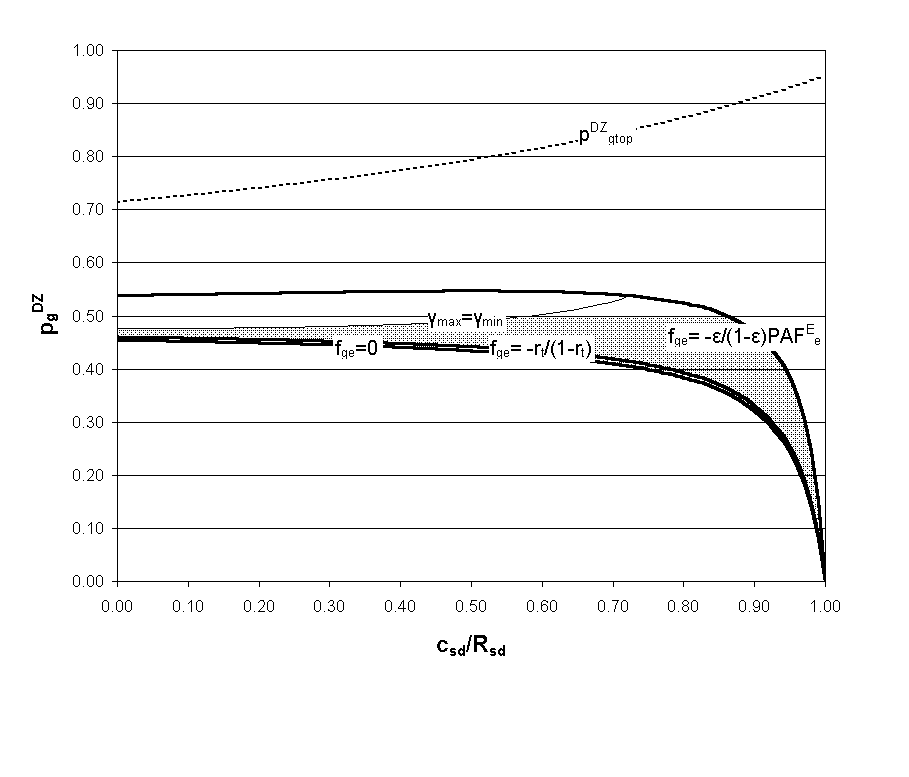

Supplement: Additional File 11 — Supplementary Figure 10: Example model solution with cMD > 1 and Uge ≥ 0. Input parameters as for Figure 13. [file 1742-4682-3-35-S11.bmp]

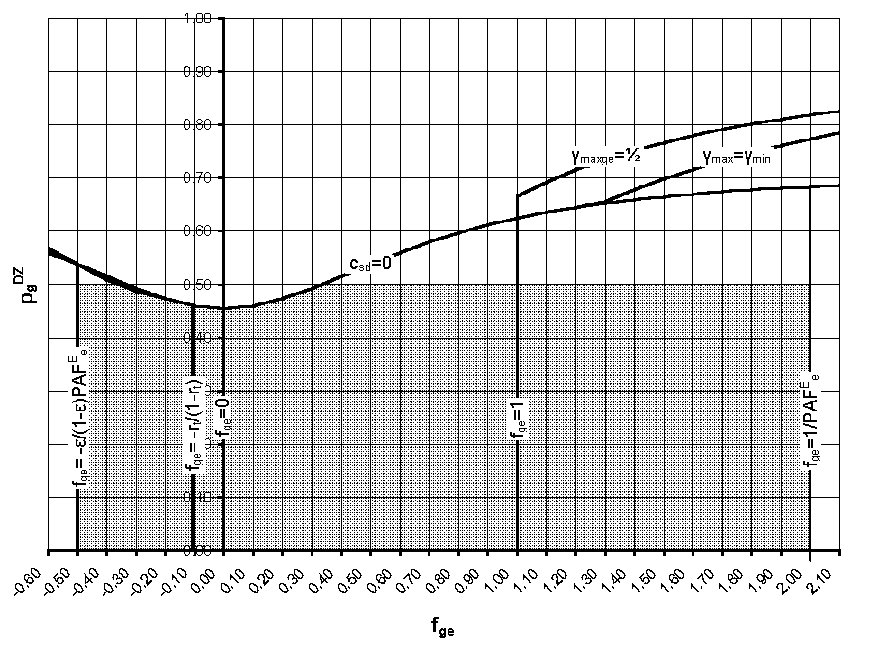

Supplement: Additional File 12 — Supplementary Figure 11: Example full solution space with cMD > 1. Full model solution space for the same parameters as Figure 13, transformed so that fge is on the horizontal axis. [file 1742-4682-3-35-S12.bmp]

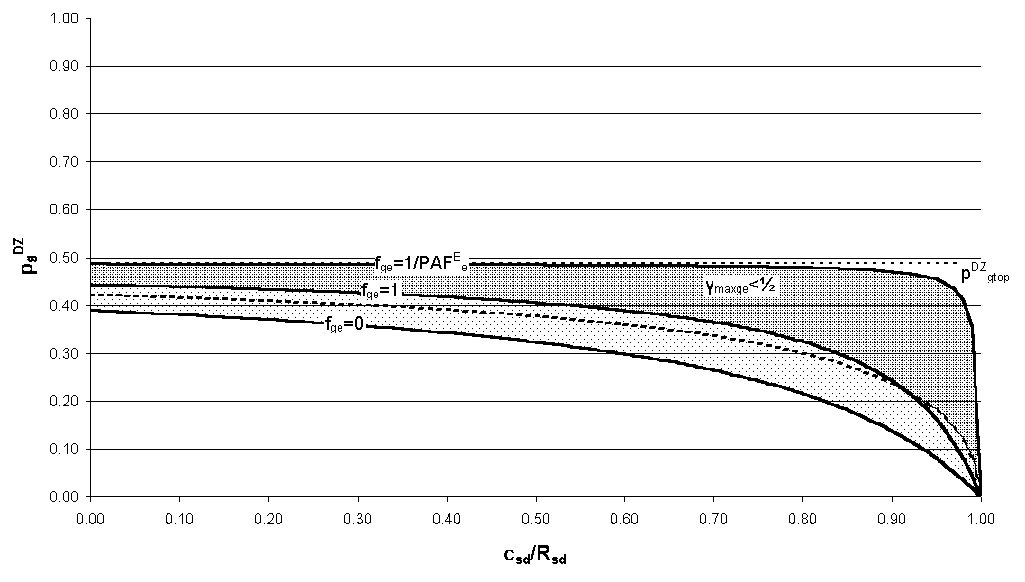

Supplement: Additional File 13 — Supplementary Figure 12: Breast cancer solution space with Uge ≥ 0. Input parameters are as shown in Table 5, with cMD = 1. The solution space is shown (shaded) for positive fge, assuming the 'equal environments' assumption holds (cMD = 1). The darker shaded area shows the part of the solution space for which γmaxge < 1/2. Utility Uge is at its maximum when γ = 1/2 except within this darker shaded area. [file 1742-4682-3-35-S13.bmp]

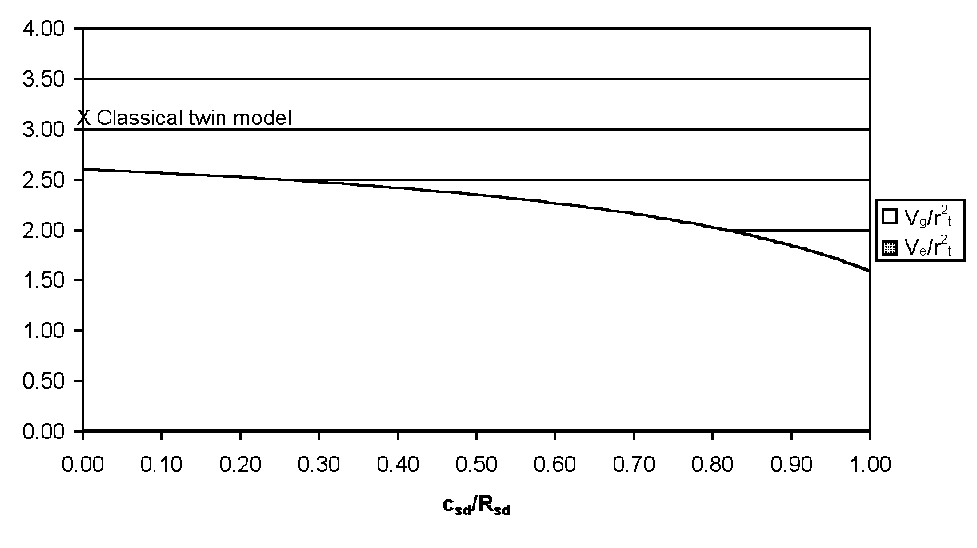

Supplement: Additional File 14 — Supplementary Figure 13: Breast cancer variances with fge = 0. Input parameters as for Figure 16. Additive model of G-E interaction (fge = 0). Variance components are genetic (Vg) or environmental (Ve). [file 1742-4682-3-35-S14.bmp]

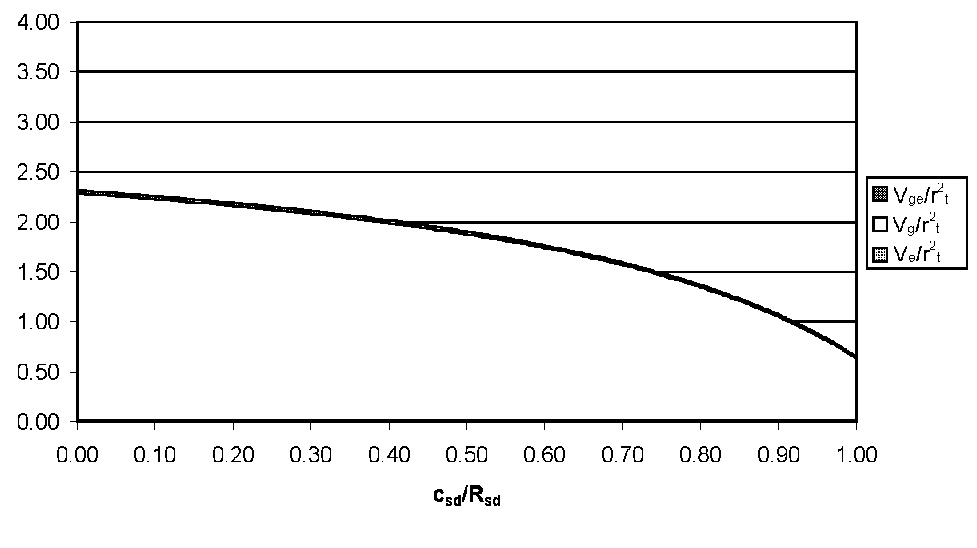

Supplement: Additional File 15 — Supplementary Figure 14: Breast cancer variances with fge = 1. Input parameters as for Figure 16. Multiplicative G-E interaction model (fge = 1). Variance components are genetic (Vg), environmental (Ve) or due to gene-environment interaction (Vge). [file 1742-4682-3-35-S15.bmp]

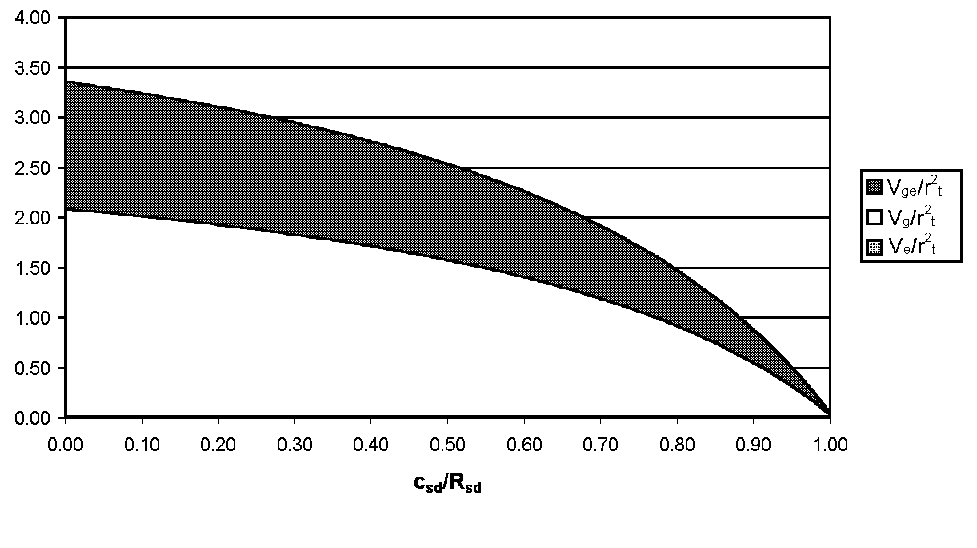

Supplement: Additional File 16 — Supplementary Figure 15: Breast cancer variances with fge = 1/PAFEe. Input parameters as for Figure 16. Maximum G-E interaction model (fge = 1/PAFEe). Variance components are genetic (Vg), environmental (Ve) or due to gene-environment interaction (Vge). [file 1742-4682-3-35-S16.bmp]

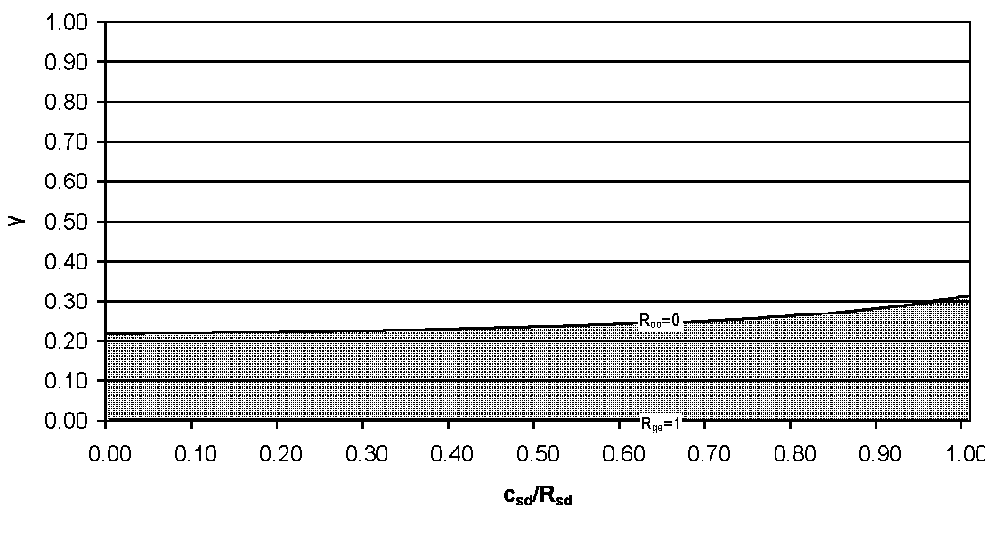

Supplement: Additional File 17 — Supplementary Figure 16: Breast cancer γ values with fge = 0. Input parameters as for Figure 16. The proportion of the population in the 'high genotypic risk' group, γ, may take any value in the shaded area. γmin occurs when Rge = 1, i.e. when the Positive Predictive Value (PPV) of being in the 'ge' subgroup is 100%. γmax occurs when Roo = 1 for an additive G-E model and solutions with a Population Impact of 100% (PI = 1) cannot exist. [file 1742-4682-3-35-S17.bmp]

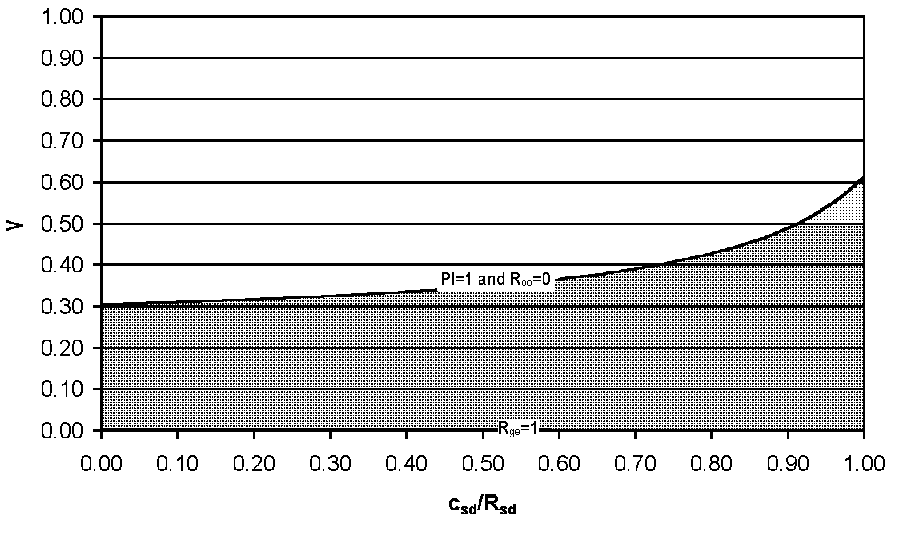

Supplement: Additional File 18 — Supplementary Figure 17: Breast cancer γ values with fge = 1. Input parameters as for Figure 16. The proportion of the population in the 'high genotypic risk' group, γ, may take any value in the shaded area. A solution with a Population Impact of 100% (PI = 1) may exist if γ = γmax. [file 1742-4682-3-35-S18.bmp]

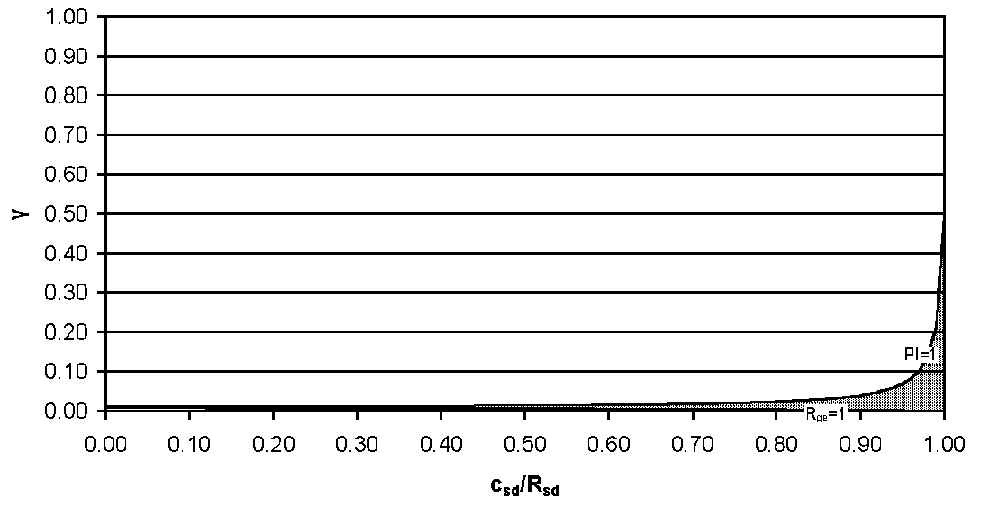

Supplement: Additional File 19 — Supplementary Figure 18: Breast cancer γ values with fge = 1/PAFEe. Input parameters as for Figure 16. The proportion of the population in the 'high genotypic risk' group, γ, may take any value in the shaded area. A solution with a Population Impact of 100% (PI = 1) may exist if γ = γmax. [file 1742-4682-3-35-S19.bmp]

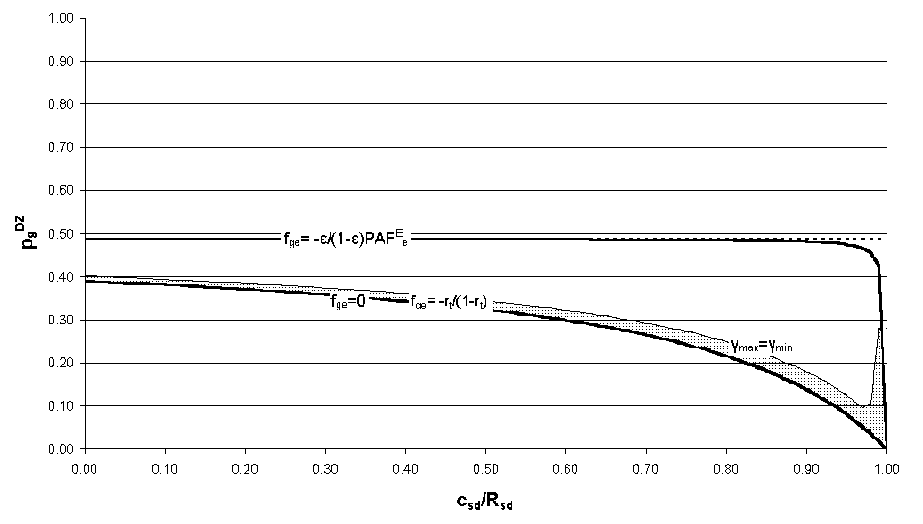

Supplement: Additional File 20 — Supplementary Figure 19: Breast cancer solution space with Uge ≤ 0. Input parameters are as for Figure 16. The solution space is shown for negative fge (where the utility of targeting environmental interventions at the high genotypic risk group is negative, Uge ≤ 0). Solutions exist only in the shaded area where γmax ≥ γmin. [file 1742-4682-3-35-S20.bmp]

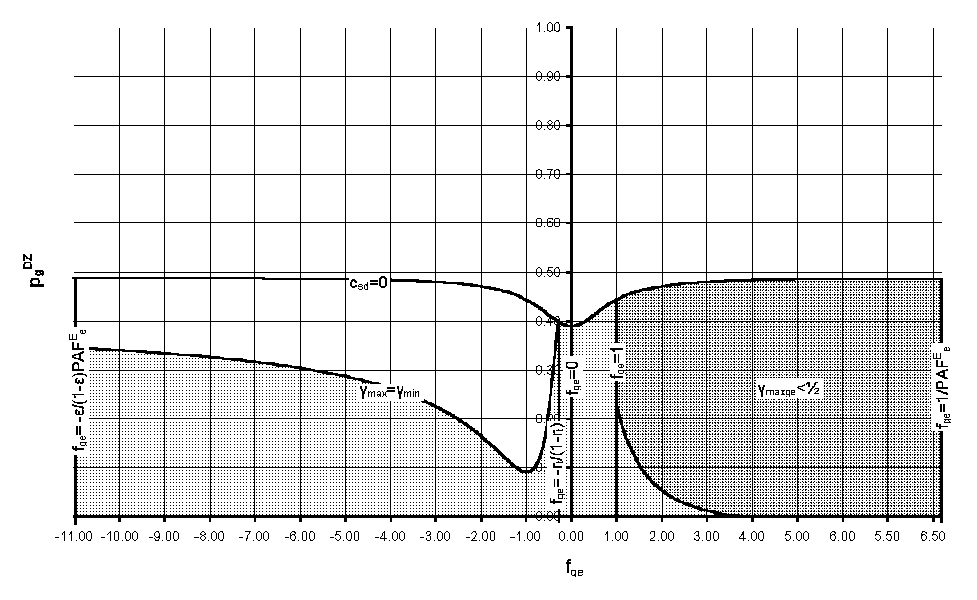

Supplement: Additional File 21 — Supplementary Figure 20: Breast cancer: full solution space. Input parameters are as for Figure 16. The same solution space as Figures 16 and 23 is shown (shaded), transformed so that the G-E interaction factor is plotted on the horizontal axis. Again, each point in the shaded solution space represents a genetic model defined by pDZg and a G-E interaction model defined by fge. The area of solutions with γmaxge < 1/2 is highlighted with darker shading. The classical twin study solution lies on the vertical axis (fge = 0) at the point pDZg = 1/2, and is slightly outside the solution space. [file 1742-4682-3-35-S21.bmp]

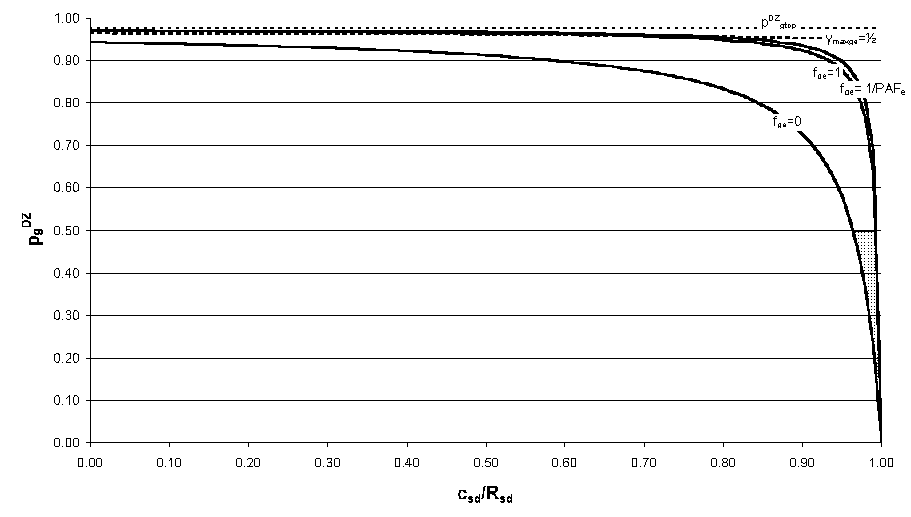

Supplement: Additional File 22 — Supplementary Figure 21: Lung cancer solution space with Uge ≥ 0. Input parameters are as shown in Table 5, with cMD = 1. [file 1742-4682-3-35-S22.bmp]

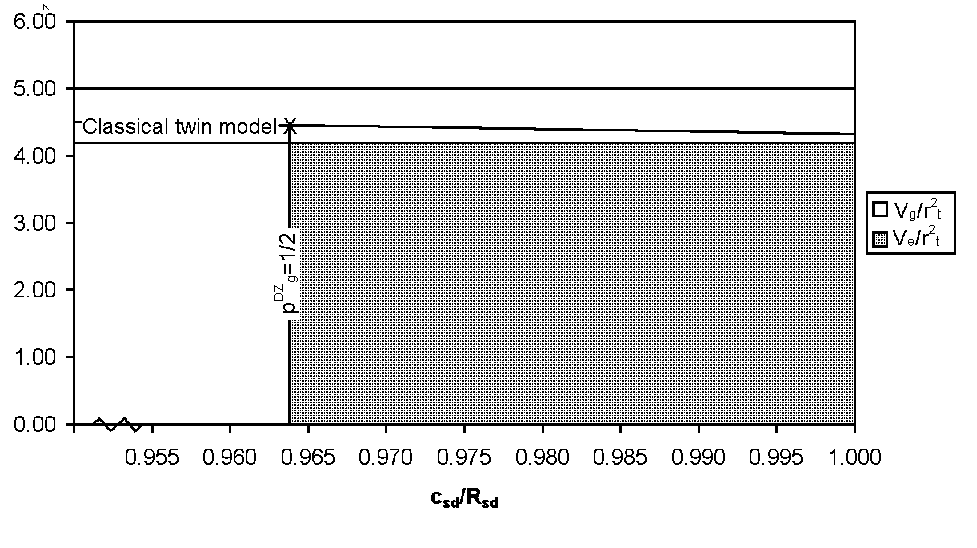

Supplement: Additional File 23 — Supplementary Figure 22: Lung cancer variances with fge = 0. Input parameters as for Figure 25. Note that the horizontal axis has been expanded to show high values of cSD/RSD only. [file 1742-4682-3-35-S23.bmp]

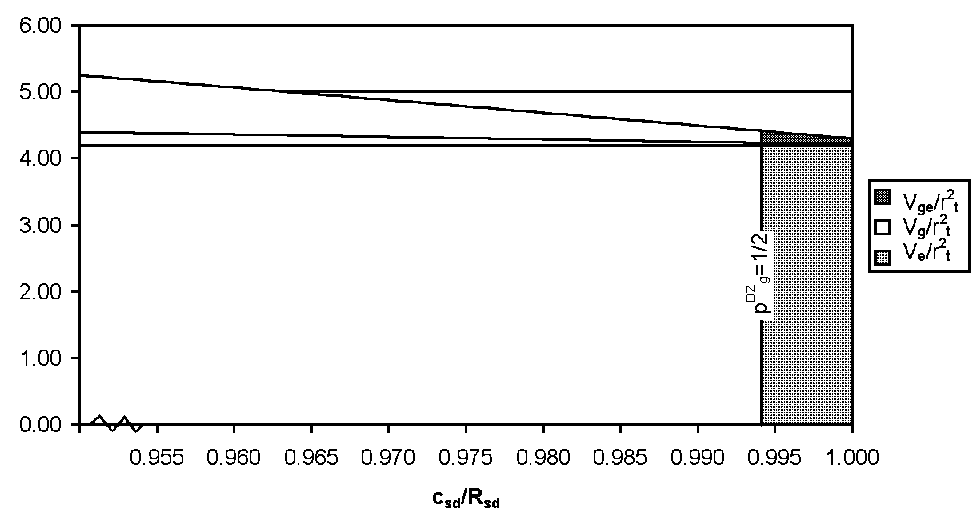

Supplement: Additional File 24 — Supplementary Figure 23: Lung cancer variances with fge = 1. Input parameters as for Figure 25. Note that the horizontal axis has been expanded to show high values of cSD/RSD only. [file 1742-4682-3-35-S24.bmp]

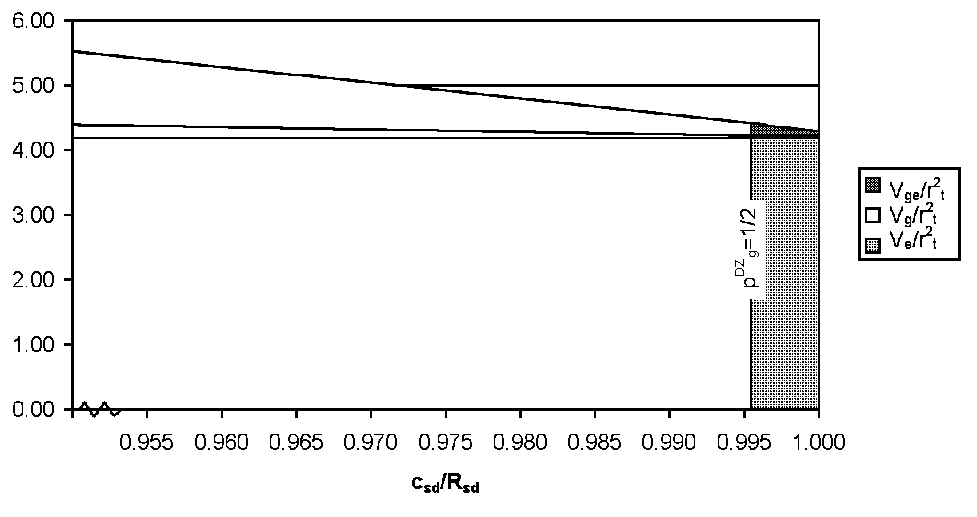

Supplement: Additional File 25 — Supplementary Figure 24: Lung cancer variances with fge = 1/PAFEe. Input parameters as for Figure 25. Note that the horizontal axis has been expanded to show high values of cSD/RSD only. [file 1742-4682-3-35-S25.bmp]

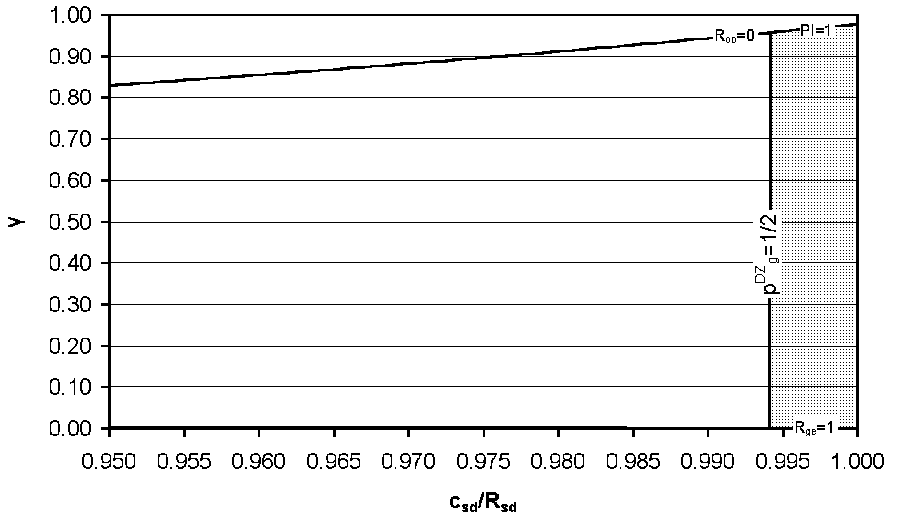

Supplement: Additional File 26 — Supplementary Figure 25: Lung cancer γ values for fge = 1. Input parameters as for Figure 25. The proportion of the population in the 'high genotypic risk' group, γ, may take any value in the shaded area. [file 1742-4682-3-35-S26.bmp]

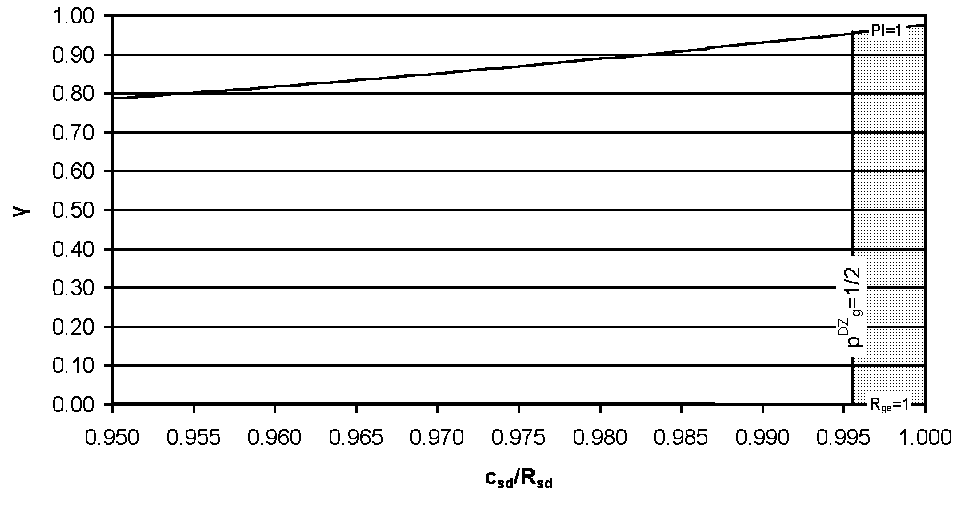

Supplement: Additional File 27 — Supplementary Figure 26: Lung cancer γ values for fge = 1/PAFEe. Input parameters as for Figure 25. The proportion of the population in the 'high genotypic risk' group, γ, may take any value in the shaded area. [file 1742-4682-3-35-S27.bmp]

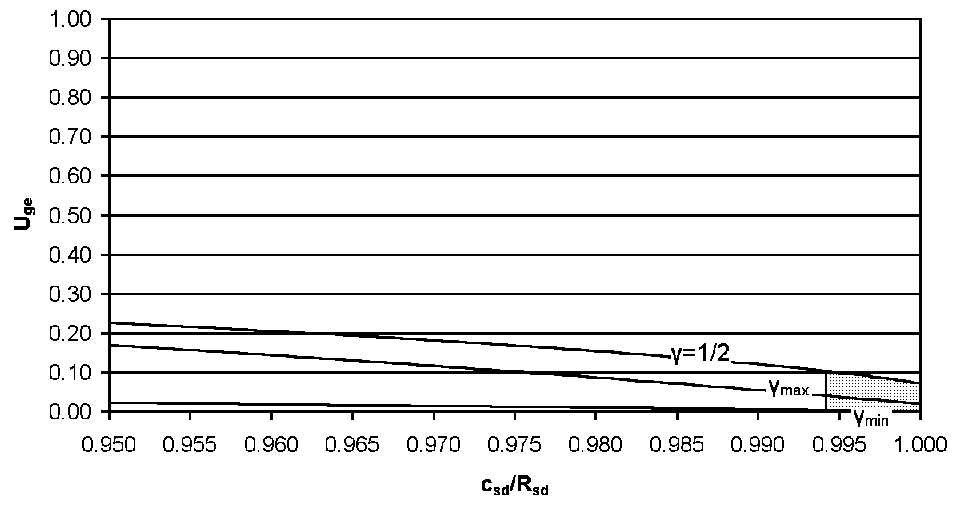

Supplement: Additional File 28 — Supplementary Figure 27: Lung cancer Uge values for fge = 1. Input parameters as for Figure 25. The utility parameter, Uge, may take any value in the shaded area, but is maximum when γ = 1/2. [file 1742-4682-3-35-S28.bmp]

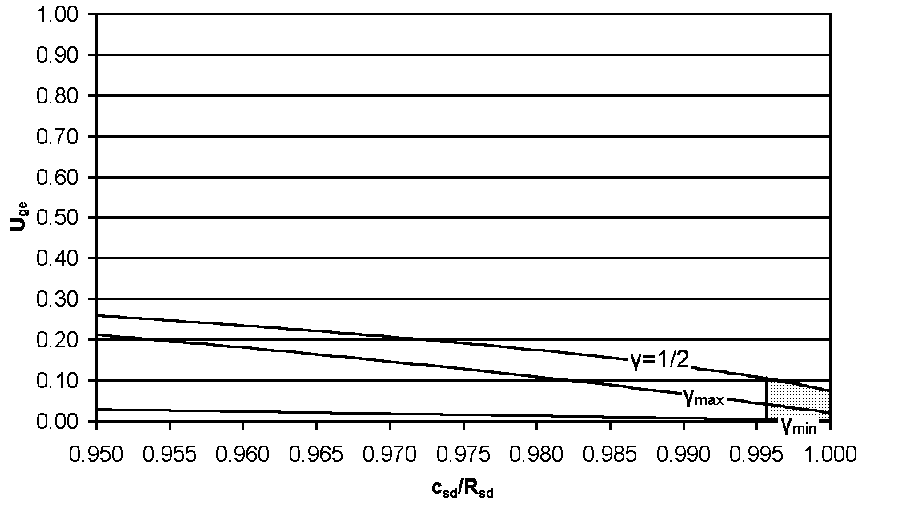

Supplement: Additional File 29 — Supplementary Figure 28: Lung cancer Uge values for fge = 1/PAFEe. Input parameters as for Figure 25. The utility parameter, Uge, may take any value in the shaded area, but is maximum when γ = 1/2. [file 1742-4682-3-35-S29.bmp]

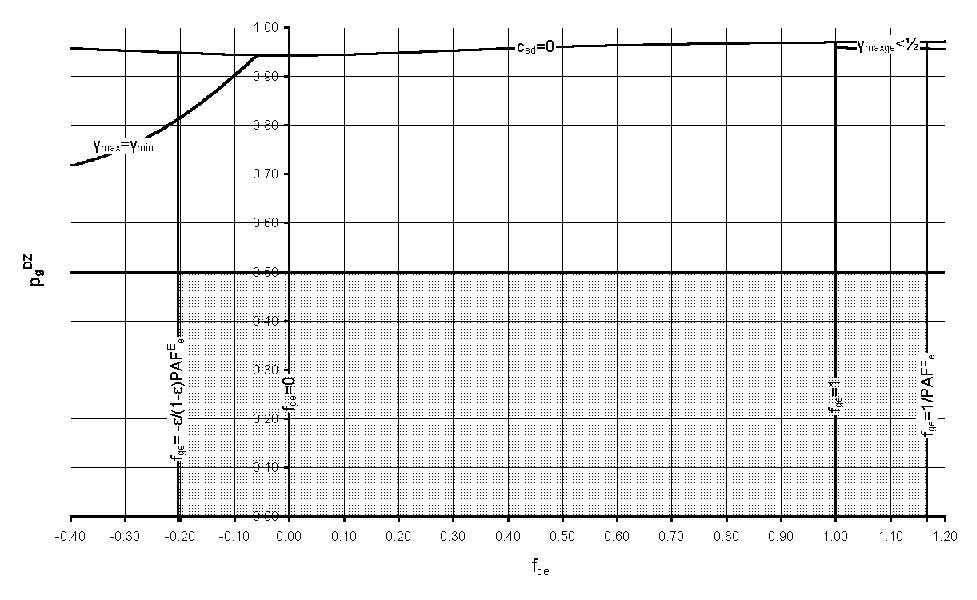

Supplement: Additional File 30 — Supplementary Figure 29: Lung cancer: full solution space. Input parameters as for Figure 25. [file 1742-4682-3-35-S30.bmp]

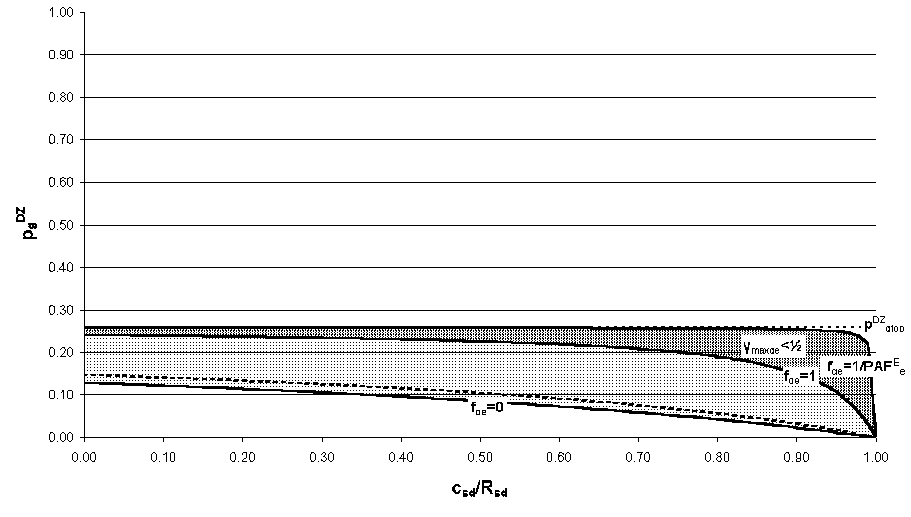

Supplement: Additional File 31 — Supplementary Figure 30: Schizophrenia Uge ≥ 0, small environmental variance and cMD ≥ 1. Input parameters are as shown in Table 5, with ε = 0.62, PAFEe = 0.15 and cMD = 1. [file 1742-4682-3-35-S31.bmp]

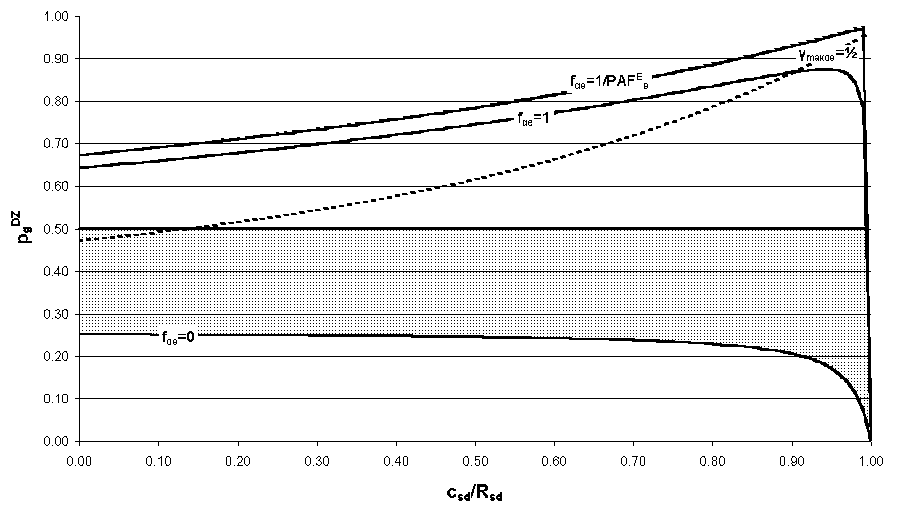

Supplement: Additional File 32 — Supplementary Figure 31: Schizophrenia Uge ≥ 0, small environmental variance and cMD > 1. Input parameters are as shown in Table 5, with ε = 0.62, PAFEe = 0.15 and cMD = 3.8. [file 1742-4682-3-35-S32.bmp]

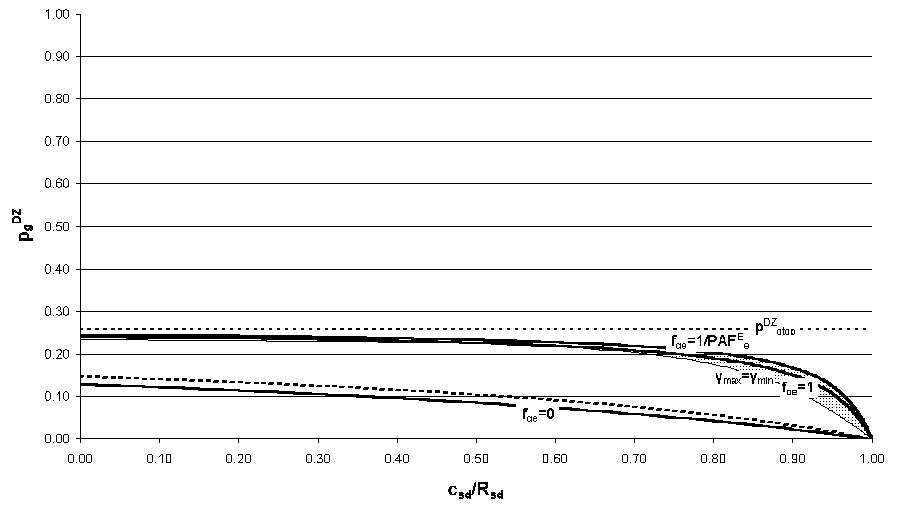

Supplement: Additional File 33 — Supplementary Figure 32: Schizophrenia Uge ≥ 0, large environmental variance and cMD = 1. Input parameters are as shown in Table 5, with ε = 0.15, PAFEe = 0.86 and cMD = 1. [file 1742-4682-3-35-S33.bmp]
